# Anti-invasive and cytotoxic evaluation of a (+)-pinoresinol-based semisynthetic library against glioblastoma

**DOI:** 10.3762/bjoc.22.54

**Published:** 2026-05-11

**Authors:** Chen Zhang, Kah Yean Lum, Jonathan M White, Paul I Forster, Nicholas Booth, Sunita A Ramesh, Rohan A Davis

**Affiliations:** 1 Institute for Biomedicine and Glycomics, Griffith University, Brisbane, QLD 4111, Australia; 2 School of Environment and Science, Griffith University, Brisbane, QLD 4111, Australia; 3 School of Chemistry and Bio21 Institute, The University of Melbourne, Melbourne, VIC 3010, Australia; 4 Queensland Herbarium & Biodiversity Assessment, Department of the Environment, Science, Tourism and Innovation, Brisbane Botanic Gardens, Toowong, QLD 4066, Australia; 5 Biological Sciences, College of Science and Engineering, Flinders University, Bedford Park, SA 5042, Australia; 6 NatureBank, Griffith University, Brisbane, QLD 4111, Australia

**Keywords:** anti-invasive, cytotoxicity, *Eremophila maculata*, glioblastoma, (+)-pinoresinol, seeds, semisynthesis

## Abstract

The Australian endemic plant genus *Eremophila* has long been recognized for its unique chemical diversity, with numerous novel and bioactive compounds reported. In this study, we chemically investigated the seeds of *Eremophila maculata* for the first time, which led to the isolation and characterization of two known plant metabolites, (+)-salicifoliol and (+)-pinoresinol. Due to the reported biological properties of the lignan natural product (+)-pinoresinol and its high abundance from the seeds of *E. maculata*, we synthesized five analogues for biological evaluations. Preliminary cytotoxicity evaluations of the semisynthetic pinoresinol-based library against two human glioblastoma cell lines, U251MG and KNS42, showed (+)-4,4'-di(3,3-dimethylbutanoyl)pinoresinol had slight cytotoxicity at 10 µM. A transwell anti-invasive assay on the same compound showed a reduction in the invasion of adult U251MG cells by 50% and pediatric KNS42 cells by 30%, with IC_50_ values of 0.26 and 0.40 µM, respectively.

## Introduction

Since ancient times, natural products have played a crucial role in the development of medicines for various diseases [1–4]. A recent review by Newman et al. has identified that over the past four decades, >30% of therapeutic drugs approved by the FDA are derived from natural products, their derivatives, or synthetic mimetics inspired by natural compounds [5]. The traditional knowledge of native plants for medicinal purposes is invaluable, and there are ongoing investigations by many research groups from around the world that aim to scientifically validate these traditional remedies and explore their potential applications in modern medicine. Australia's native flora, which is renowned for its rich biodiversity, has been used by Australian Aboriginal peoples for thousands of years, and they have long used *Eremophila* plants such as *E. longifolia* and *E. alternifolia* as traditional medicines [6–8]. In recent times, *Eremophila* plants have been shown to generate impressive chemical diversity, along with a wide range of biological activities from both extracts or isolated natural products, such as antibacterial, antimalarial, and anticancer effects [9–14]. However, many of the 287 species that make up this genus have not yet been chemically investigated, indicating that *Eremophila* still has the potential to be the source of new bioactive chemicals and/or known compounds with new biological activity. The use of *Eremophila*-derived specialized metabolites or semisynthetic derivatives has the potential to impact biodiscovery research.

In this study, we investigated the chemistry of the seeds of *Eremophila maculata* for the first time. Seeds from the *Eremophila* genus have rarely been chemically investigated due to difficulties in collecting sufficient quantities of this plant part. Owing to access to Griffith University’s NatureBank biota repository, which contains 45 locally collected *Eremophila* samples, we were able to obtain more than 20 g of *E. maculata* seeds. Large-scale extraction and isolation studies on this uniquely abundant plant part afforded two known phytochemicals, (+)-salicifoliol (**1**) [15–16] and (+)-pinoresinol (**2**) [17]. The abundant natural product **2** was used as a chemical scaffold for semisynthetic studies. At the same time, all derivatives were fully characterized utilizing spectroscopic and spectrometric techniques.

Plants are a vast reservoir of phytochemicals that serve as the source of numerous drugs, including many with anticancer properties [18–19]. These phytochemicals may act on cellular signalling, apoptosis, metabolic pathways, and cell motility in tumor cells. Most anticancer research focuses on antiproliferation or apoptosis of tumor cells. However, tumor cells are highly aggressive and have the capacity to invade and metastasize from a primary tumor to other parts of the body. Strategies that can limit or inhibit the invasiveness of tumor cells without damaging or killing healthy cells represent a powerful approach that can augment current therapies, prevent metastasis, and improve patient quality of life and survival. Cytotoxicity is defined as the ability of a compound to cause cell death, while anti‑invasive properties refer to the ability of compounds to slow down or block cancer cells from moving. In this study, a unique plant-derived lignan‑based library was screened against glioblastoma (brain cancer) cell lines to evaluate anti-invasive and cytotoxicity effects.

## Results and Discussion

**Figure 1:**
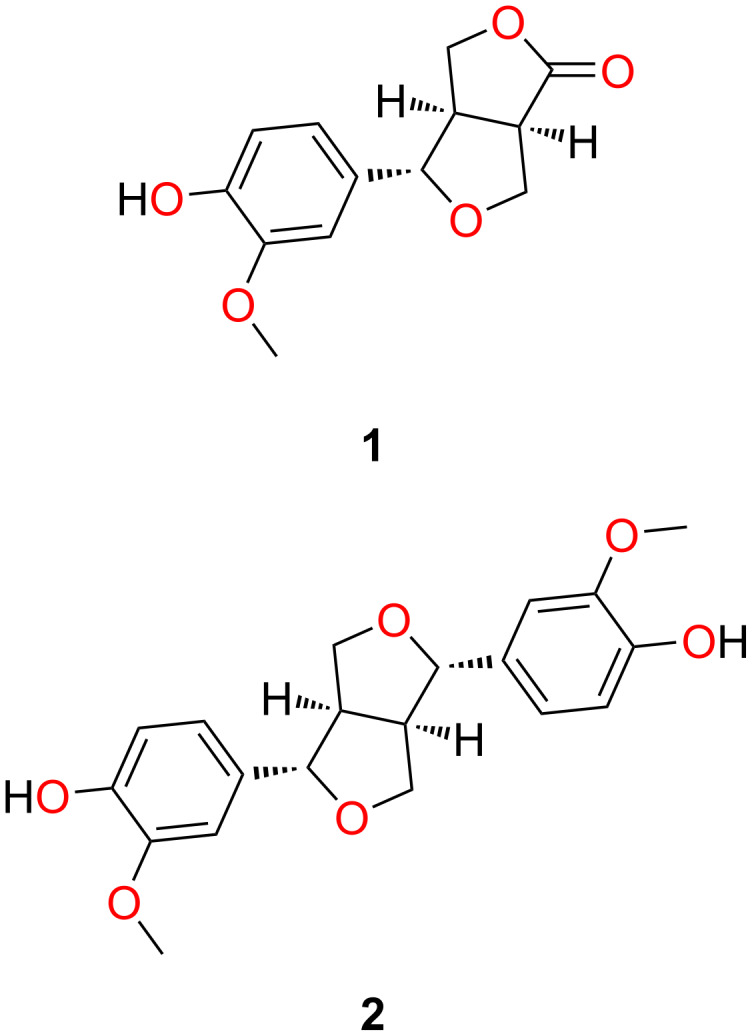
Chemical structures of the two known natural products, (+)-salicifoliol (**1**) and (+)-pinoresinol (**2**), that were obtained from the CH_2_Cl_2_/MeOH extracts of *Eremophila maculata* seeds.

The air-dried and ground seeds of *E. maculata* were sequentially extracted with CH_2_Cl_2_ and MeOH. Subsequent purifications of these two extracts afforded the two known natural products (+)-salicifoliol (**1**) [15–16] and (+)-pinoresinol (**2**, Figure 1) [17]. Their structures were determined by 1D (^1^H and ^13^C) and 2D (COSY, HMBC, HSQC, and ROESY) NMR spectroscopy together with MS data analysis. During our comparison of spectroscopic and chiro-optical data for (+)-salicifoliol (**1**) with reported literature values [16], we noted that an earlier paper [15] reporting the chemical structure of **1** had misdrawn the stereochemistry. The stereochemical assignment for (+)-salicifoliol (**1**) has been corrected as shown in Figure 1.

**Figure 2:**
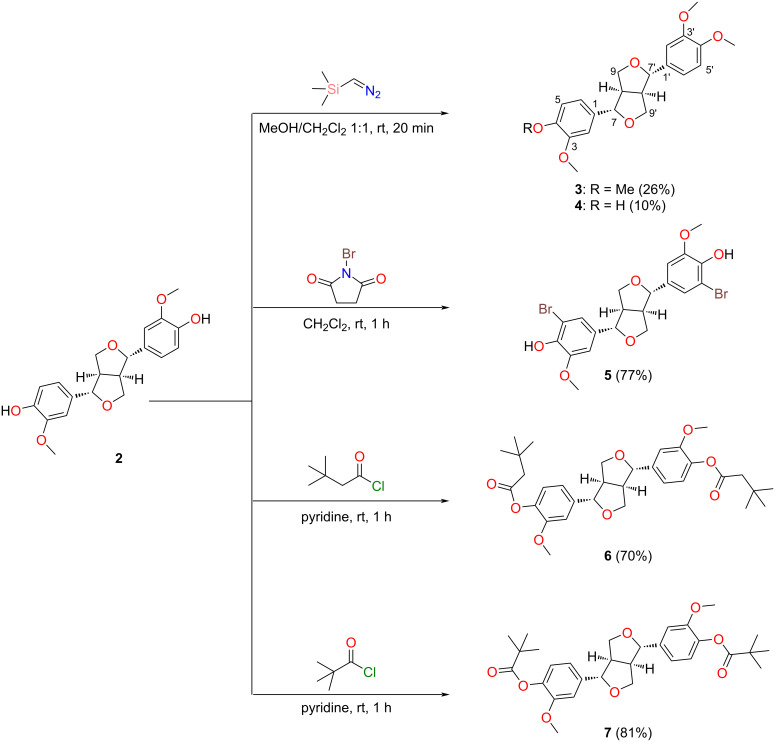
Reaction schemes detailing the semisynthesis of (+)-eudesmin (**3**), (+)-phillygenin (**4**), (+)-5,5'-dibromopinoresinol (**5**), (+)-4,4'-di(3,3-dimethylbutanoyl)pinoresinol (**6**), and (+)-4,4'-dipivaloylpinoresinol (**7**) from (+)-pinoresinol (**2**).

The most abundant metabolite isolated during these chemical studies was (+)-pinoresinol (**2**), which has been reported to exhibit a variety of bioactivities. These include antifungal activity against the human pathogens *Candida albicans* (MIC = 12.5 µM), *Trichosporon beigelii* (MIC = 25 µM), and *Malassezia furfur* (MIC = 12.5 µM) [20]; antioxidant activity with the inhibition of CuSO_4_-induced peroxidation of low-density lipoprotein in a concentration-dependent manner from 0.1−10 µM [21], and anti-invasiveness activity on HT115 human colon cancer cells at a concentration ranging from 1.56−25 µM with a reduction of up to 65% invasion in comparison to the control [22]. Owing to the interesting biological activity of **2** and its moderate abundance in the seeds of *E. maculata*, we decided to generate five semisynthetic analogues **3**−**7** (Figure 2) and evaluate these compounds for in vitro cytotoxicity and anti-invasive properties using two glioblastoma cancer cell lines [23–24].

During our semisynthetic studies, several reactions including methylations, halogenations, and acylations were performed, leading to the generation of five analogues in varying yield (10−81%) and high purity (>95%). Semisynthetic analogues **3**−**5** are known compounds [25–26]. However, some of these compounds have not been fully characterized. Regarding compound **5**, this molecule had previously been reported as an intermediate in the total synthesis of racemic pinoresinol [25]. Through a bromination reaction using *N*-bromosuccinimide (NBS) in CH_2_Cl_2_ at room temperature (1 h), we produced **5** as a single enantiomer (i.e., (+)-5,5'-dibromopinoresinol). Herein, we report the full spectroscopic and spectrometric characterization of this molecule. Furthermore, analogues **6** and **7** are new semisynthetic molecules and were also fully characterized following NMR, UV–vis, [α]_D_, ECD, and ESIMS data analyses.

**Figure 3:**
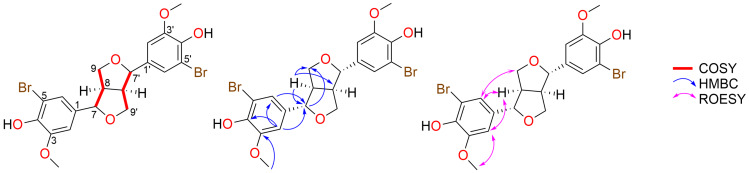
Key COSY, HMBC, and ROESY correlations for (+)-5,5'-dibromopinoresinol (**5**).

An example of our structure elucidation studies is described below, which focuses on the brominated pinoresinol enantiomer (+)-5,5'-dibromopinoresinol (**5**). HRESIMS data revealed an ion at *m*/*z* 536.9519 [M + Na]^+^ that indicated the molecular formula C_20_H_20_Br_2_O_6_. The ^1^H NMR spectrum of **5** in CDCl_3_ (Table 1) indicated that the symmetry of the reaction starting material (+)-pinoresinol (**2**) had been retained in the brominated product. This resulted in the presence of two signals corresponding to aromatic protons (δ_H_ 7.06 (2H), 6.83 (2H)), as well as two methine (δ_H_ 4.75 (2H), 3.11 (2H)), one methylene (δ_H_ 4.27/3.90, 4H), and one methoxy signal (δ_H_ 3.92, 6H)). The ^13^C NMR and HSQC spectra of **5** indicated a total of 10 unique carbon signals, including one methoxy (δ_C_ 56.6, 2C), two methine (δ_C_ 85.4, 53.9, 4C), one methylene (δ_C_ 71.8, 2C), and six aromatic carbon signals (δ_C_ 147.6, 142.9, 133.3, 122.3, 108.4, 108.0, 12C). Comparison of the ^1^H and ^13^C NMR data of **5** and (+)-pinoresinol (**2**) showed a high degree of homology, with the major differences in NMR data associated with a chemical shift of the aryl resonances. Thus, NMR signals for **5** were assigned following a comparison of the chemical shift with the previously reported natural product **2** and detailed analysis of the 2D NMR data (see Table 1). Key COSY, HMBC, and ROESY correlations for **5** are shown below in Figure 3. Collectively, this data confirmed the planar structure assignment for **5**. Moreover, the relative configuration of the semisynthetic derivative **5** was assigned following ROESY data analysis.

**Table 1:** NMR data for (+)-5,5'-dibromopinoresinol (**5**) in CDCl_3_.^a^

position	δ_H_ (multiplicity, *J* in Hz)	δ_C_, type	COSY	HMBC	ROESY

1	—	133.3, C	—	—	—
2	6.83 (d, 1.8)	108.0, CH	6,7	1,3,4,5,6,7	3-OMe,7,8,9a
3	—	147.6, C	—	—	—
3-OMe	3.92 (s)	56.6, CH_3_	—	3	2
4	—	142.9, C	—	—	—
4-OH	—^b^	—	—	—	—
5	—	108.4, C	—	—	—
6	7.06 (dd, 1.8; 0.5)	122.3, CH	2,7	2,3,4,5,7	7,8,9a
7	4.75 (d, 4.2)	85.4, CH	2,6,8	1,2,6,8,9	2,6,8,9b
8	3.11 (m)	53.9, CH	7,9a,9b	1,7,9,7',8',9'	2,6,7,9a
9a	4.27 (m)	71.8, CH_2_	8,9b	1,7,8,7'	2,6,8,9b
9b	3.90 (dd, 9.3; 3.6)		8,9a	7,7'	7
1'	—	133.3, C	—	—	—
2'	6.83 (d, 1.8)	108.0, CH	6',7'	1',3',4',5',6',7'	3'-OMe,7',8',9'a
3'	—	147.6, C	—	—	—
3'-OMe	3.92 (s)	56.6, CH_3_	—	3'	2'
4'	—	142.9, C	—	—	—
4'-OH	—^b^		—	—	—
5'		108.4, C	—	—	—
6'	7.06 (dd, 1.8; 0.5)	122.3, CH	2',7'	2',3',4',5',7'	7',8',9'a
7'	4.75 (d, 4.2)	85.4, CH	2',6',8'	1',2',6',8',9'	2',6',8',9'b
8'	3.11 (m)	53.9, CH	7',9'a,9'b	1',7',9',7,8,9	2',6',7',9'a
9'a	4.27 (m)	71.8, CH_2_	8',9'b	1',7',8',7	2',6',8',9'b
9'b	3.90 (dd, 9.3; 3.6)		8',9'a	7',7	7'

^a^Spectra recorded at 25 °C (800 MHz for ^1^H and 200 MHz for ^13^C). ^b^Signal not observed.

**Figure 4:**
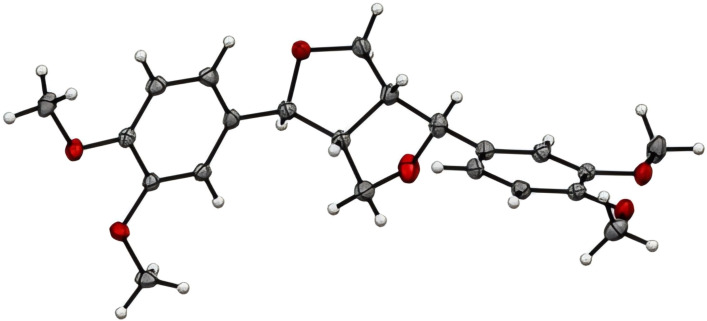
ORTEP drawing of (+)-eudesmin (**3**).

**Figure 5:**
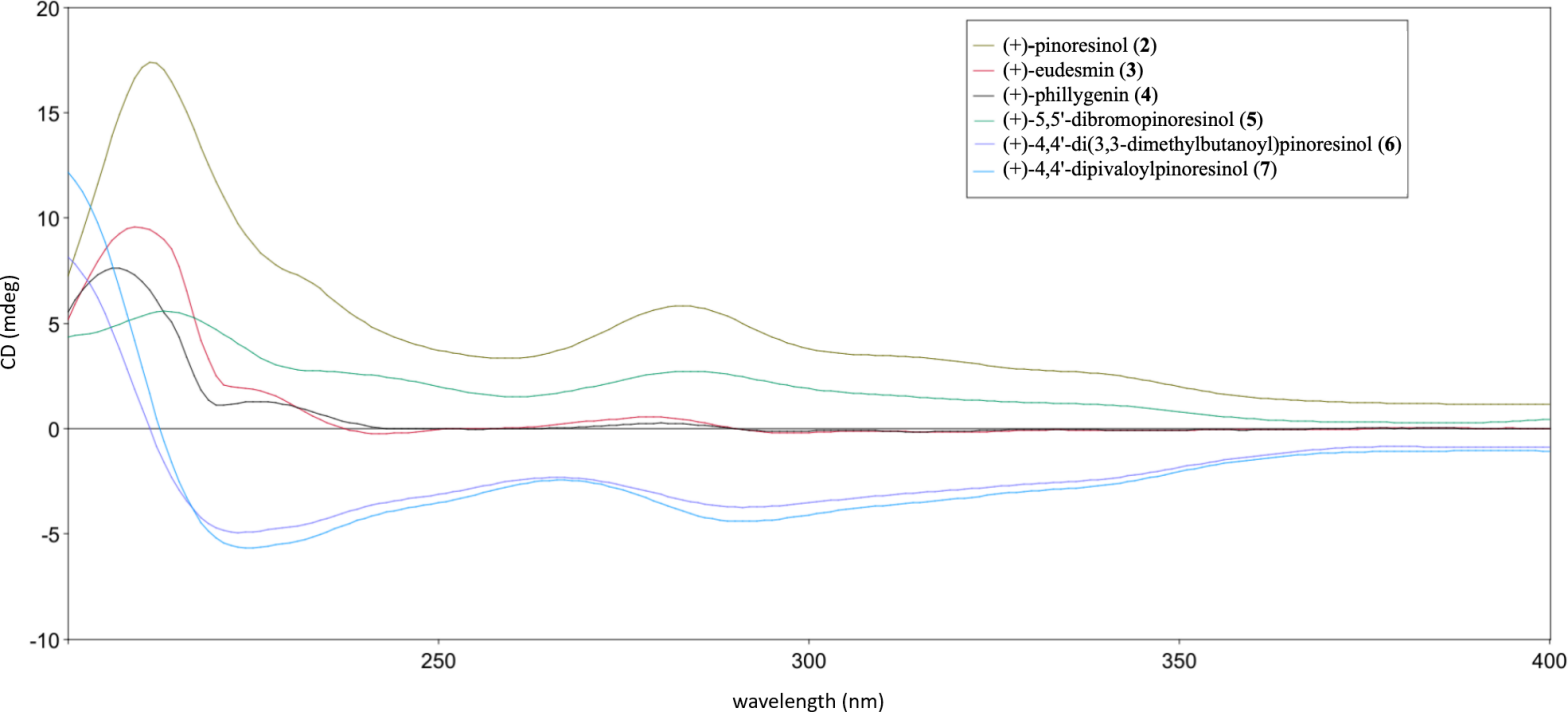
ECD data for compounds **2**–**7** in MeOH.

Following the slow evaporation of a methanolic solution of (+)-eudesmin (**3**), suitable crystals were obtained for X-ray crystallographic studies (Figure 4), which enabled the absolute configuration of **3** to be assigned as 7*S*,8*R*,7'*S*,8'*R*. Moreover, this is the first report of a crystal structure of (+)-eudesmin (**3**). In 2015, Lu et al. reported the purification and full characterization of both enantiomers of eudesmin from *Acorus tatarinowii*, along with the X-ray crystallographic structure (Cu source) for (−)-eudesmin only, which enabled the absolute configuration of this stereoisomer to be assigned [27]. Our experimental data and stereochemical assignment for (+)-eudesmin (**3**) are consistent with those reported by Lu et al. [27]. Furthermore, during our studies, ECD data was recorded for (+)-eudesmin (**3**), (+)-pinoresinol (**2**), and analogues **4**−**7** (Figure 5). As expected, similar ECD data was observed for all compounds, and thus the absolute configuration of all new derivatives reported here was assigned as 7*S*,8*R*,7'*S*,8'*R* [28]. Of note, the ECD data for the two new pinoresinol ester derivatives **6** and **7** showed the biggest differences to the natural products owing to the addition of UV-active acyl moieties.

**Figure 6:**
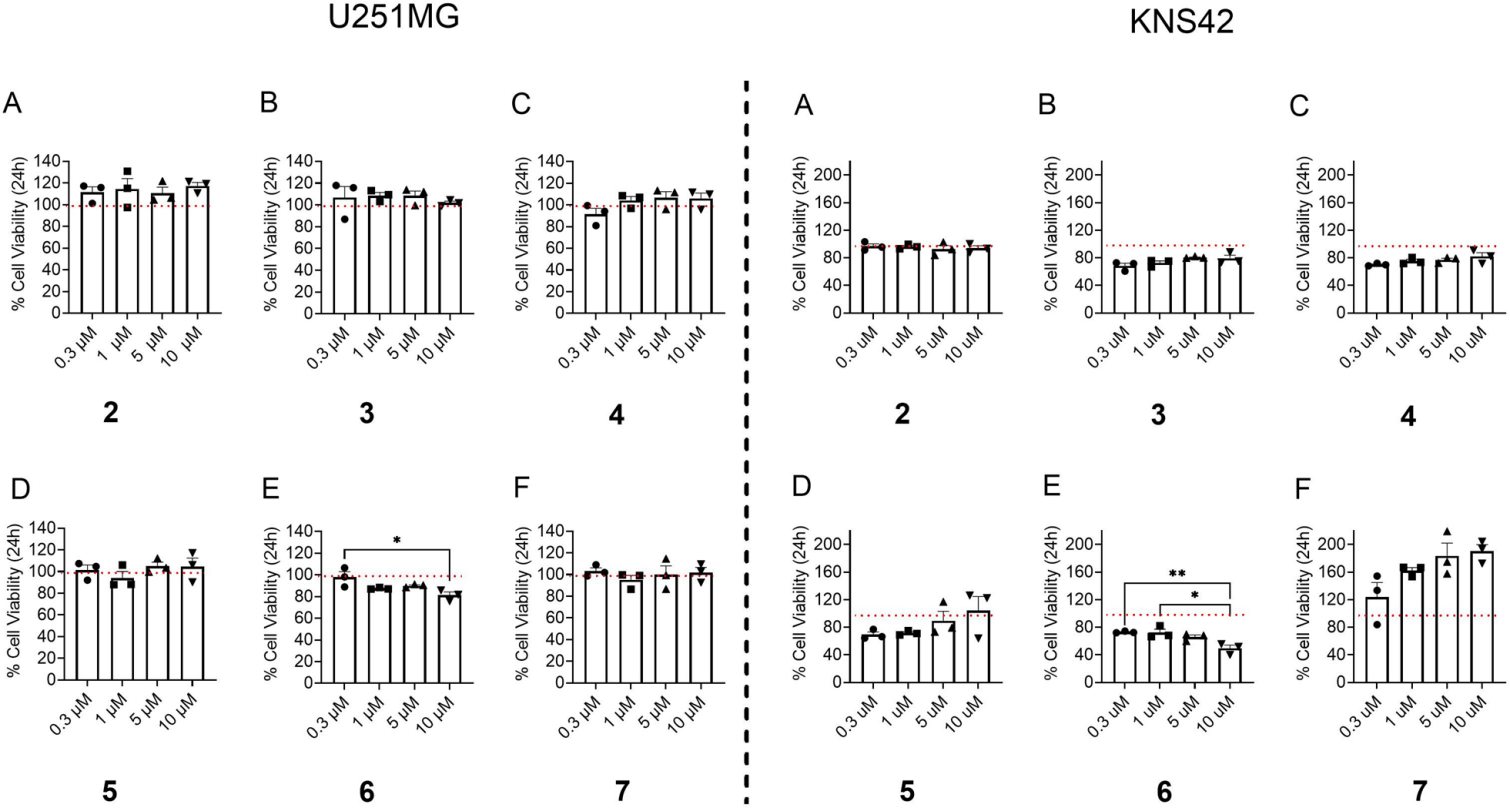
Percentage of cell viability for (+)-pinoresinol (**2**) and its analogues (+)-eudesmin (**3**), (+)-phillygenin (**4**), (+)-5,5'-dibromopinoresinol (**5**), (+)-4,4'-di(3,3-dimethylbutanoyl)pinoresinol (**6**), and (+)-4,4'-dipivaloylpinoresinol (**7**) at different concentrations (0.3, 1, 5, and 10 µM) on cell lines U251MG (left) and KNS42 (right). Cells were seeded in a 96-well plate (Costar #3596) in triplicates at 100 µL/well (i.e., 1,500 cells/well) and incubated for 24 h at 37 °C and 5% CO_2_. Vehicle control (positive control) was the solvent DMSO, in which all compounds were dissolved, plus cells. The final concentration of DMSO was 0.01%. Medium with only cells was the negative control. All data normalized to vehicle control. *n* = 3 replicates.

Owing to our developing interest in identifying natural products and derivatives that are cytotoxic to human cancer cell lines and/or block invasion or migration of proliferating cancer cells [23,29], all compounds were tested against two human glioblastoma cell lines, U251MG and KNS42 [23–24]. Glioblastoma, which is also known as glioblastoma multiforme (GBM), is a grade IV glioma and one of the deadliest human cancers [30]. Following cytotoxicity evaluations of (+)-pinoresinol (**2**) and its analogues **3**–**7** against the U251MG cell line, compound (+)-4,4'-di(3,3-dimethylbutanoyl)pinoresinol (**6**) showed slight cytotoxicity (19% cell viability reduction) at a concentration of 10 µM. Also, on the KNS42 cell line, only (+)-4,4'-di(3,3-dimethylbutanoyl)pinoresinol (**6**) showed a significant reduction (50%) in cell viability at 10 µM (Figure 6). Interestingly, (+)-4,4'-dipivaloylpinoresinol (**7**) seemed to increase cell viability in the KNS42 assay at all tested concentrations. As such, further investigations into these data is warranted (Figure 6).

**Figure 7:**
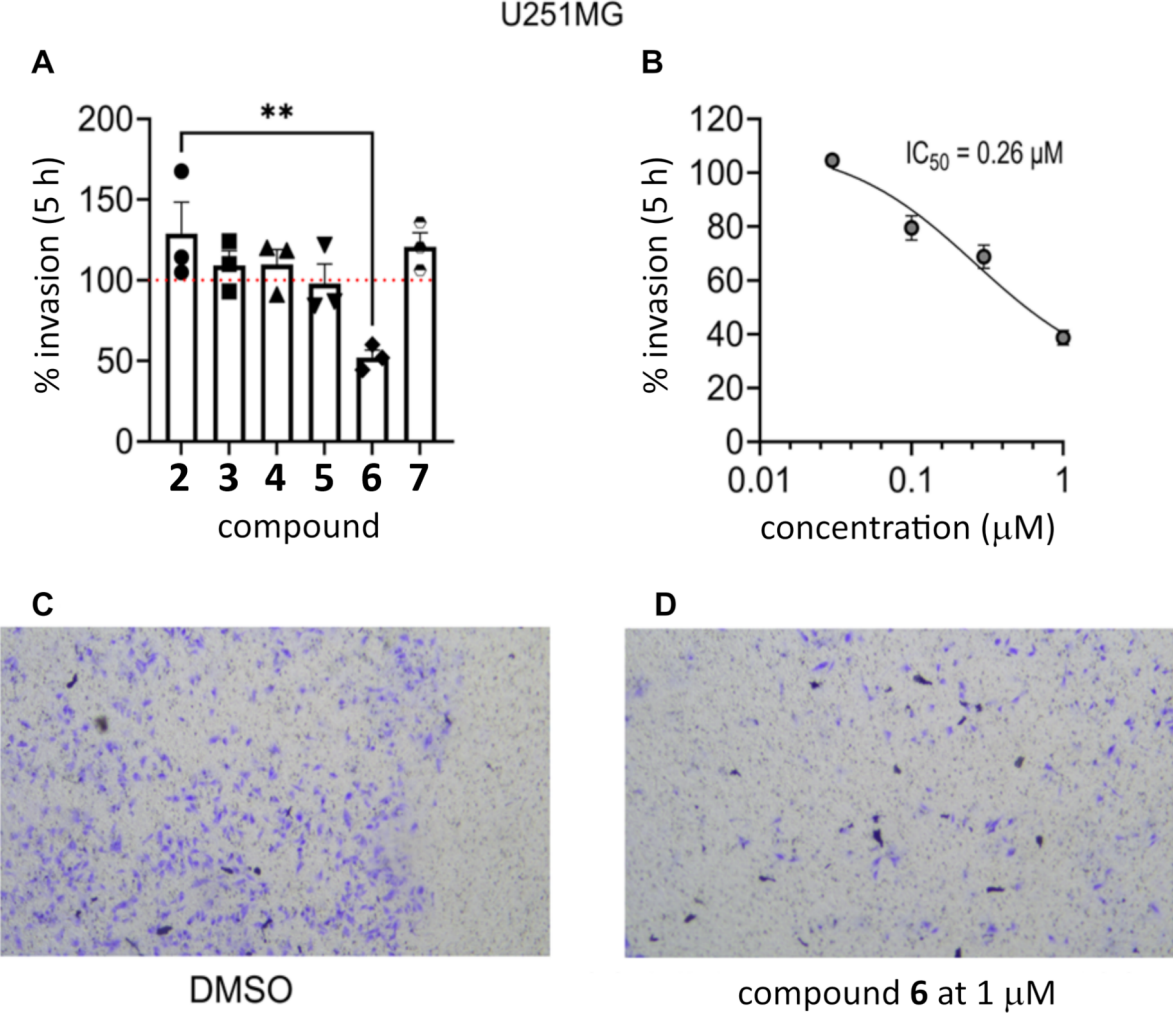
(A) Transwell invasive assay of the (+)-pinoresinol library **2**–**7** on U251MG cells at a concentration of 1 µM with a treatment time of 5 h. 45,000 cells (100 µL volume) were seeded into the top of each transwell insert (#CLS3422; Sigma-Aldrich) in Dulbecco’s modified Eagle medium (DMEM), with 0% fetal bovine serum (FBS), in triplicates. The bottom wells contained 700 µL of DMEM (10% FBS). This was incubated for 24 h at 37 °C with 5% CO_2_. Vehicle control (positive control) was the solvent DMSO, in which all compounds were dissolved, plus cells. The final concentration of DMSO was 0.01%. (B) The U251MG cells were treated with different concentrations of (+)-4,4'-di(3,3-dimethylbutanoyl)pinoresinol (**6**, (0.03, 0.1, 0.3, and 1 µM) over 5 h to monitor the effect on the inhibition of invasion. The % invasion of U251MG cells was used to calculate the half-maximal inhibition concentration (IC_50_) using GraphPad Prism version 9.02. (C) Representative image of transwell for DMSO on U251MG cells taken with a Leica DMi1 10× objective. (D) Representative image of transwell for 1 µM (+)-4,4'-di(3,3-dimethylbutanoyl)pinoresinol (**6**) on U251MG cells taken with a Leica DMi1 10× objective. All data normalized to vehicle control. *n* = 3 replicates.

Based on the glioblastoma cell viability assay results and the previously reported anti-invasive activity of (+)-pinoresinol (**2**) towards HT115 cancer cells [22], the (+)-pinoresinol-based library **2**–**7** was also tested using a transwell invasion assay. A preliminary screen was carried out for the U251MG cells at a concentration of 1 µM with a treatment time of 5 h, and only (+)-4,4'-di(3,3-dimethylbutanoyl)pinoresinol (**6**) reduced the invasion of the cells (by ≈50%) when compared to the vehicle control. None of the other compounds tested reduced the invasive capability of the cells (Figure 7A).

**Figure 8:**
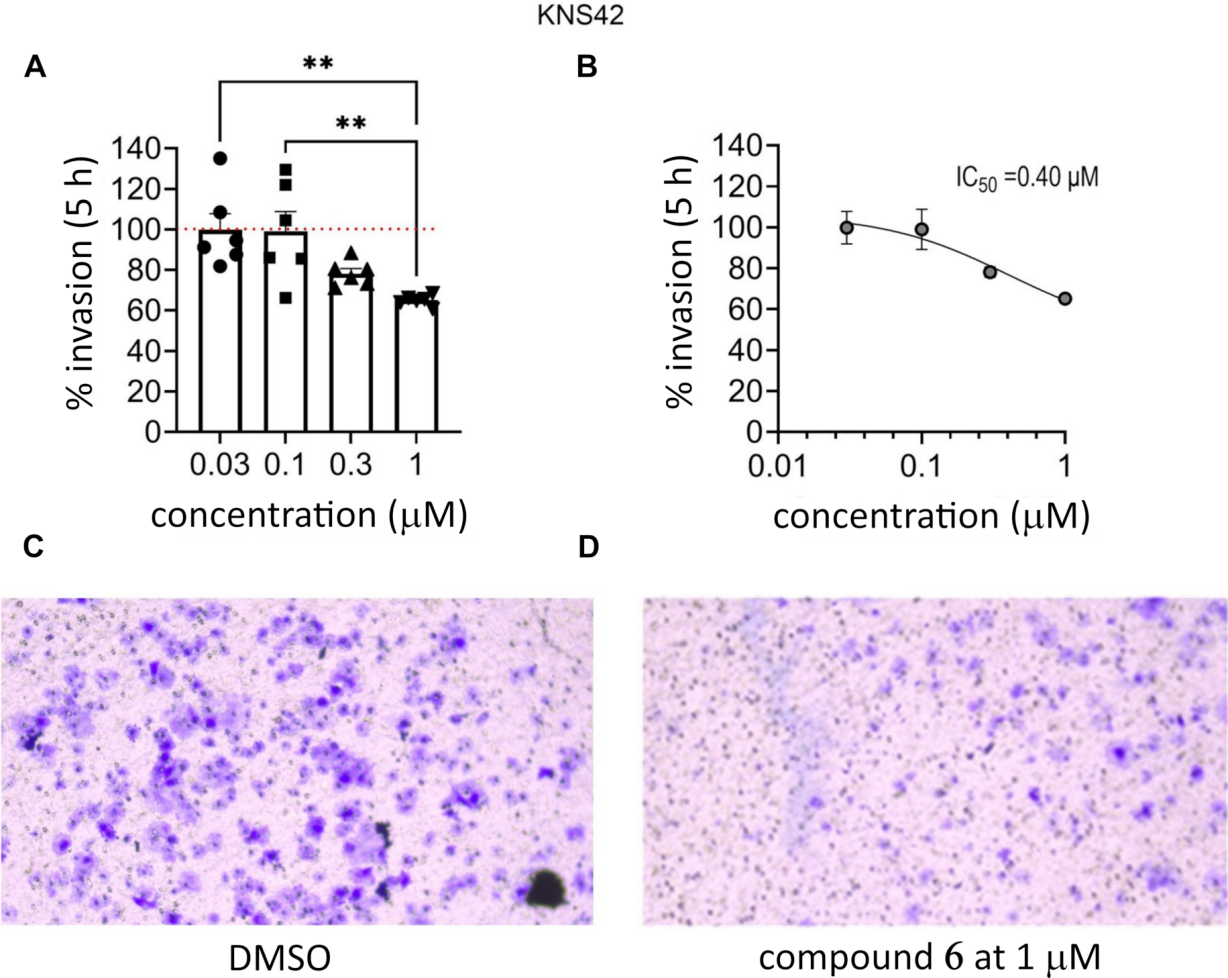
(A) Transwell invasion assay of (+)-4,4'-di(3,3-dimethylbutanoyl)pinoresinol (**6**) on KNS42 cells at a concentration range of 0.03–1 µM with a treatment time of 5 h. 45,000 cells (100 µL volume) were seeded into the top of each transwell insert (#CLS3422; Sigma-Aldrich) in DMEM (0% FBS) in triplicates. The bottom wells contained 700 µL of DMEM (10% FBS). This was incubated for 24 h at 37 °C with 5% CO_2_. Vehicle control (positive control) was the solvent DMSO, in which all compounds were dissolved, plus cells. The final concentration of DMSO was 0.01%. (B) The % invasion of KNS42 cells was used to calculate the half-maximal inhibition concentration (IC_50_) using GraphPad Prism version 9.02. (C) Representative image of transwell for DMSO on KNS42 cells taken with a Leica DMi1 10× objective. (D) Representative image of transwell for 1 µM (+)-4,4'-di(3,3-dimethylbutanoyl)pinoresinol (**6**) on KNS42 cells taken with a Leica DMi1 10× objective. All data normalized to vehicle control. *n* = 3 replicates.

Based on the results of the transwell invasion assay (Figure 7), (+)-4,4'-di(3,3-dimethylbutanoyl)pinoresinol (**6**) was selected for further study. The U251MG cells were treated with different concentrations of **6** (0.03, 0.1, 0.3, and 1 µM) over 5 h and assayed for inhibition of invasion. The IC_50_ for compound **6** was calculated as 0.26 µM (Figure 7B). The KNS42 cells were treated with different concentrations of (+)-4,4'-di(3,3-dimethylbutanoyl)pinoresinol (**6**, 0.03, 0.1, 0.3, and 1 µM) for 5 h and assayed for inhibition of invasion (Figure 8A). The compound inhibited invasion at 0.3 and 1 µM by up to 30%, and the IC_50_ for compound **6** was calculated as 0.40 µM (Figure 8).

## Conclusion

In summary, the first chemical investigations of the seeds of *E. maculata* yielded the known metabolites (+)-salicifoliol (**1**, 0.014 dry wt %) and (+)-pinoresinol (**2**, 0.134 dry wt %). The most abundant metabolite **2** was chosen for semisynthetic studies. Five analogues were successfully generated using standard medicinal chemistry methodologies, and all derivatives were fully characterized. The (+)-pinoresinol library **2**–**7** was evaluated on glioblastoma-derived U251MG and KNS42 cell lines for their cytotoxicity. Only (+)-4,4'-di(3,3-dimethylbutanoyl)pinoresinol (**6**) showed some cytotoxicity. Transwell invasion assays on the library against U251MG cells showed that analogue **6** was the most active compound, as it reduced the invasion of the cells by ≈50%, with an IC_50_ value of 0.26 µM. Consequently, compound **6** was further tested in the pediatric cell line KNS42 and was shown to reduce cell invasion in the transwell assay, with an IC_50_ value of 0.40 µM. These findings provide a rationale for more detailed anticancer studies on pinoresinol and its derivatives in the future.

## Experimental

### General experimental procedures

Melting points were measured using a Cole-Parmer melting point apparatus and are uncorrected. Specific rotations were determined on a JASCO P-2000 polarimeter. UV–vis spectra were recorded using a CAMSPEC M501 UV–vis spectrophotometer. ECD spectra were obtained on a JASCO J-1500 spectropolarimeter and processed using the software SDAR [31]. NMR spectra were recorded at 25 °C on a Bruker AVANCE III HD 500 or HDX 800 MHz NMR spectrometer equipped with a cryoprobe. The ^1^H and ^13^C chemical shifts were referenced to solvent peaks for CDCl_3_ at δ_H_ 7.26 and δ_C_ 77.2, respectively. UHPLC–LRESIMS analysis was performed using a previously reported method [32], and data was recorded on an Ultimate 3000 RS UHPLC coupled to a Thermo Fisher Scientific MSQ Plus single quadruple ESI mass spectrometer. HRESIMS data were acquired on Bruker maXis II ETD ESI-qTOF. GRACE C_18_- or C_8_-bonded silica (35–70 µm, 60 Å) was used for preadsorption work before HPLC separations, and the preadsorbed sample was packed into an Alltech stainless steel guard cartridge (10 × 30 mm). GRACE C_8_-bonded silica (35–70 µm, 60 Å) was used for the C_8_ flash column. Isolute Si SPE cartridge (53 µm, 20 g) was used for the separations. A Thermo Fisher Scientific Dionex Ultimate 3000 UHPLC was used for semipreparative HPLC separations. A Thermo BetaSil C_18_ column (5 μm, 100 Å, 150 × 21.2 mm), Luna C_18_ HPLC column (5 μm, 100 Å, 150 × 21.2 mm), or a BetaSil phenyl HPLC column (5 μm, 100 Å, 250 × 21.2 mm) was used for semipreparative HPLC separation. Merck silica gel 60 F_254_ precoated aluminium plates were used for TLC and analyzed under UV light at 254 and 365 nm. For large-scale studies, the plant material was extracted at room temperature using an Edwards Instrument Company Bio-line orbital shaker set to 200 rpm. All chemical reagents used throughout the experiments were purchased from Sigma-Aldrich. All solvents used for chromatography, UV–vis, ECD, [α]_D_, and MS were Honeywell Burdick & Jackson brand or Lab-Scan HPLC grade. H_2_O was filtered using a Sartorius Stedium Arium Pro VF ultrapure H_2_O system.

### Plant material

*E. maculata* seeds used in these studies were obtained from the NatureBank biota library housed at the Institute for Biomedicine and Glycomics, Griffith University, Australia. This seed sample was collected on December 3, 1993 at Mount Crosby, Queensland, Australia. A voucher specimen (AQ600324) has been deposited with the Queensland Herbarium.

### Extraction and isolation

The air-dried and ground seeds of *E*. *maculata* (10 g) were extracted with CH_2_Cl_2_ (250 mL, 2 h; 250 mL, 16 h) to afford 120.5 mg of crude CH_2_Cl_2_ extract. Subsequent extraction with MeOH (250 mL, 2 h; 250 mL, 16 h) afforded 1.43 g of crude MeOH extract. The CH_2_Cl_2_ extract (120.5 mg) was subjected to chromatography using a silica flash column (3 × 6 cm) and a 10% stepwise gradient from 100% *n*–hexane to 100% EtOAc (100 mL washes) followed by a 10% MeOH/90% CH_2_Cl_2_ flush (100 mL) to afford 100 fractions that were analyzed by TLC and UHPLC–MS. These data enabled the combining (or discarding) of several fractions, which resulted in 12 final fractions (FA−FL). All 12 samples were analyzed by UHPLC–MS and ^1^H NMR spectroscopy. Fraction H (22.2 mg) was preadsorbed to C_18_-bonded silica (≈1 g), packed into a guard cartridge, and attached to a semipreparative Luna C_18_ HPLC column. A linear gradient from 40% MeOH (0.1% TFA)/60% H_2_O (0.1% TFA) to 100% MeOH (0.1% TFA) at a flowrate of 4 mL/min over 60 min afforded salicifoliol (**1**, 1.4 mg, *t*_R_ 11 min, 0.014 dry wt %) and (+)-pinoresinol (**2**, 13.4 mg, *t*_R_ 21–22 min, 0.134 dry wt %). The MeOH extract (1.3 g) was divided into three equal portions and individually preadsorbed to phenyl-bonded silica (≈1 g), packed into a guard cartridge, and subjected to three separate semipreparative BetaSil phenyl HPLC purifications. A linear gradient from 10% MeOH (0.1% TFA)/90% H_2_O (0.1% TFA) to 100% MeOH (0.1% TFA) at a flowrate of 9 mL/min over 60 min produced semipure (+)-pinoresinol (**2**, 113.5 mg, *t*_R_ 41−42 min), which was preadsorbed to C_18_-bonded silica (≈1 g), packed into a guard cartridge, and subjected to semipreparative C_18_ HPLC purification. A linear gradient from 40% MeOH (0.1% TFA)/60% H_2_O (0.1% TFA) to 100% MeOH (0.1% TFA) over 50 min, followed by isocratic conditions of 100% MeOH (0.1% TFA) for 10 min, all at a flow rate of 9 mL/min, gave pure (+)-pinoresinol (**2**, 19.5 mg, *t*_R_ 13 min, 0.195 dry wt %). The extraction and isolation protocol described above was repeated on an additional 10 g of seeds to provide a sufficient quantity of **2** for the semisynthetic studies detailed below. The previously described plant compounds salicifoliol (**1**) [15–16] and (+)-pinoresinol (**2**) [17] were identified following 1D and 2D NMR (^1^H, ^13^C, COSY, HSQC, HMBC, and ROESY), [*α*]_D_, MS data analysis, and comparison with literature values.

(+)-Salicifoliol (**1**): clear gum; [α]_D_^25^ +36.4 (*c* 0.09, MeOH); lit. [α]_D_^25^ +56.3 (*c* 0.06, MeOH) [33]; see Supporting Information File 1 for ^1^H and ^13^C NMR data in CDCl_3_; LRESIMS *m*/*z*: [M + H]^+^ 251, [M − H]^−^ 249.

(+)-Pinoresinol (**2**): brown gum; [α]_D_^25^ +55.5 (*c* 0.07, MeOH); lit. [α]_D_^25^ +20.0 (*c* 0.75, MeOH) [34]; UV–vis (MeOH) λ_max_ (log ε) 230 (3.94), 279 (3.59) nm; ECD (MeOH) λ_ext_ (Δε) 208 (+6.25), 256 (−0.08), 282 (+0.26) nm; see Supporting Information File 1 for ^1^H and ^13^C NMR data in CDCl_3_; LRESIMS *m*/*z*: [M + Na]^+^ 381, [M − H]^−^ 357; HRESIMS *m*/*z*: [M + Na]^+^ calcd for C_20_H_22_NaO_6_, 381.1308; found, 381.1307.

### Preparation and purification of semisynthetic (+)-pinoresinol derivatives **3**–**7**

#### Methylation of (+)-pinoresinol (**2**)

(+)-Pinoresinol (**2**, 15.8 mg, 0.044 mmol) was dissolved in MeOH/CH_2_Cl_2_ 1:1 (250 μL) before (trimethylsilyl)diazomethane (2.0 M in diethyl ether, 150 μL) was added dropwise. The reaction mixture was stirred for 20 min at room temperature and then transferred to a separatory funnel containing CH_2_Cl_2_ (3 mL) and H_2_O (3 mL). The H_2_O layer was washed with CH_2_Cl_2_ (3 × 3 mL), and the CH_2_Cl_2_-soluble material was dried, preadsorbed to C_18_-bonded silica (≈1 g), packed into a guard cartridge, and subjected to semipreparative HPLC using a BetaSil C_18_-bonded silica column. Isocratic conditions of 10% MeOH (0.1% TFA)/90% H_2_O (0.1% TFA) were applied for 10 min, followed by a linear gradient to 40% MeOH (0.1% TFA)/60% H_2_O (0.1% TFA) over 5 min, a linear gradient to 100% MeOH (0.1% TFA) over 35 min, and then isocratic conditions of 100% MeOH (0.1% TFA) for 10 min, all at a flow rate of 9 mL/min, to give the dimethylated product (+)-eudesmin (**3**, 4.4 mg, *t*_R_ 35−36 min, 26% yield) and the monomethylated product (+)-phillygenin (**4**, 1.7 mg, *t*_R_ 32−33 min, 10% yield).

(+)-Eudesmin (**3**): clear needles (4.4 mg, 26%); mp 94−96 °C (lit. [35] mp 98−100 °C); [α]_D_^24^ +110.0 (*c* 0.4, CHCl_3_); lit. [α]_D_ +61.0 (*c* 0.4, CHCl_3_) [35]; UV–vis (MeOH) λ_max_ (log ε) 230 (4.39), 278 (3.97) nm; ECD (MeOH) λ_ext_ (Δε) 209 (+9.57), 280 (+0.53) nm; see Supporting Information File 1 for ^1^H (800 MHz) and ^13^C NMR (200 MHz) data in CDCl_3_; LRESIMS *m*/*z*: [M + Na]^+^ 409, [2M + Na]^+^ 795.

(+)-Phillygenin (**4**): clear gum (1.7 mg, 10%); [α]_D_^24^ +46.8 (*c* 0.6, CHCl_3_); lit. [α]_D_ +91.6 (*c* 0.5, CHCl_3_) [35]; UV–vis (MeOH) λ_max_ (log ε) 230 (4.01), 279 (3.61) nm; ECD (MeOH) λ_ext_ (Δε) 206 (+6.69), 280 (+0.22) nm; see Supporting Information File 1 for ^1^H (800 MHz) and ^13^C NMR (200 MHz) data in CDCl_3_; LRESIMS *m*/*z*: [M + Na]^+^ 395.

#### Bromination of (+)-pinoresinol (**2**)

(+)-Pinoresinol (**2**, 10.0 mg, 0.028 mmol) was dissolved in CH_2_Cl_2_ (600 μL) before NBS (9.9 mg, 0.056 mmol, 2 equiv) was added. The reaction mixture was stirred for 1 h at room temperature and dried before being preadsorbed to C_18_-bonded silica (≈1 g). Then, it was packed into a guard cartridge and subjected to semipreparative HPLC using a BetaSil C_18_-bonded silica column. Isocratic conditions of 10% MeOH (0.1% TFA)/90% H_2_O (0.1% TFA) were maintained for 10 min, followed by a linear gradient to 40% MeOH (0.1% TFA)/60% H_2_O (0.1% TFA) over 5 min, a linear gradient to 100% MeOH (0.1% TFA) over 35 min, and then isocratic conditions of 100% MeOH (0.1% TFA) for 10 min, all at a flow rate of 9 mL/min, to give (+)-5,5'-dibromopinoresinol (**5**, 11.0 mg, *t*_R_ 37−38 min, 77% yield).

(+)-5,5'-Dibromopinoresinol (**5**): clear gum (11.0 mg, 77%); [α]_D_^25^ +154.7 (*c* 0.7, MeOH); UV–vis (MeOH) λ_max_ (log ε) 220 (4.28), 286 (3.61) nm; ECD (MeOH) λ_ext_ (Δε) 213 (+2.61), 284 (+1.27), 340 (0.52) nm; see Supporting Information File 1 for ^1^H (800 MHz) and ^13^C NMR (200 MHz) data in CDCl_3_; LRESIMS *m*/*z*: [M (^79^Br_2_) − H]^−^ 513, [M (^79^Br^81^Br) − H]^−^ 515, [M (^81^Br_2_) − H]^−^ 517; HRESIMS *m*/*z*: [M + Na]^+^ calcd for C_20_H_20_^79^Br_2_NaO_6_, 536.9519; found, 536.9519 [25].

#### Acylation of (+)-pinoresinol (**2**)

(+)-Pinoresinol (**2**, 13.4 mg, 0.037 mmol) was dissolved in pyridine-*d*_5_ (200 μL) before 3,3-dimethylbutyryl chloride (150 μL) was added dropwise. The reaction mixture was stirred for 1 h at room temperature and dried before being preadsorbed to C_18_-bonded silica (≈1 g). This was then packed into a guard cartridge and subjected to semipreparative HPLC using a BetaSil C_18_-bonded silica column. Isocratic conditions of 10% MeOH (0.1% TFA)/90% H_2_O (0.1% TFA) were maintained for 10 min, followed by a linear gradient to 40% MeOH (0.1% TFA)/60% H_2_O (0.1% TFA) over 5 min, a linear gradient to 100% MeOH (0.1% TFA) over 35 min, and then isocratic conditions of 100% MeOH (0.1% TFA) for 10 min, all at a flow rate of 9 mL/min, to give pure (+)-4,4'-di(3,3-dimethylbutanoyl)pinoresinol (**6**, 14.6 mg, *t*_R_ 48−50 min, 70% yield). (+)-Pinoresinol (**2**, 8.0 mg, 0.022 mmol) was dissolved in pyridine-*d*_5_ (200 μL) before pivaloyl chloride (100 μL) was added dropwise. The reaction mixture was stirred for 1 h at room temperature and purified using the same method described above to give (+)-4,4'-dipivaloylpinoresinol (**7**, 9.5 mg, *t*_R_ 46−50 min, 81% yield).

(+)-4,4'-Di(3,3-dimethylbutanoyl)pinoresinol (**6**): clear gum (14.6 mg, 70%); [α]_D_^25^ +96.2 (*c* 0.9, MeOH); UV–vis (MeOH) λ_max_ (log ε) 220 (4.27), 272 (3.78) nm; ECD (MeOH) λ_ext_ (Δε) 223 (−2.49), 292 (−1.88) nm; see Supporting Information File 1 for ^1^H (800 MHz) and ^13^C NMR (200 MHz) data in CDCl_3_; LRESIMS *m*/*z*: [M − H]^−^ 553, [M + Na]^+^ 577; HRESIMS *m*/*z*: [M + Na]^+^ calcd for C_32_H_42_NaO_8_, 577.2772; found, 577.2766.

(+)-4,4'-Dipivaloylpinoresinol (**7**): clear gum (9.5 mg, 81%); [α]_D_^25^ +30.0 (*c* 0.05, MeOH); UV–vis (MeOH) λ_max_ (log ε) 220 (4.27), 273 (3.76) nm; ECD (MeOH) λ_ext_ (Δε) 224 (2.71), 291 (−2.11); see Supporting Information File 1 for ^1^H (800 MHz) and ^13^C NMR (200 MHz) data in CDCl_3_; LRESIMS *m*/*z*: [M + Na]^+^ 549; HRESIMS *m*/*z*: [M + Na]^+^ calcd for C_30_H_38_NaO_8_, 549.2459; found, 549.2458.

#### X-ray crystallography analysis of (+)-eudesmin (**3**)

Intensity data were collected on a Rigaku XtaLAB synergy diffractometer using Cu Kα radiation at 100.0(1) K using an Oxford Cryostream cooling device. The structure was solved by direct methods and difference Fourier synthesis [36]. Hydrogen atoms bound to the carbon atom were placed at their idealized positions and included in subsequent refinement cycles. Thermal ellipsoid plots were generated using the program Mercury [37], integrated within the WINGX suite of programs [38]. Crystallographic data for (+)-eudesmin (**3**) have been deposited with the Cambridge Crystallographic Data Centre and assigned CCDC Number 2366804. These data can be obtained free of charge from the Cambridge Crystallographic Data Centre via http://www.ccdc.cam.ac.uk/data_request/cif.

#### Crystal data for (+)-eudesmin (**3**)

C_22_H_26_O_6_, *M* = 386.43, *T* = 100.0 K, 1.54184 Å, monoclinic, space group *P*2_1_, *a* = 11.42420(10) Å, *b* = 32.78820(10) Å, *c* = 11.53130(10) Å, β = 116.7680(10)°, *V* = 3856.50(6) Å^3^, *Z* = 8, *Z*’ = 4, *D_c_* = 1.331 mg⋅M^−3^, μ(Cu Kα) = 0.792 mm^−1^, *F*(000) = 1648, crystal size 0.6 × 0.4 × 0.24 mm^3^, 136064 reflections measured, θ_max_ = 79.97°, 16547 independent reflections (*R*_int_ = 0.056), final *R* = 0.0334 (*I* > 2σ(*I*), 16091 data), *wR*(*F*_2_) = 0.0886 (all data), GOF = 1.041, absolute structure parameter = 0.02(3).

### Cancer cell lines and growth

**Figure 9:**
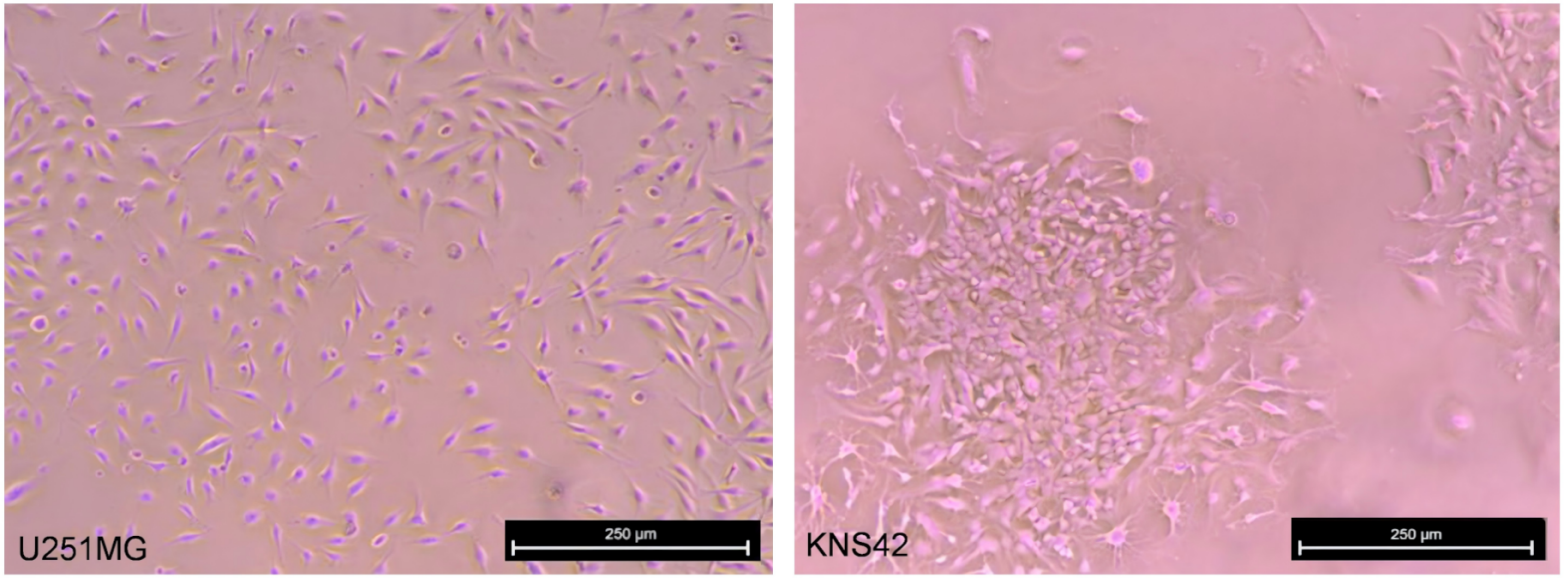
The U251 cell line was derived from a grade III–IV malignant tumor, specifically an astrocytoma, obtained from a 75-year-old male patient. The cell line exhibits a fibroblastic pattern of growth and has a doubling time of 23–24 h. KNS42 cells are derived from a 16-year-old male patient with GBM, and cells appear as polygonal and flat clusters, with a doubling time of approximately 14 h.

Two glioblastoma cell lines U251MG (#203170) and KNS42 (#IF050356) were obtained from Cell Bank Australia and maintained in the laboratory in either DMEM with 10% FBS and 100 U/mL penicillin-streptomycin (Pen-Strep) (Figure 9). Cells were grown to 80% confluence in T75 flasks with appropriate medium. The cells were then detached form the surface of the flask by incubating in 0.5% trypsin-EDTA for 3 min, followed by neutralization with 7 mL of DMEM complete medium.

### Cytotoxicity or percentage of cell viability

An aliquot (10 µL) of the harvested cells was counted in a haemocytometer after the addition of 10 µL of trypan blue to determine the cell count. The remaining cells were centrifuged at 1,100 rpm for 5 min. Based on the cell count, the spun cells were resuspended in the appropriate volume of complete medium to achieve a cell number of 15,000 cells per mL. Cells were seeded in a 96-well plate (Costar #3596) in triplicates at 100 µL/well (i.e., 1,500 cells/well) and incubated for 24 h at 37 °C and 5% CO_2_. Vehicle control (positive control) was the solvent DMSO, in which all compounds were dissolved, plus cells. The final concentration of DMSO was 0.01%. Medium with only cells was the negative control. After incubation, the medium was removed, and 100 µL/well of fresh medium with four different concentrations (0.3, 1, 5, and 10 µM) of the compounds were added. Plates were placed in the incubator for 24 h at 37 °C with 5% CO_2_. After 24 h, the medium was replaced with 100 µL/well of fresh medium with alamarBlue (1:10 dilution, Thermo Fisher #DAL1100) and incubated at 37 °C and 5% CO_2_. AlamarBlue is a redox indicator used for measuring cell viability [39], and the reaction is based on the live cells converting resazurin to resorufin, i.e., blue to pink /red in color [40].

Changes in cell viability were detected using fluorescence (excitation at 530–560 and emission at 590 nm) or absorbance (detected at 570 and 600 nm) in a plate reader (FLUOstar Omega, BMG Labtech). In this study, cell viability was quantitated by measuring changes in fluorescence after 45 and 90 min of incubation at 37 °C and 5% CO_2_. The % cell viability was calculated using the following formula:


[1] $$\begin{array}{l} \%\text{ cell viability}=\\ \frac{\text{fluorescence of treated cells}}{\text{fluorescence of untreated (vehicle-treated) cells}}*100 \end{array}$$

### Transwell invasion assay

This assay is used to study the invasive capabilities of tumor cells to pharmacological inhibitors. It involves the addition of an extracellular matrix, such as Matrigel, on top of a porous membrane that allows cells to invade in response to the chemotactic gradient. The transwell assay protocol was adapted from the method outlined by Justus et al. [41]. Corning 6.5 mm transwell assay plates (24 wells) with polycarbonate inserts (24) containing 8.0 µm pores (Sigma-Aldrich, #CLS3422) were used in this study.

### Cell starvation

U251MG and KNS42 cells were grown in DMEM complete medium in a T75 flask to 50–60% confluency. Cells were washed once with 1× phosphate-buffered saline (PBS), and PBS was replaced with 15 mL of DMEM containing 2% FBS and 100 U/mL Pen-Strep. The flask was placed at 37 °C and 5% CO_2_ for 24 h of starvation.

### Insert preparation

40 µL of Matrigel (1:40 dilution) was added to each insert, followed by fixing for 5 min under UV light in a biosafety cabinet. The inserts were left to dry overnight in the biosafety cabinet. After 24 h, 50 µL of FBS-free DMEM with 100 U/mL Pen-Strep was added to each insert and placed in the incubator at 37 °C and 5% CO_2_ to rehydrate for 1 h.

### Cell preparation

Medium from the starved cells was aspirated, and the cells were washed with 10 mL of 1× PBS. The PBS was replaced with 3 mL of warm 1× trypsin-EDTA solution, and the flask was placed in an incubator at 37 °C and 5% CO_2_ to detach the cells. The trypsin-EDTA medium was neutralized with 7 mL of DMEM complete medium, and the cell suspension was transferred to a 15 mL centrifuge tube. An aliquot (10 µL) was taken to count the number of viable cells after the addition of an equal volume of trypan blue. Depending on the cell count, the cell pellet in the tube was resuspended in a volume of FBS-free medium to give 450,000 cells/mL, such that each insert would contain 45,000 cells/100 µL.

### Assay setup

An aliquot of 700 µL each of DMEM complete medium containing 10% FBS and Pen-Strep and the appropriate test compound (1 µM) was added to the wells of the plate. Positive control was the vehicle control, i.e., cell suspension with DMSO. To each prepared insert, 100 µL of cell suspension (prepared above) with the appropriate test compound was added, and the inserts were placed into the wells with corresponding medium plus compound. The plates were incubated for 5 h in an incubator at 37 °C and 5% CO_2_.

### Cell fixing and staining

After 5 h of incubation, 700 µL of 80% ethanol was added to the empty wells of the plate. The inserts were gently lifted, and a cotton-tipped applicator was used to remove the medium and uninvaded cells from the top of the inserts before being placed into ethanol to fix the cells (7−10 min). The inserts were lifted, any excess ethanol was removed with a cotton-tipped applicator, and the inserts were placed upside down to dry. Ethanol was removed from the wells, and 700 µL of 0.2% w/v crystal violet solution was added to each well. The dried inserts were placed into this solution for 10 min to stain the cells. After 10 min, the inserts were gently lifted and rinsed in autoclaved water 2−3 times to remove excess stain. The inside of the inserts was cleaned with cotton-tipped applicators to remove excess water and stain, and the inserts were placed upside down to dry overnight in a biosafety cabinet.

### Cell imaging and counting

A Leica DMi1 microscope camera with a 10× objective was used to take three images per insert (different fields of view) using the Leica Application Suite (version 3.7.2.22383). Each image was processed in Fiji (ImageJ). A grid was applied to each image by selecting ‘Analyze’>’Tools’>’Grid’, and the cells were counted, grid by grid, using a manual cell counter. The cell counts from the three images per insert were then averaged to obtain the average number of invaded cells per insert.

### Data analysis

All raw data collected from cell counting was processed in Microsoft Excel. This included the average cell number for each replicate and test compound and the average invasion percentage for each sample extract. The % invasion was calculated using the following formula:


[2] $$\%\text{ invasion}=\frac{\text{average cell count for a treatment}}{\text{average cell count of vehicle control}}*100$$

The data was exported into Prism (GraphPad 9.02), where a one-way analysis of variance (ANOVA) was performed to determine whether the results were significant.

### Dose-response curve

The compound that showed significant inhibition of invasion of the cells was selected for this assay. The test compound was diluted to provide a concentration range (0.03, 0.1, 0.3, and 1 µM). These dilutions were used in the assay as detailed in the sections above.

## Supporting Information

File 1 Compound characterization data.

## Data Availability

All data that supports the findings of this study is available in the published article and/or the supporting information of this article.

## References

[1] Villa F A, Gerwick L (2010). Immunopharmacol Immunotoxicol.

[2] Balunas M J, Kinghorn A D (2005). Life Sci.

[3] Achan J, Talisuna A O, Erhart A, Yeka A, Tibenderana J K, Baliraine F N, Rosenthal P J, D'Alessandro U (2011). Malar J.

[4] Kardos N, Demain A L (2011). Appl Microbiol Biotechnol.

[5] Newman D J, Cragg G M (2020). J Nat Prod.

[6] Quinn R J (1999). Drug Dev Res.

[7] Chinnock R J (2007). Eremophila and allied genera: a monograph of the plant family Myoporaceae.

[8] Richmond G S (1993). J Adel Bot Gard.

[9] Sadgrove N J, Jones G L (2013). J Ethnopharmacol.

[10] Barnes E C, Kavanagh A M, Ramu S, Blaskovich M A, Cooper M A, Davis R A (2013). Phytochemistry.

[11] Kumar R, Duffy S, Avery V M, Carroll A R, Davis R A (2018). J Nat Prod.

[12] Pedersen H A, Ndi C, Semple S J, Buirchell B, Møller B L, Staerk D (2020). J Nat Prod.

[13] Zhao Y, Li T, Kjaerulff L, Venter H, Coriani S, Møller B L, Semple S, Staerk D (2023). J Nat Prod.

[14] Petersen M J, Liang C, Kjaerulff L, Ndi C, Semple S, Buirchell B, Coriani S, Møller B L, Staerk D (2022). Phytochemistry.

[15] Hye Jin K, Kye Jung S, Hyun Soo K, Donghyn K, Hyung Jun K, Young Deog H, Kee Dong Y (2023). Nat Prod Sci.

[16] Yamauchi S, Ina T, Kirikihira T, Masuda T (2004). Biosci, Biotechnol, Biochem.

[17] Umezawa T, Kuroda H, Isohata T, Higuchi T, Shimada M (1994). Biosci, Biotechnol, Biochem.

[18] Yuan M, Zhang G, Bai W, Han X, Li C, Bian S (2022). Oxid Med Cell Longevity.

[19] Yool A J, Ramesh S (2020). Front Pharmacol.

[20] Hwang B, Lee J, Liu Q-H, Woo E-R, Lee D G (2010). Molecules.

[21] Kang M-H, Naito M, Sakai K, Uchida K, Osawa T (1999). Life Sci.

[22] Hashim Y Z H-Y, Rowland I R, McGlynn H, Servili M, Selvaggini R, Taticchi A, Esposto S, Montedoro G, Kaisalo L, Wähälä K (2008). Int J Cancer.

[23] Varricchio A, Khan S, Price Z K, Davis R A, Ramesh S A, Yool A J (2023). Cancers.

[24] Chen S, Deng X, Sheng H, Rong Y, Zheng Y, Zhang Y, Lin J (2021). Mol Ther–Nucleic Acids.

[25] Yue F, Lan W, Zhang L, Lu F, Sun R, Ralph J (2021). Front Energy Res.

[26] Miyazawa M, Kasahara H, Kameoka H (1993). Phytochemistry.

[27] Lu Y, Xue Y, Liu J, Yao G, Li D, Sun B, Zhang J, Liu Y, Qi C, Xiang M (2015). J Nat Prod.

[28] Stomberg R, Langer V, Li S, Lundquist K (2001). Acta Crystallogr, Sect E: Struct Rep Online.

[29] Tietjen I, Cassel J, Register E T, Zhou X Y, Messick T E, Keeney F, Lu L D, Beattie K D, Rali T, Tebas P (2021). Antimicrob Agents Chemother.

[30] Wu W, Klockow J L, Zhang M, Lafortune F, Chang E, Jin L, Wu Y, Daldrup-Link H E (2021). Pharmacol Res.

[31] Weeratunga S, Hu N-J, Simon A, Hofmann A (2012). BMC Bioinf.

[32] Hayes S, Taki A C, Lum K Y, Byrne J J, Ekins M G, Gasser R B, Davis R A (2022). Beilstein J Org Chem.

[33] González A G, Estévez-Reyes R, Mato C (1989). J Nat Prod.

[34] Páska C, Innocenti G, Ferlin M, Kunvári M, László M (2002). Nat Prod Lett.

[35] Iida T, Nakano M, Ito K (1982). Phytochemistry.

[36] Sheldrick G M (2015). Acta Crystallogr, Sect C: Struct Chem.

[37] Macrae C F, Bruno I J, Chisholm J A, Edgington P R, McCabe P, Pidcock E, Rodriguez-Monge L, Taylor R, van de Streek J, Wood P A (2008). J Appl Crystallogr.

[38] Farrugia L J (1999). J Appl Crystallogr.

[39] Ligasová A, Koberna K (2021). Molecules.

[40] O'Brien J, Wilson I, Orton T, Pognan F (2000). Eur J Biochem.

[41] Justus C R, Leffler N, Ruiz-Echevarria M, Yang L V (2014). J Visualized Exp.

