# Supplementary material for: Anti-invasive and cytotoxic evaluation of a (+)-pinoresinol-based semisynthetic library against glioblastoma
Source: Beilstein J Org Chem. 2026 May 11;22:691–704. doi: 10.3762/bjoc.22.54 (PMC13181606; doi:10.3762/bjoc.22.54)

BEILSTEIN JOURNAL OF ORGANIC CHEMISTRY

Supporting Information

for

Anti-invasive and cytotoxic evaluation of a (+)-pinoresinol-based semisynthetic library against glioblastoma

Chen Zhang, Kah Yean Lum, Jonathan M. White, Paul I. Forster, Nicholas Booth, Sunita A. Ramesh and Rohan A. Davis

Beilstein J. Org. Chem. **2026,** *22,* 691–704. doi:10.3762/bjoc.22.54

Compound characterization data

License and Terms: This is a supporting information file under the terms of the Creative Commons Attribution License (https://creativecommons.org/licenses/by/4.0). Please note that the reuse, redistribution and reproduction in particular requires that the author(s) and source are credited and that individual graphics may be subject to special legal provisions.
The license is subject to the *Beilstein Journal of Organic Chemistry* terms and conditions: (https://www.beilstein-journals.org/bjoc/terms)

Table of contents

NMR data for salicifoliol (**1**) in $CDCl_3$ S4

^{1}H NMR spectrum of salicifoliol (**1**) in $CDCl_3$ S5

^{13}C NMR spectrum of salicifoliol (**1**) in $CDCl_3$ S5

COSY spectrum of salicifoliol (**1**) in $CDCl_3$ S6

HSQC spectrum of salicifoliol (**1**) in $CDCl_3$ S6

HMBC spectrum of salicifoliol (**1**) in $CDCl_3$ S7

ROESY spectrum of salicifoliol (**1**) in $CDCl_3$ S7

NMR data for (+)-pinoresinol (**2**) in $CDCl_3$ S8

^{1}H NMR spectrum of (+)-pinoresinol (**2**) in $CDCl_3$ S9

^{13}C NMR spectrum of (+)-pinoresinol (**2**) in $CDCl_3$ S9

COSY spectrum of (+)-pinoresinol (**2**) in $CDCl_3$ S10

HSQC spectrum of (+)-pinoresinol (**2**) in $CDCl_3$ S10

HMBC spectrum of (+)-pinoresinol (**2**) in $CDCl_3$ S11

ROESY spectrum of (+)-pinoresinol (**2**) in $CDCl_3$ S11

NMR data for (+)-eudesmin (**3**) in $CDCl_3$ S12

^{1}H NMR spectrum of (+)-eudesmin (**3**) in $CDCl_3$ S13

^{13}C NMR spectrum of (+)-eudesmin (**3**) in $CDCl_3$ S13

COSY spectrum of (+)-eudesmin (**3**) in $CDCl_3$ S14

HSQC spectrum of (+)-eudesmin (**3**) in $CDCl_3$ S14

HMBC spectrum of (+)-eudesmin (**3**) in $CDCl_3$ S15

ROESY spectrum of (+)-eudesmin (**3**) in $CDCl_3$ S15

NMR data for (+)-phillygenin (**4**) in $CDCl_3$ S16

^{1}H NMR spectrum of (+)-phillygenin (**4**) in $CDCl_3$ S17

^{13}C NMR spectrum of (+)-phillygenin (**4**) in $CDCl_3$ S17

COSY spectrum of (+)-phillygenin (**4**) in $CDCl_3$ S18

HSQC spectrum of (+)-phillygenin (**4**) in $CDCl_3$ S18

HMBC spectrum of (+)-phillygenin (**4**) in $CDCl_3$ S19

ROESY spectrum of (+)-phillygenin (**4**) in $CDCl_3$ S19

NMR data for (+)-5,5'-dibromopinoresinol (**5**) in $CDCl_3$ S20

^{1}H NMR spectrum of (+)-5,5'-dibromopinoresinol (**5**) in $CDCl_3$ S21

^{13}C NMR spectrum of (+)-5,5'-dibromopinoresinol (**5**) in $CDCl_3$ S21

COSY spectrum of (+)-5,5'-dibromopinoresinol (**5**) in $CDCl_3$ S22

HSQC spectrum of (+)-5,5'-dibromopinoresinol (**5**) in $CDCl_3$ S22

HMBC spectrum of (+)-5,5'-dibromopinoresinol (**5**) in $CDCl_3$ S23

ROESY spectrum of (+)-5,5'-dibromopinoresinol (**5**) in $CDCl_3$ S23

NMR data for (+)-4,4'-di(3,3-dimethylbutanoyl)pinoresinol (**6**) in $CDCl_3$ S24

^{1}H NMR spectrum of (+)-4,4'-di(3,3-dimethylbutanoyl)pinoresinol (**6**) in $CDCl_3$ S25

^{13}C NMR spectrum of (+)-4,4'-di(3,3-dimethylbutanoyl)pinoresinol (**6**) in $CDCl_3$ S25

COSY spectrum of (+)-4,4'-di(3,3-dimethylbutanoy)lpinoresinol (**6**) in $CDCl_3$ S26

HSQC spectrum of (+)-4,4'-di(3,3-dimethylbutanoy)lpinoresinol (**6**) in $CDCl_3$ S26

HMBC spectrum of (+)-4,4'-di(3,3-dimethylbutanoy)lpinoresinol (**6**) in $CDCl_3$ S27

ROESY spectrum of (+)-4,4'-di(3,3-dimethylbutanoy)lpinoresinol (**6**) in $CDCl_3$ S27

NMR data for (+)-4,4'-dipivaloylpinoresinol (**7**) in $CDCl_3$ S28

^{1}H NMR spectrum of (+)-4,4'-dipivaloylpinoresinol (**7**) in $CDCl_3$ S29

^{13}C NMR spectrum of (+)-4,4'-dipivaloylpinoresinol (**7**) in $CDCl_3$ S29

COSY spectrum of (+)-4,4'-dipivaloylpinoresinol (**7**) in $CDCl_3$ S30

HSQC spectrum of (+)-4,4'-dipivaloylpinoresinol (**7**) in $CDCl_3$ S30

HMBC spectrum of (+)-4,4'-dipivaloylpinoresinol (**7**) in $CDCl_3$ S31

ROESY spectrum of (+)-4,4'-dipivaloylpinoresinol (**7**) in $CDCl_3$ S31

UHPLC–MS data of salicifoliol (**1**) S32

UHPLC–MS data of (+)-pinoresinol (**2**) S33

UHPLC–MS data of (+)-eudesmin (**3**) S34

UHPLC–MS data of (+)-phillygenin (**4**) S35

UHPLC–MS data of (+)-5,5'-dibromopinoresinol (**5**) S36

UHPLC–MS data of (+)-4,4'-di(3,3-dimethylbutanoyl)pinoresinol (**6**) S37

UHPLC–MS data of (+)-4,4'-dipivaloylpinoresinol (**7**) S38

HRESIMS of (+)-pinoresinol (**2**) S39

HRESIMS of (+)-eudesmin (**3**) S40

HRESIMS of (+)-phillygenin (**4**) S41

HRESIMS of (+)-5,5'-dibromopinoresinol (**5**) S42

HRESIMS of (+)-4,4'-di(3,3-dimethylbutanoyl)pinoresinol (**6**) S43

HRESIMS of (+)-4,4'-dipivaloylpinoresinol (**7**) S44

NMR data comparison for (+)-5,5'-dibromopinoresinol (**5**) and previously synthesised and reported racemic (±)-5,5'-dibromopinoresinol in acetone-d_6 .... S45

^{1}H NMR spectrum of (+)-5,5'-dibromopinoresinol (**5**) in acetone-d_6 .... S46

NMR data for salicifoliol (**1**) in $CDCl_3$[a]

Position	δ_H, mult (*J* in Hz)	δ_C, type	COSY	HMBC	ROESY
1		130.8, C			
2	6.88 (d, 1.9)	108.6, CH	6	4,6,7	3-OMe,7
3		147.1, C			
3-OMe	3.91 (s)	56.1, CH_3		3	2
4		146.0, C			
4-OH	5.65 (s)			4,5,6	
5	6.90 (d, 8.1)	114.6, CH	6	1,3	
6	6.80 (dd, 8.1, 1.9)	119.3, CH	2,5	2,4,7	7
7	4.62 (d, 6.9)	86.3, CH	8	1,2,6,8,9,11	2,6,8
8	3.12 (dddd, 9.1, 6.9, 6.9, 2.2)	48.3, CH	7,9a,9b, 11	1,7,11,10,12	6,9a,11
9a	4.50 (dd, 9.7, 6.9)	70.0, CH_2	8, 9b	7,8,10,11	8
9b	4.34 (dd, 9.7, 2.2)		8, 9a	7,8,10,11	
10		178.3, C			
11	3.44 (ddd, 9.1, 9.0, 3.8)	46.2, CH	8,12a, 12b	7,8,9,10,12	8, 12a
12a	4.35 (dd, 9.1, 9.0)	70.1, CH_2	11,12b	8,10,11	11
12b	4.18 (dd, 9.1, 3.8)		11,12a	8,10,11	

[a]Spectra recorded at 25 °C (800 MHz for ^1H and 200 MHz for ^{13}C).

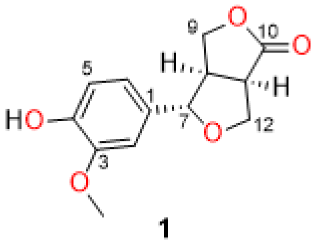

1

^{1}H NMR spectrum of salicifoliol (**1**) in $CDCl_3$

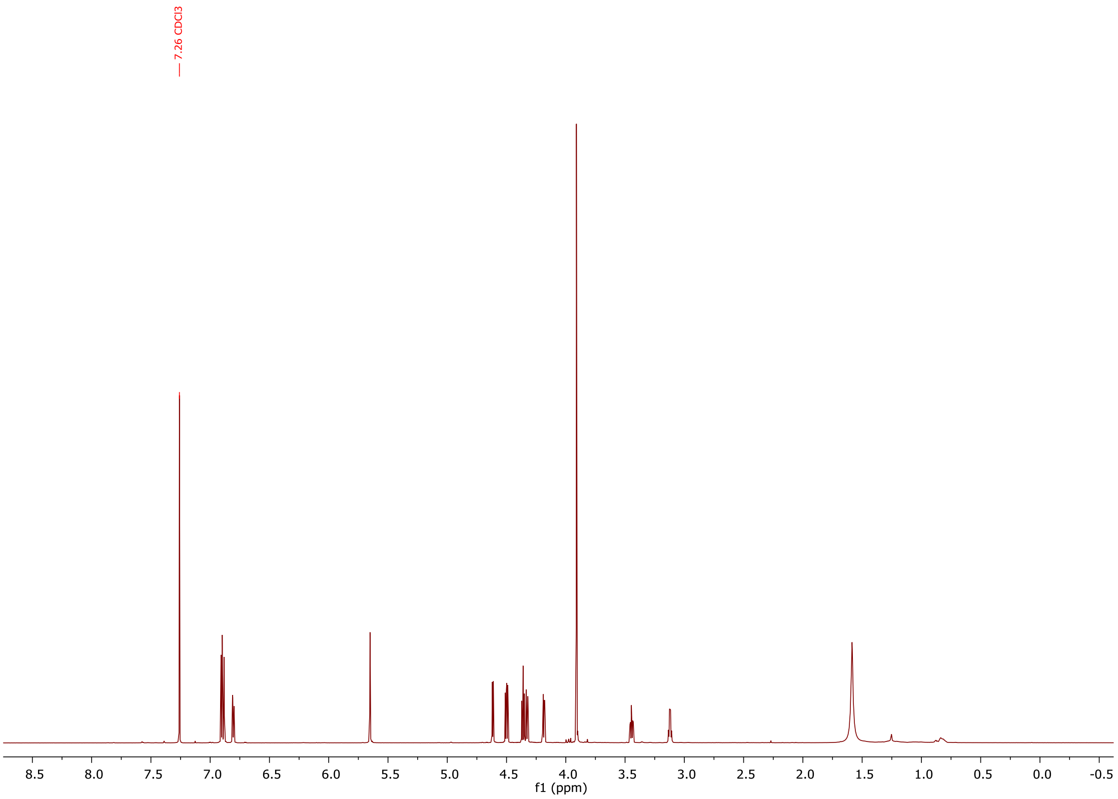

^{13}C NMR spectrum of salicifoliol (**1**) in $CDCl_3$

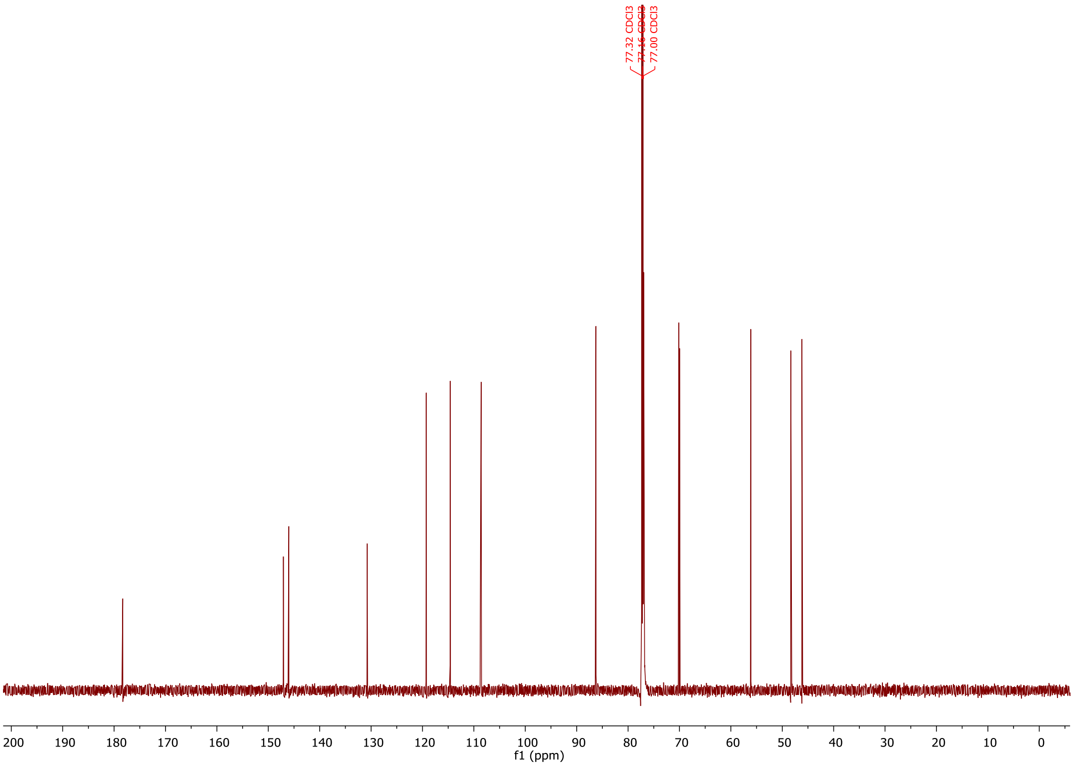

COSY spectrum of salicifoliol (**1**) in $CDCl_3$

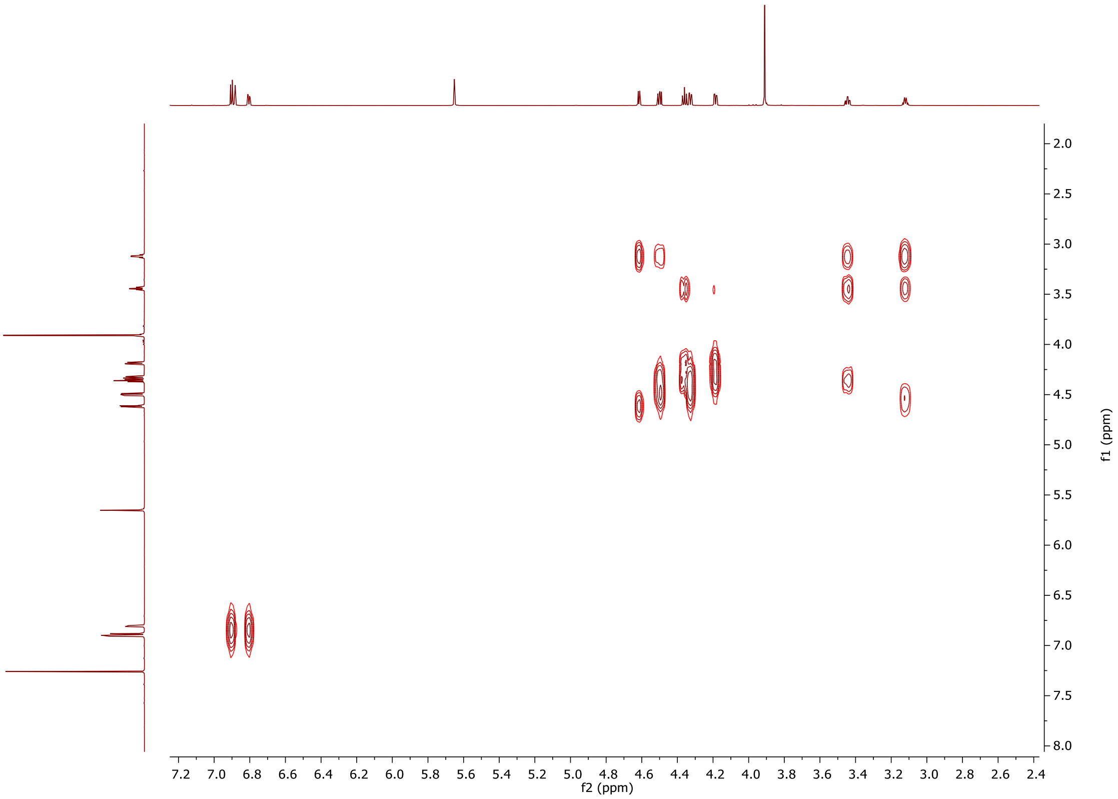

HSQC spectrum of salicifoliol (**1**) in $CDCl_3$

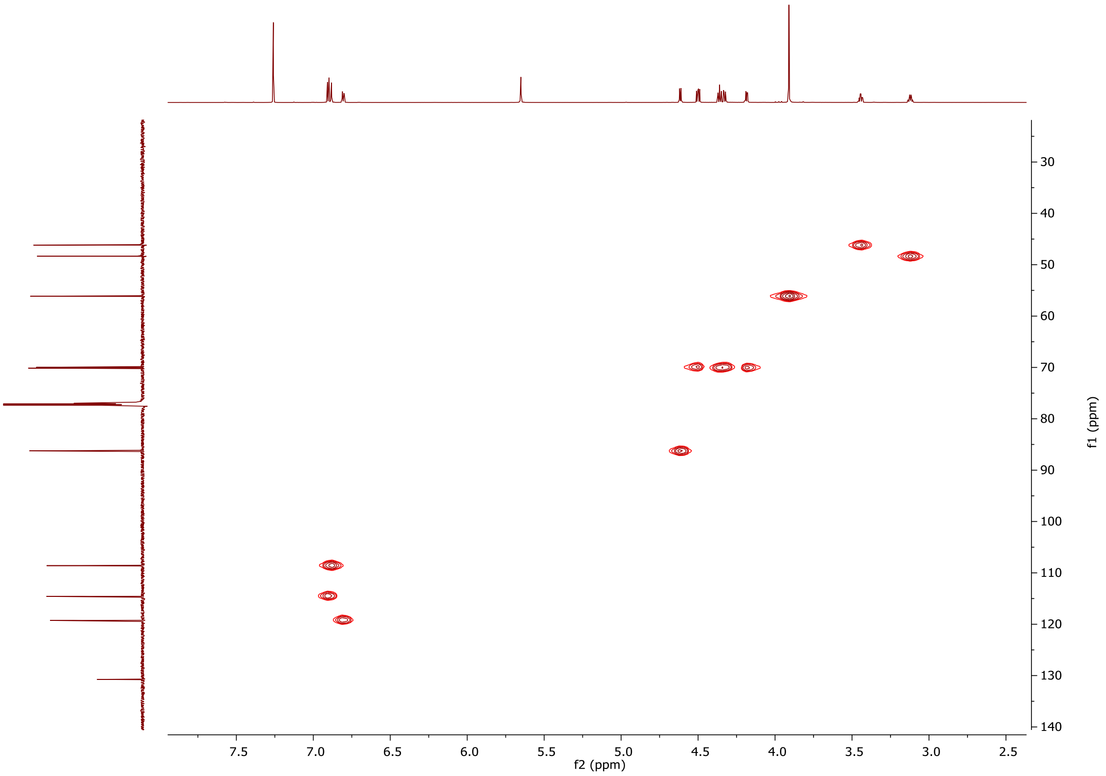

HMBC spectrum of salicifoliol (**1**) in $CDCl_3$

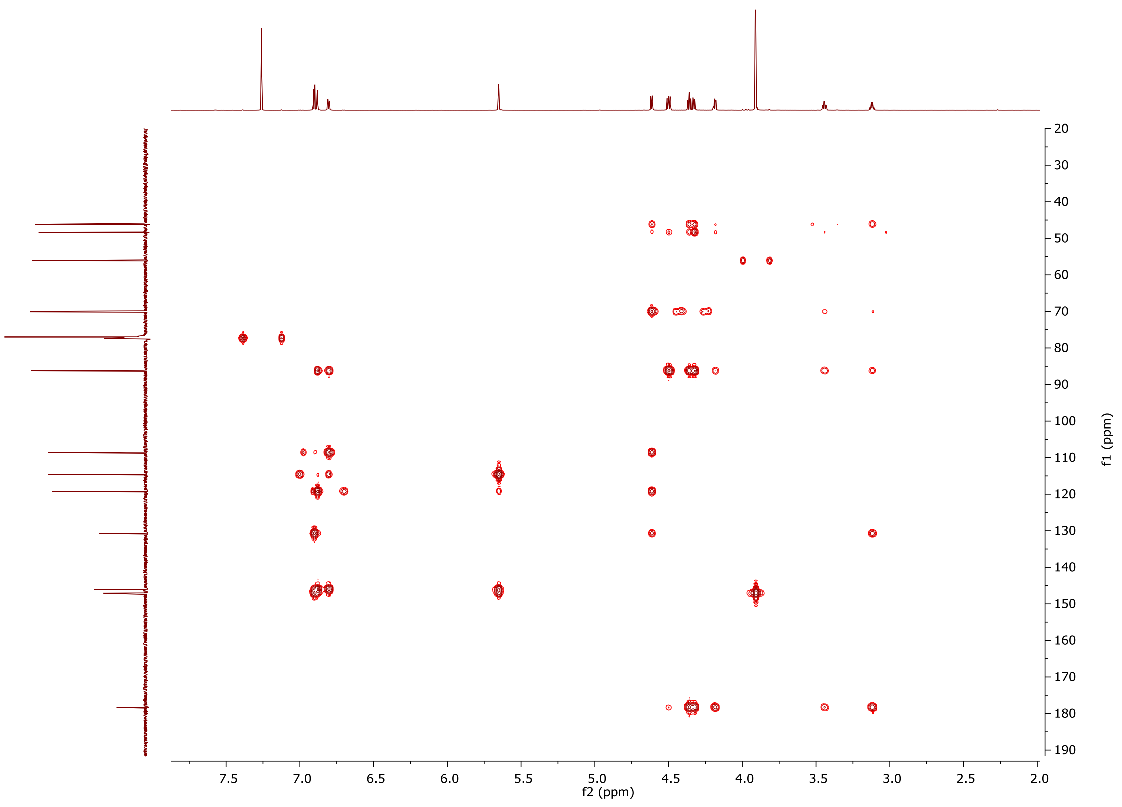

ROESY spectrum of salicifoliol (**1**) in $CDCl_3$

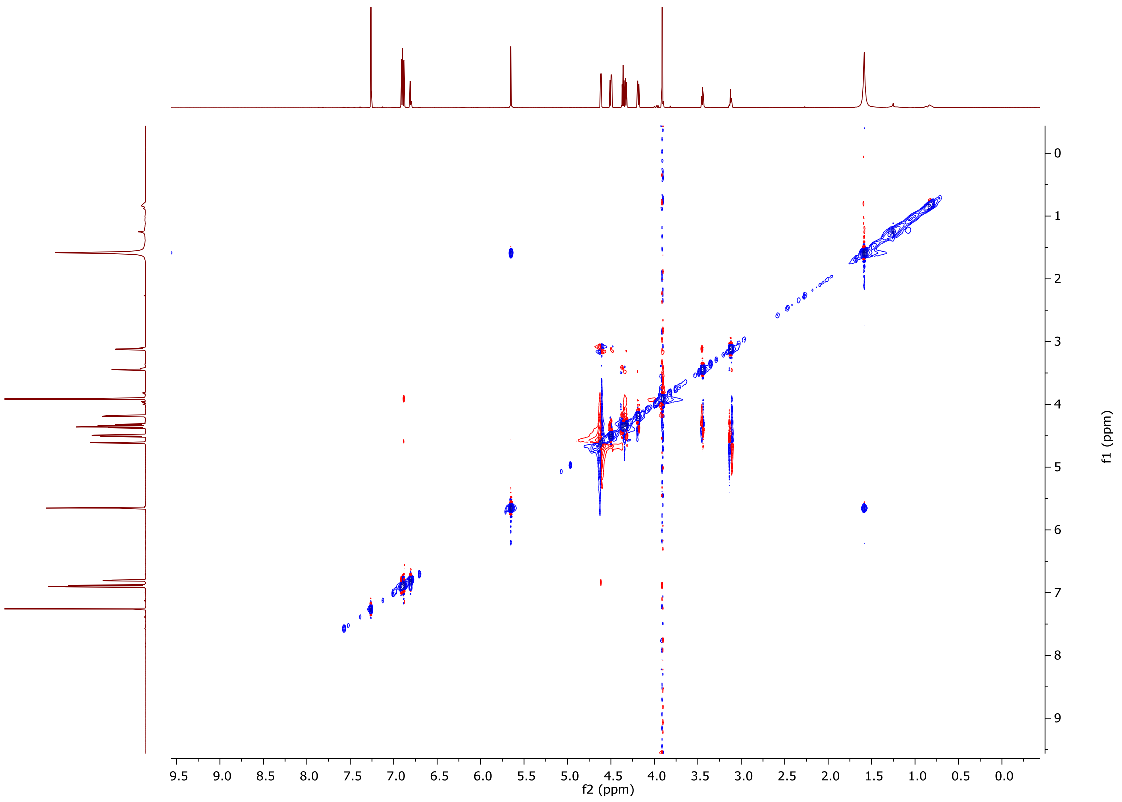

NMR data for (+)-pinoresinol (**2**) in $CDCl_3$[a]

Position	δ_H, mult (*J* in Hz)	δ_C, type	COSY	HMBC	ROESY
1		132.9, C			
2	6.90 (d, 1.9)	108.8, CH	6	4,6,7	3-OMe,7,8
3		146.9, C			
3-OMe	3.88 (s)	56.0, CH_3		3	2
4		145.3, C			
4-OH	5.80 (br s)				
5	6.88 (d, 8.0)	114.4, CH	6	1,3	
6	6.81 (dd, 8.0, 1.9)	119.1, CH	2,5	2,4,7	7,8
7	4.74 (d, 4.3)	86.0, CH	2,6,8	1,2,6,8,9	2,6,8,9b
8	3.11 (m)	54.2, CH	7,9a,9b	1,7,9,7',8',9'	2,6,7,9a
9a	4.25 (m)	71.7, CH_2	8, 9b	1[w],7,8,7'	8,9b
9b	3.89 (dd, 9.3, 3.7)		8, 9a	1[w],7,8,7'	7,9a
1'		132.9, C			
2'	6.90 (d, 1.9)	108.8, CH	6'	4',6',7'	3'-OMe,7',8'
3'		146.9, C			
3'-OMe	3.88 (s)	56.0, CH_3		3'	2'
4		145.3, C			
4'-OH	5.80 (br s)				
5'	6.88 (d, 8.0)	114.4, CH	6'	1',3'	
6'	6.81 (dd, 8.0, 1.9)	119.1, CH	2',5'	2',4',7'	7',8'
7'	4.74 (d, 4.3)	86.0, CH	2',6',8'	1',2',6',8',9'	2',6',8',9'b
8'	3.11 (m)	54.2, CH	7', 9'a, 9'b	1',7',9',7,8,9	2',6',7',9'a
9'a	4.25 (m)	71.7, CH_2	8', 9'b	1'[w],7',8',7	8',9'b
9'b	3.89 (dd, 9.3, 3.7)		8', 9'a	1'[w],7',8',7	7',9'a

[a]Spectra recorded at 25 °C (800 MHz for ^1H and 200 MHz for ^{13}C); [w]weak correlation.

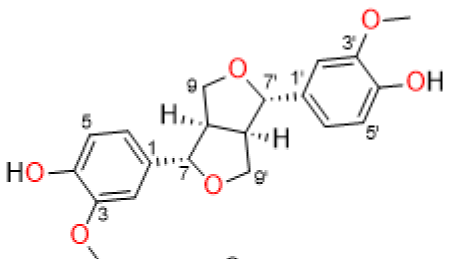

2

^{1}H NMR spectrum of (+)-pinoresinol (**2**) in $CDCl_3$

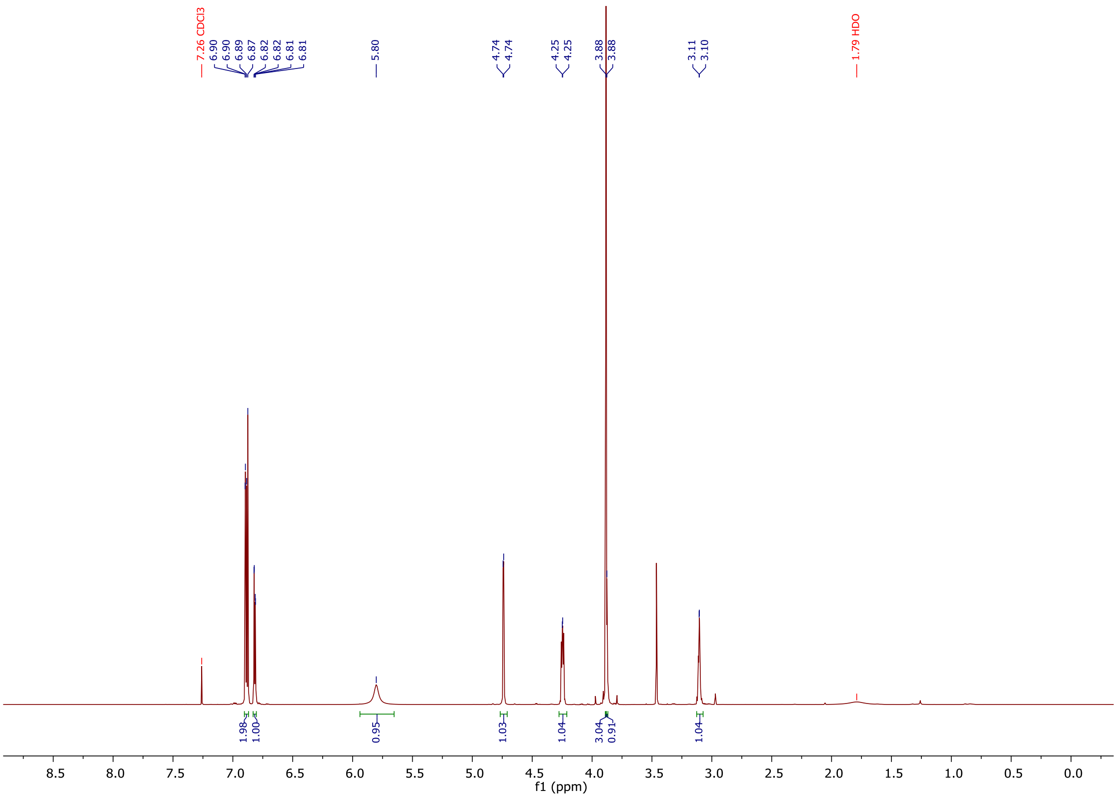

^{13}C NMR spectrum of (+)-pinoresinol (**2**) in $CDCl_3$

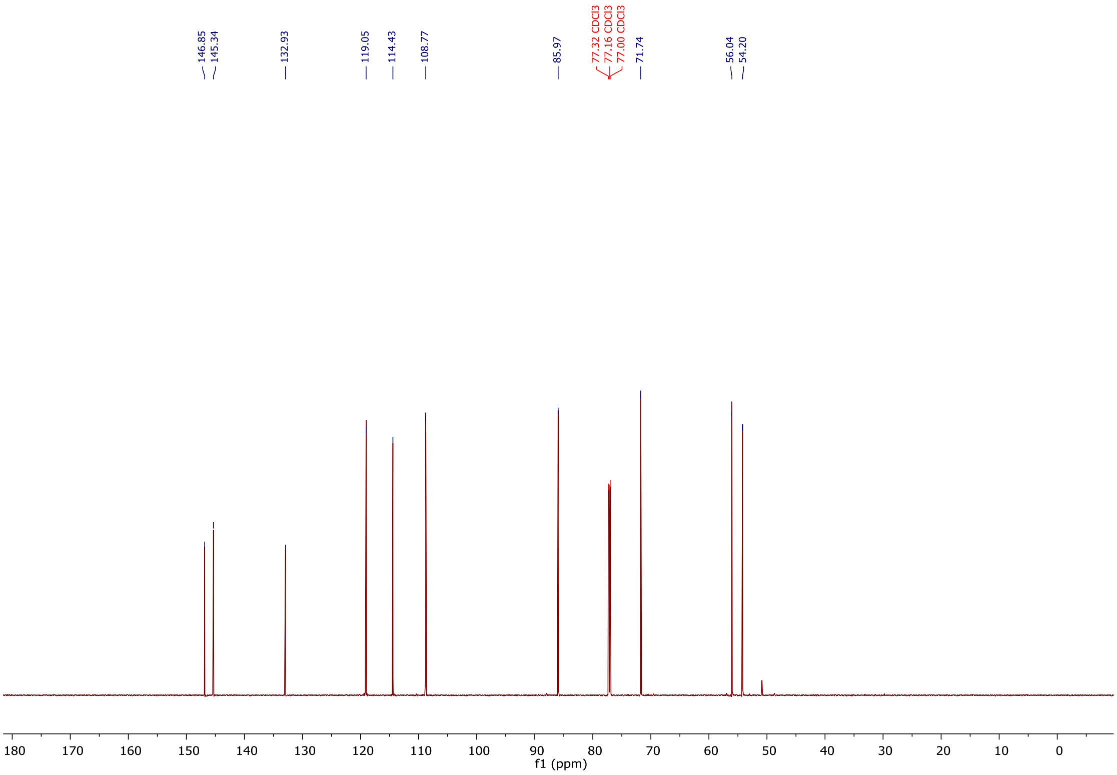

COSY spectrum of (+)-pinoresinol (**2**) in $CDCl_3$

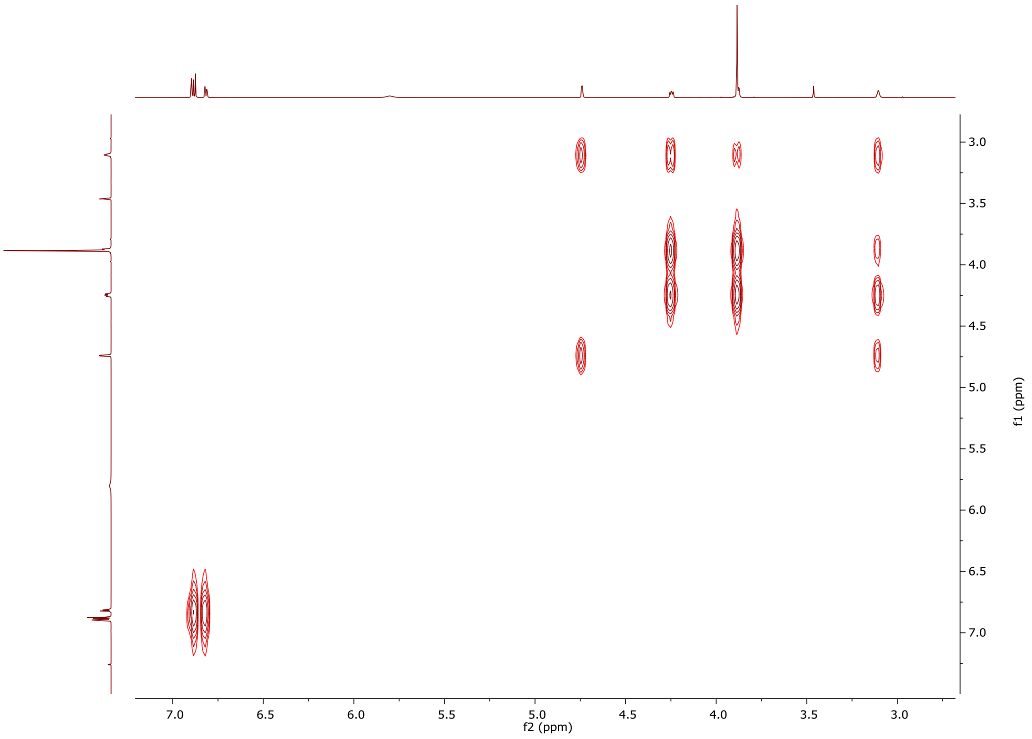

HSQC spectrum of (+)-pinoresinol (**2**) in $CDCl_3$

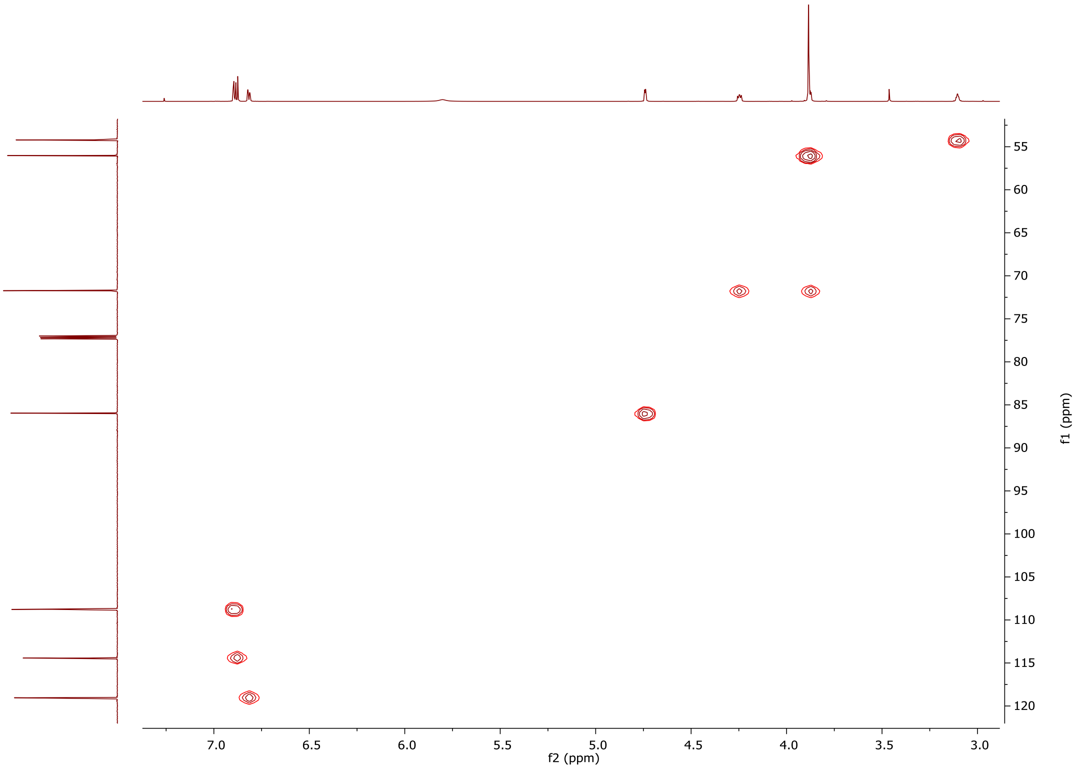

HMBC spectrum of (+)-pinoresinol (**2**) in $CDCl_3$

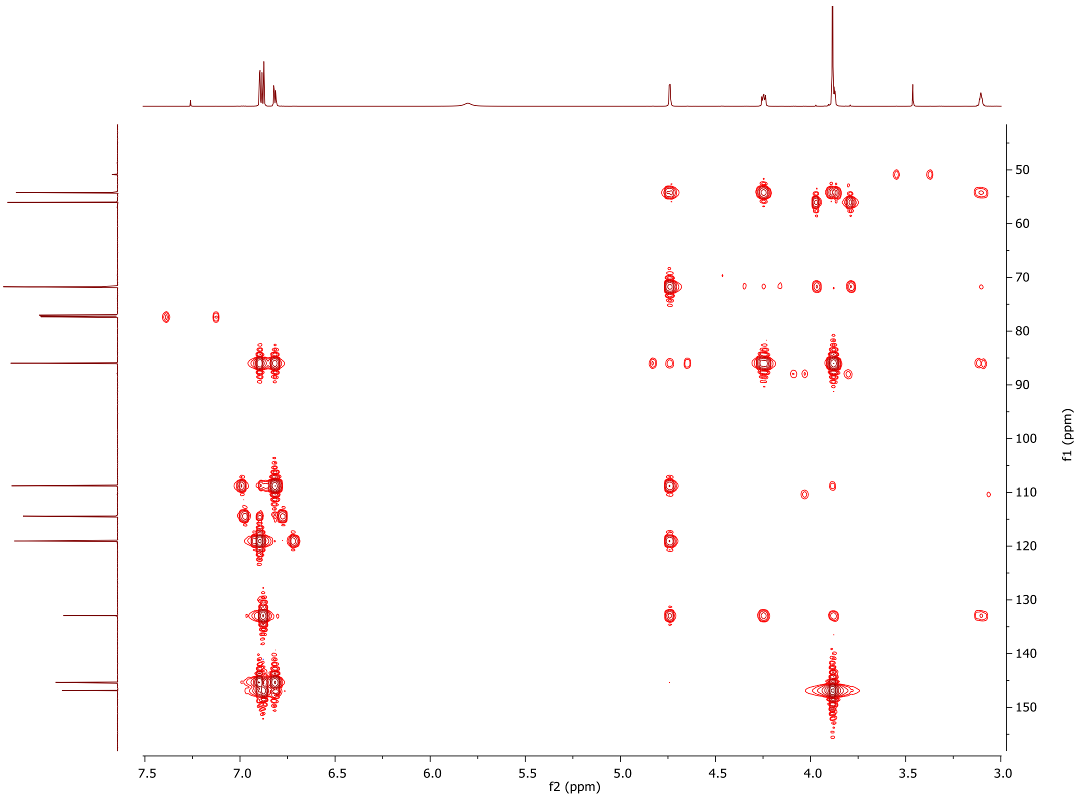

ROESY spectrum of (+)-pinoresinol (**2**) in $CDCl_3$

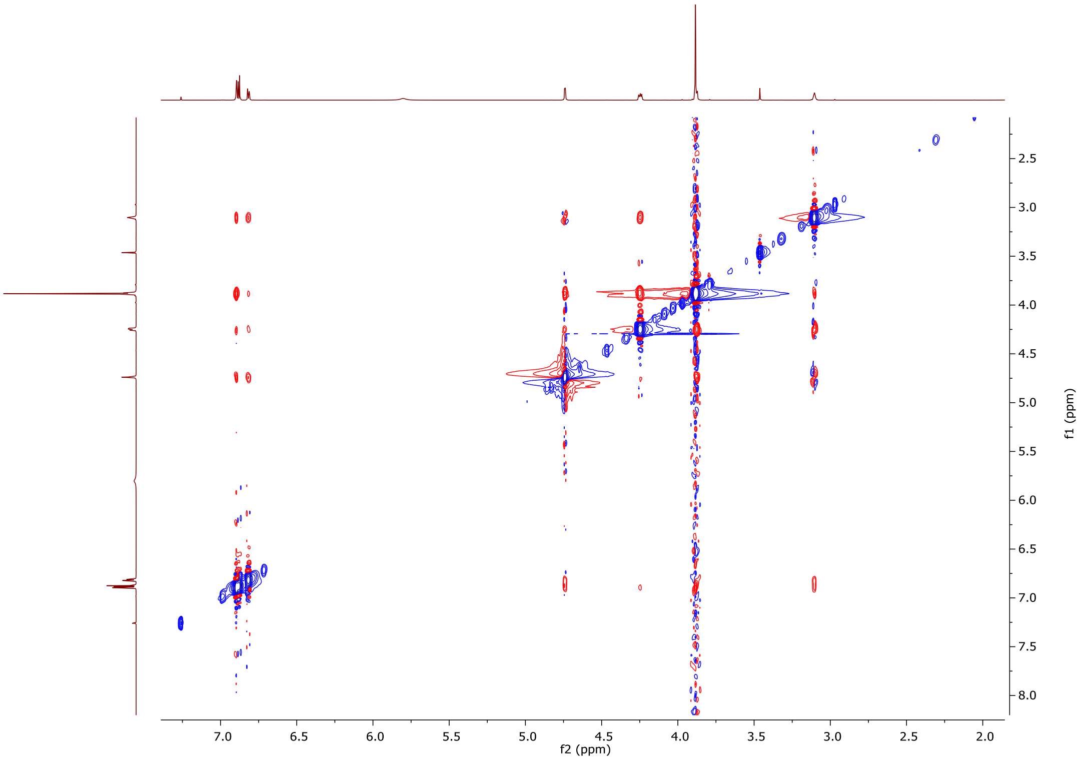

NMR data for (+)-eudesmin (**3**) in $CDCl_3$[a]

Position	δ_H, mult (*J* in Hz)	δ_C, type	COSY	HMBC	ROESY
1		133.7, C			
2	6.91 (d, 1.8)	109.4, CH	6,7	1,4,6,7	3-OMe,7,8
3		149.3, C			
3-OMe	3.90 (s)	56.09[b], CH_3		3	2
4		148.8, C			
4-OMe	3.87 (s)	56.06[b], CH_3		4	5
5	6.85 (d, 8.1)	111.2, CH	6	1,3	4-OMe,7,8
6	6.88 (dd, 8.1, 1.8)	118.4, CH	2,7	2,4,7	7,8,9a
7	4.76 (d, 4.3)	85.9, CH	2,6,8	1,2,6,8,9	2,6,8,9b
8	3.11 (m)	54.3, CH	7,9a,9b	1,7,9,7',8',9'	2,6
9a	4.26 (m)	71.9, CH_2	8, 9b	7,8,7'	2,6,8
9b	3.89 (dd, 9.4, 4.2)		8, 9a	7,8,7'	7
1'		133.7, C			
2'	6.91 (d, 1.8)	109.4, CH	7'	1',4',6',7'	3'-OMe,7',8'
3'		149.3, C			
3'-OMe	3.90 (s)	56.09[b], CH_3		3'	2'
4		148.8, C			
4'-OMe	3.87 (s)	56.06[b], CH_3		4'	5'
5'	6.85 (d, 8.1)	111.2, CH		1',3'	4'-OMe,7',8'
6'	6.88 (dd, 8.1, 1.8)	118.4, CH	7'	2',4',7'	7',8',9'a
7'	4.76 (d, 4.3)	85.9, CH	2',6',8'	1',2',6',8',9'	2',6',8',9'b
8'	3.11 (m)	54.3, CH	7',9'a,9'b	1',7',9',7,8,9	2',6'
9'a	4.26 (m)	71.9, CH_2	8', 9'b	7',8',7	2',6',8'
9'b	3.89 (dd, 9.4, 4.2)		8', 9'a	7',8',7	7'

[a]Spectra recorded at 25 °C (800 MHz for ^1H and 200 MHz for ^{13}C); [b]interchangeable signal.

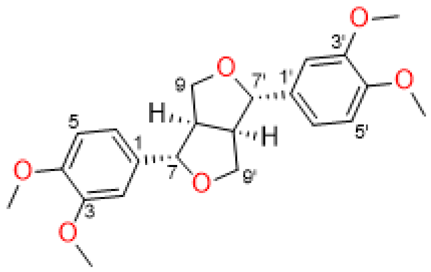

3

^1H NMR spectrum of (+)-eudesmin (**3**) in $CDCl_3$

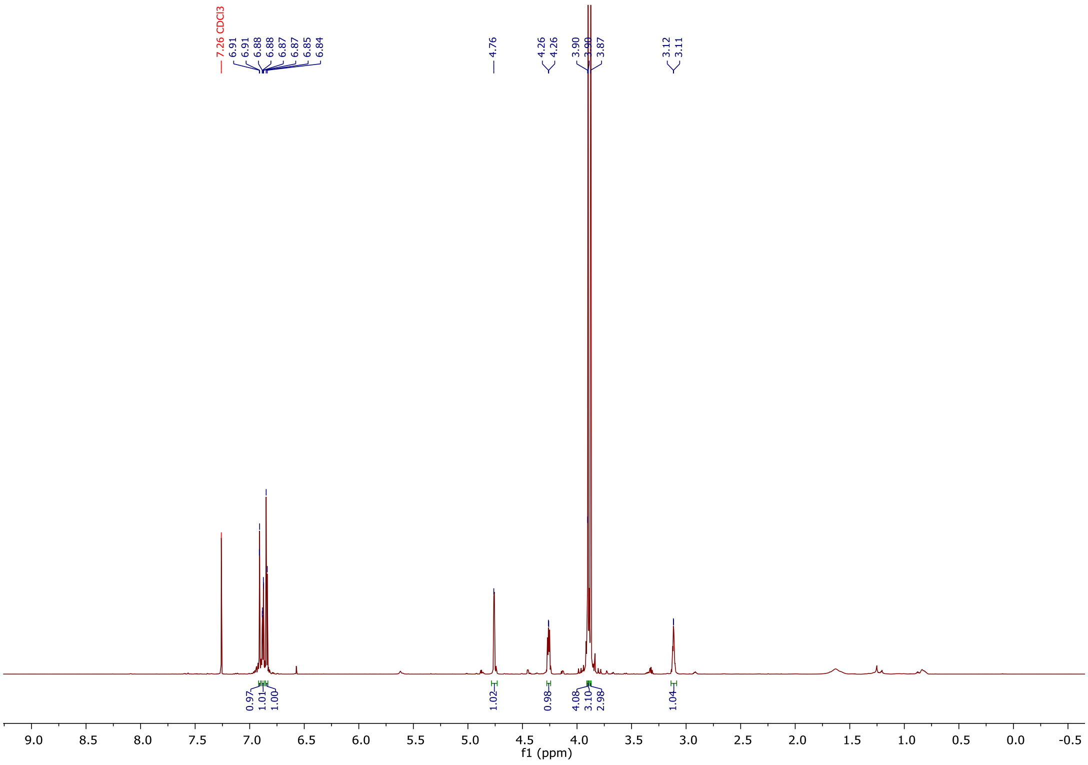

^{13}C NMR spectrum of (+)-eudesmin (**3**) in $CDCl_3$

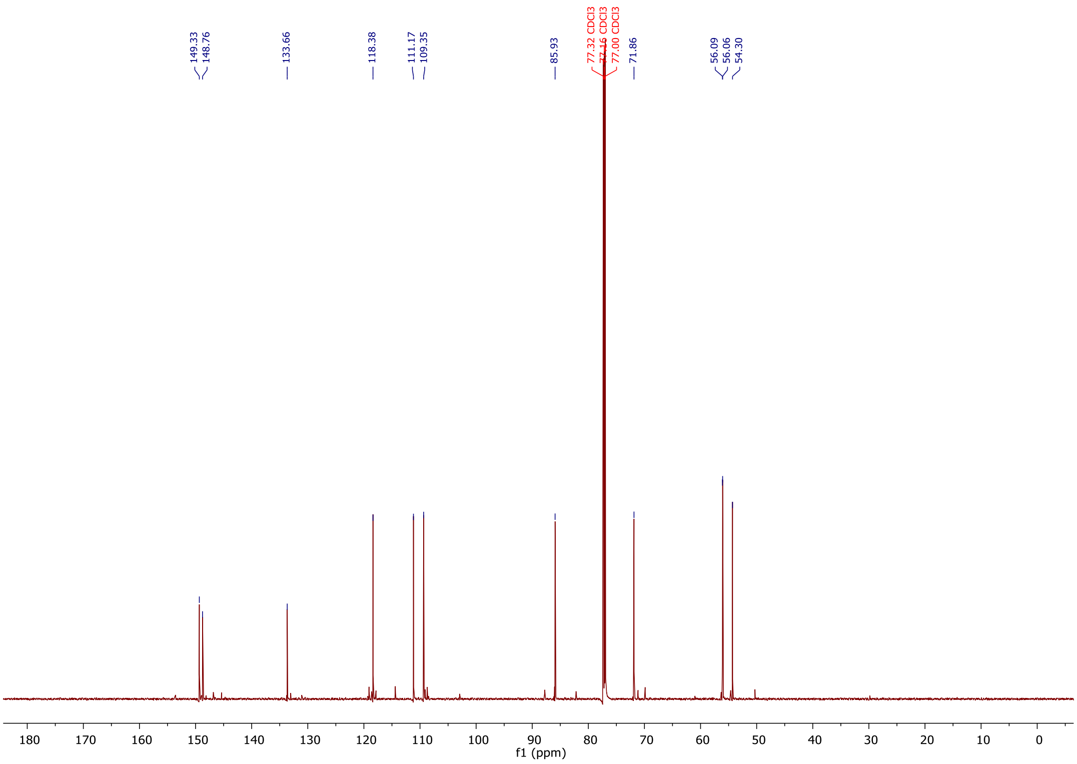

COSY spectrum of (+)-eudesmin (**3**) in $CDCl_3$

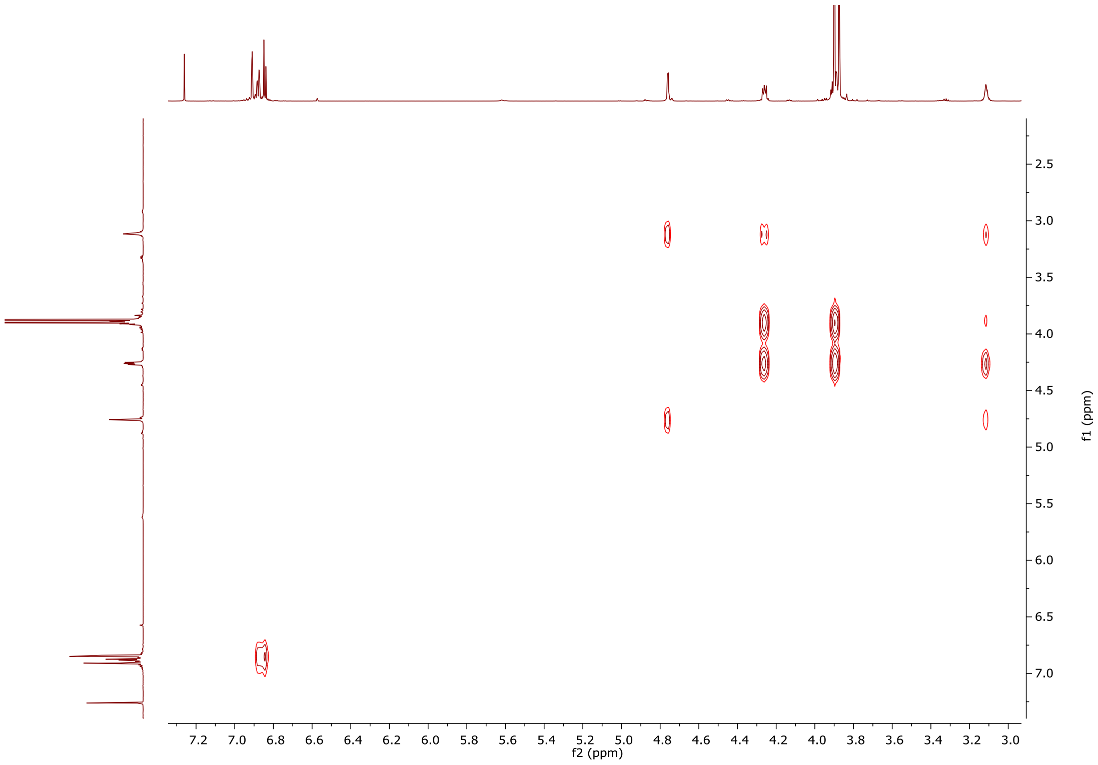

HSQC spectrum of (+)-eudesmin (**3**) in $CDCl_3$

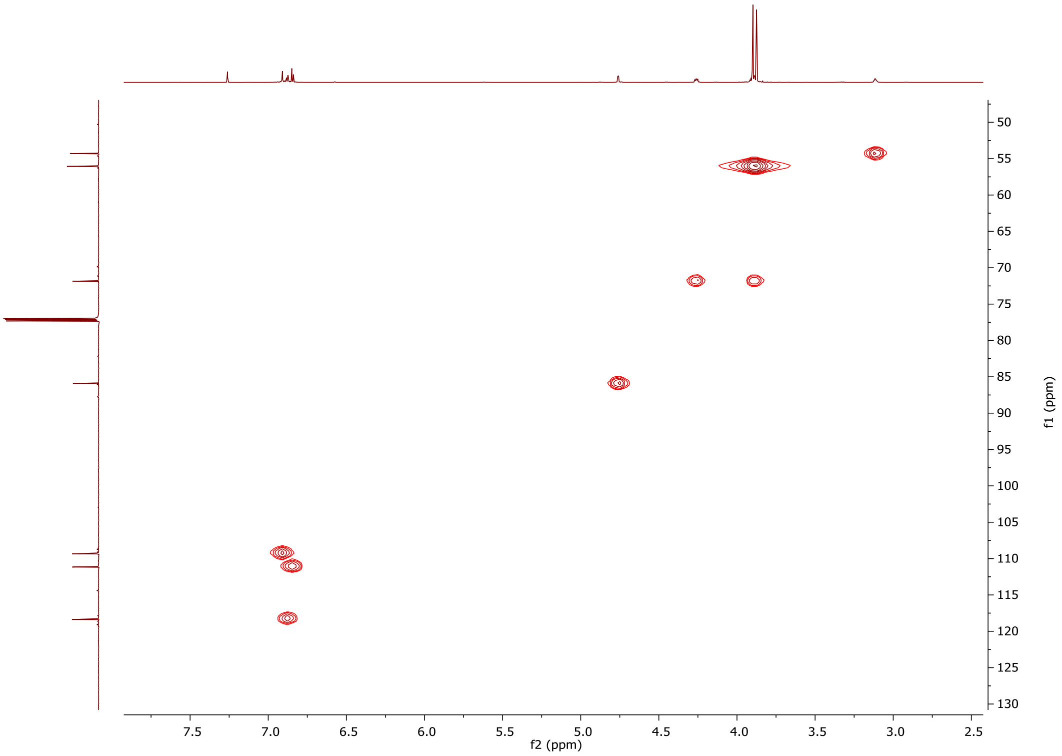

HMBC spectrum of (+)-eudesmin (**3**) in $CDCl_3$

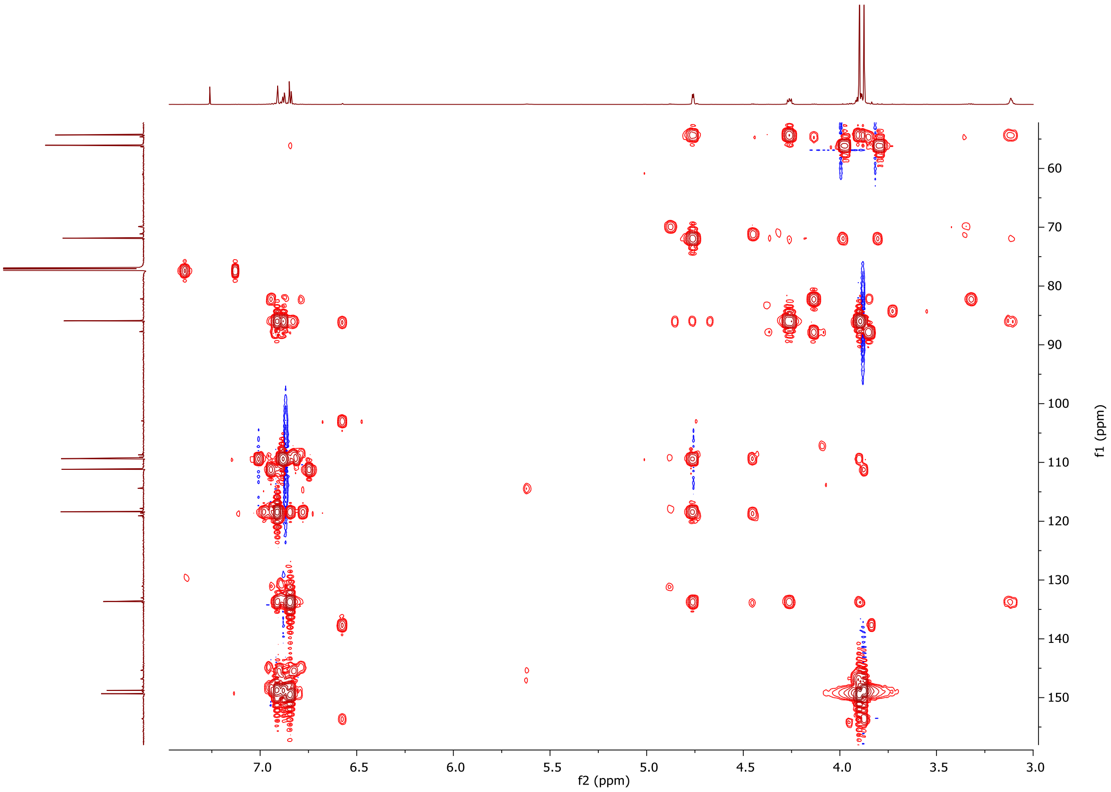

ROESY spectrum of (+)-eudesmin (**3**) in $CDCl_3$

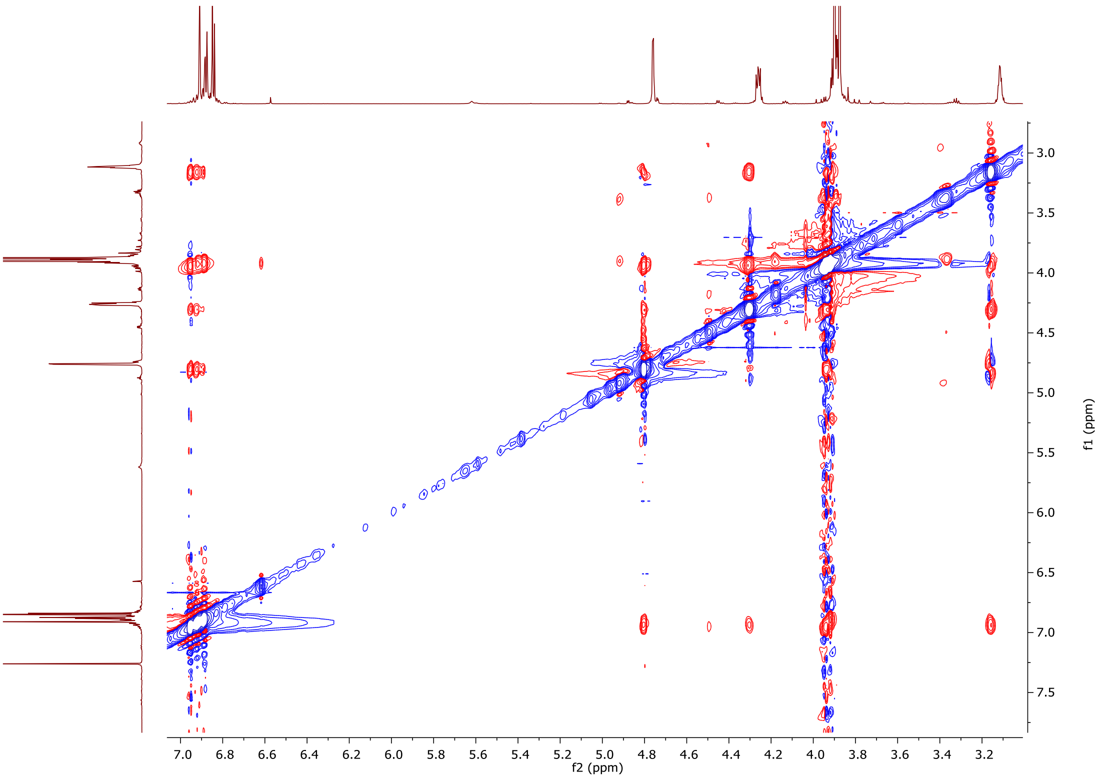

NMR data for (+)-phillygenin (**4**) in $CDCl_3$[a]

Position	δ_H, mult (*J* in Hz)	δ_C, type	COSY	HMBC	ROESY
1		133.1, C			
2	6.90 (d, 1.8)	109.4, CH	6	4,6,7	3-OMe,7,8
3		146.8, C			
3-OMe	3.91 (s)	56.11[b], CH_3		3	2
4		145.4, C			
4-OH	5.60 (s)			3,4,5	
5	6.89 (d, 8.0)	114.4, CH	6	1,3	
6	6.82 (dd, 8.0, 1.8)	119.1, CH	2,5	2,4,7	7,8
7	4.74 (d, 4.5)	86.0, CH	8	2,9	2,8,9b
8	3.11 (m)	54.32[c], C	7,9a,9b	7[w],8'[w]	2,7,9a,9b
9a	4.26 (m)	71.9, CH_2	8,9b	7,8,7'	2,8
9b	3.89 (m)		8,9a	7,8,7'	7,8
1'		133.7, C			
2'	6.91 (d, 1.8)	108.7, CH	6'	4',6',7'	3'-OMe,7',8'
3'		149.3, C			
3'-OMe	3.90 (s)	56.11[b], CH_3		3'	2'
4		148.8, C			
4'-OMe	3.88 (s)	56.08[b], CH_3		4'	5'
5'	6.84 (d, 8.2)	111.2, CH	6'	1',3'	
6'	6.87 (dd, 8.2, 1.8)	118.4, CH	2',5'	2',4',7'	7',8'
7'	4.76 (d, 4.5)	85.9, CH	8'	2',9'	
8'	3.10 (m)	54.31[c], C	7',9'a,9'b	7'[w],8[w]	2',7',9'a,9'b
9'a	4.25 (m)	71.8, CH_2	8',9'b	7',8',7	2',8'
9'b	3.87 (m)		8',9'a	7',8',7	7',8'

[a]Spectra recorded at 25 °C (800 MHz for ^{1}H and 200 MHz for ^{13}C); [b,c]interchangeable signals; [w]weak correlation.

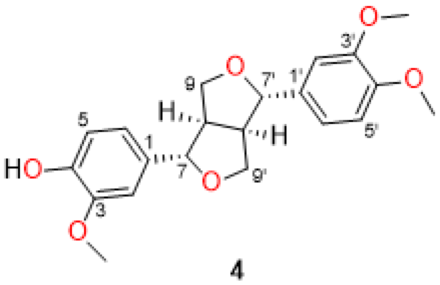

4

^1H NMR spectrum of (+)-phillygenin (**4**) in $CDCl_3$

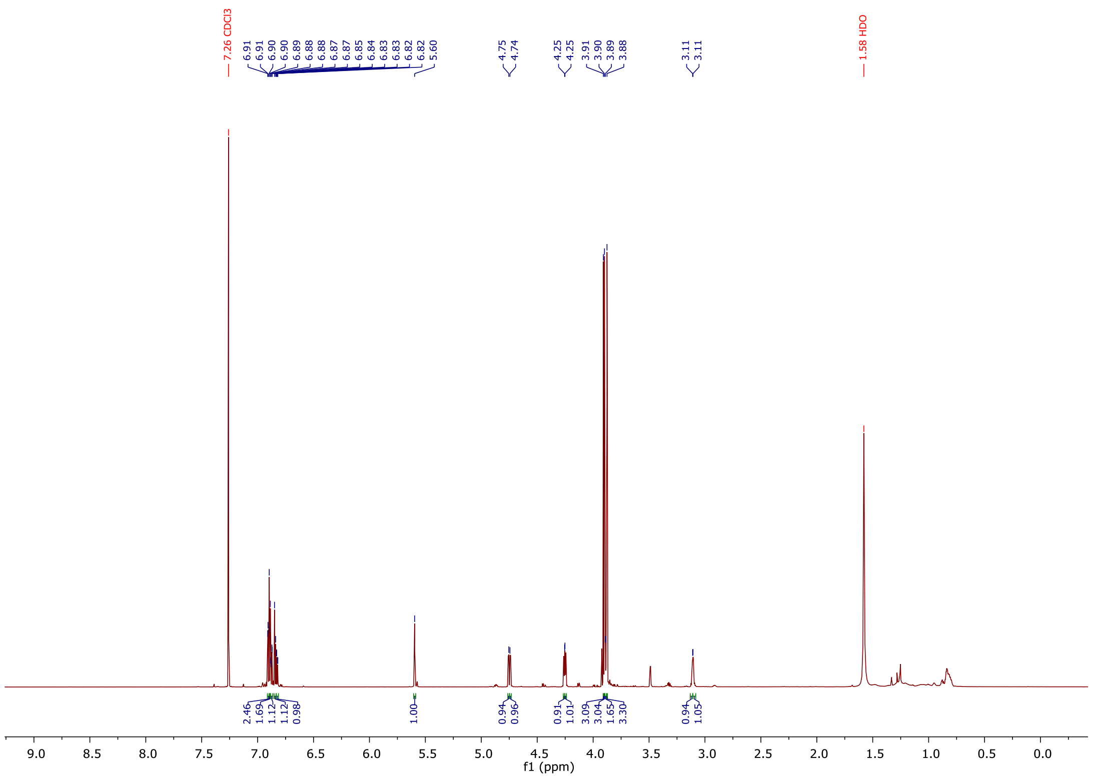

^{13}C NMR spectrum of (+)-phillygenin (**4**) in $CDCl_3$

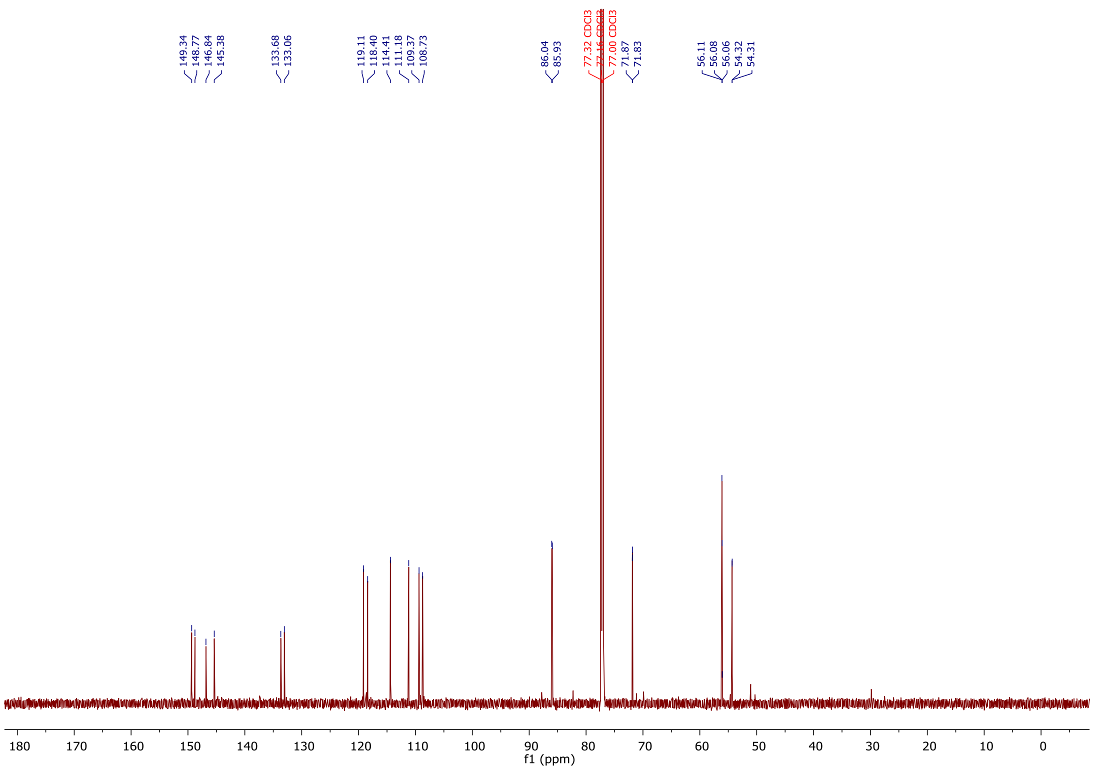

COSY spectrum of (+)-phillygenin (**4**) in $CDCl_3$

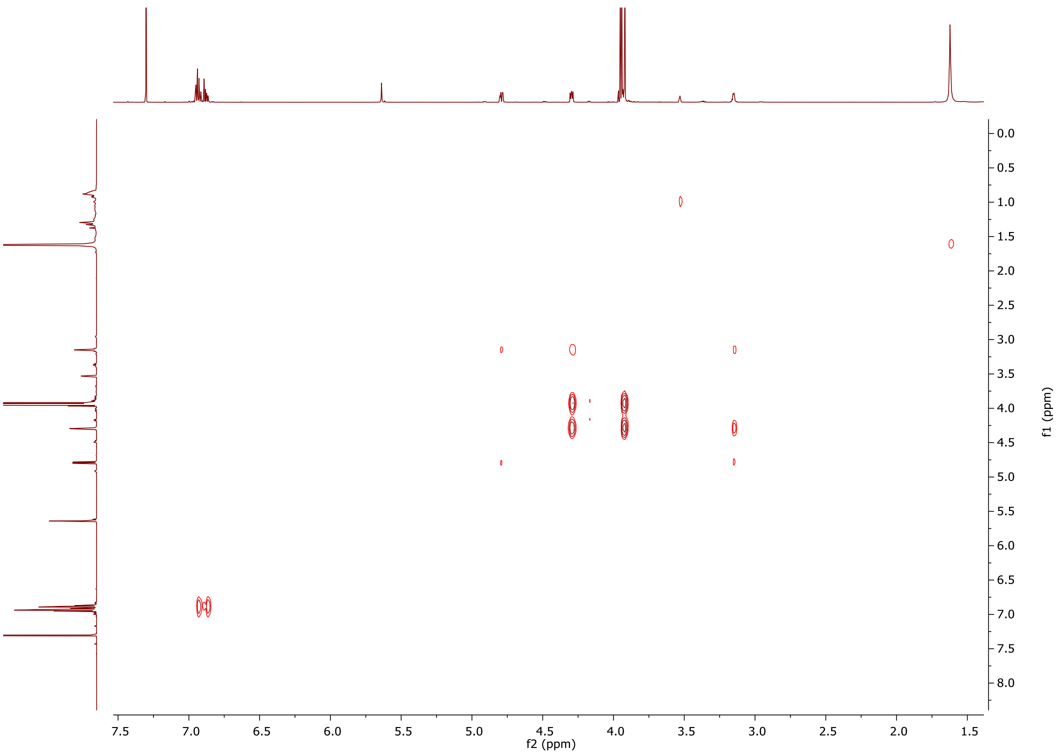

HSQC spectrum of (+)-phillygenin (**4**) in $CDCl_3$

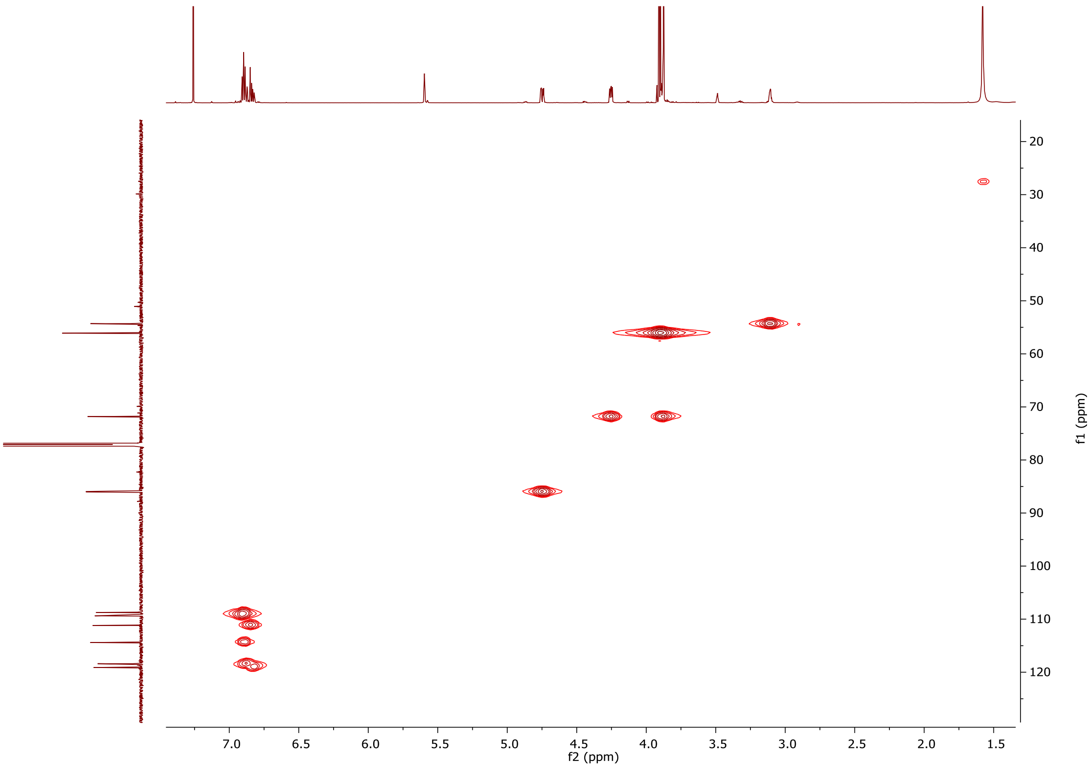

HMBC spectrum of (+)-phillygenin (**4**) in $CDCl_3$

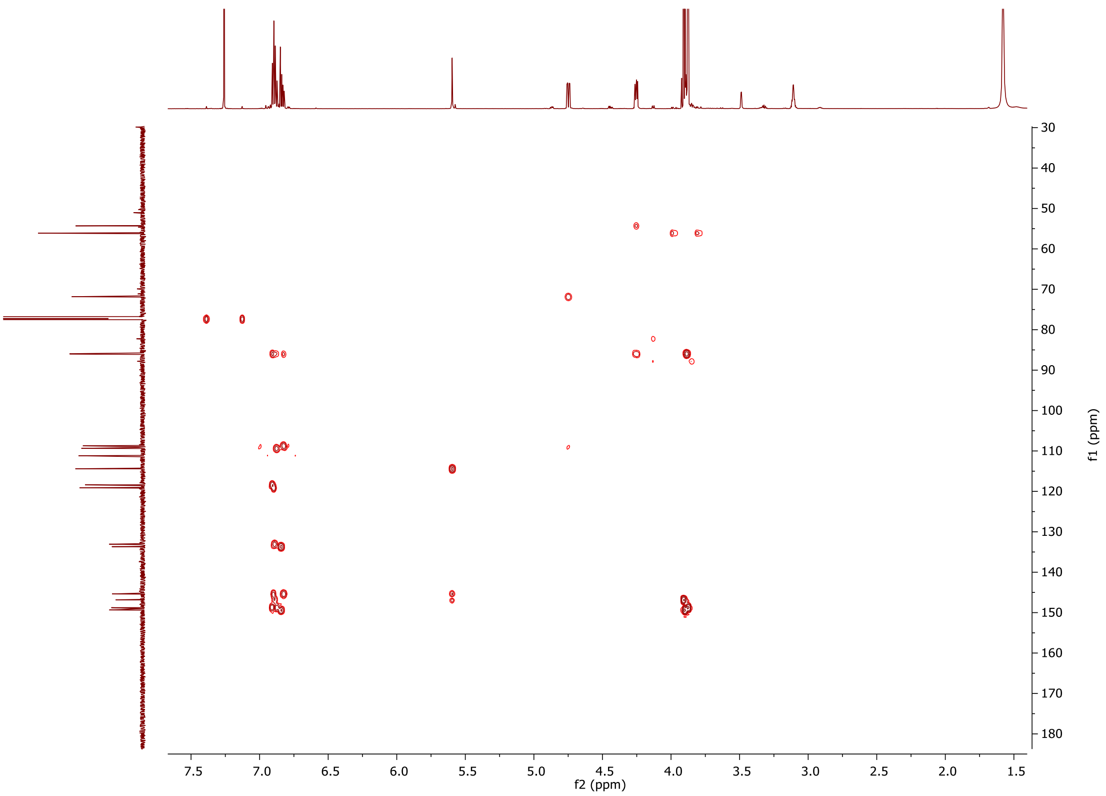

ROESY spectrum of (+)-phillygenin (**4**) in $CDCl_3$

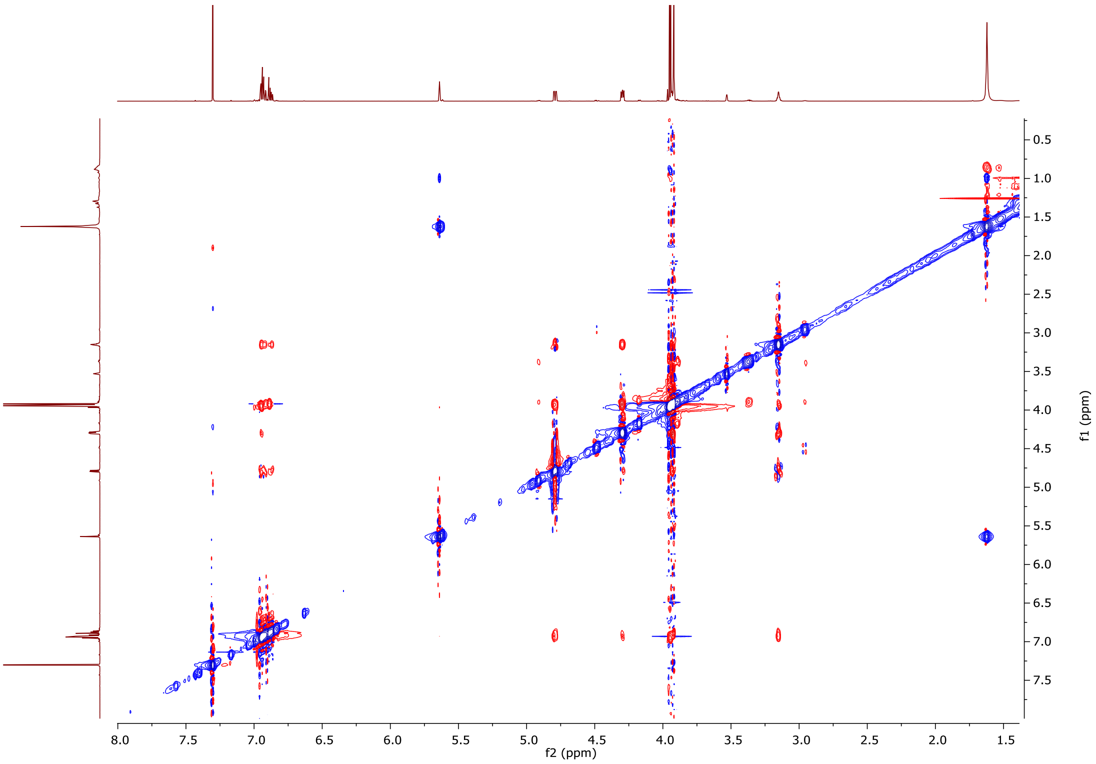

NMR data for (+)-5,5'-dibromopinoresinol (**5**) in $CDCl_3$[a]

Position	δ_H (mult, *J* in Hz)	δ_C, type	COSY	HMBC	ROESY
1		133.3, C			
2	6.83 (d, 1.8)	108.0, CH	6,7	1,3,4,5,6,7	3-OMe,7,8,9a
3		147.6, C			
3-OMe	3.92 (s)	56.6, CH_3		3	2
4		142.9, C			
4-OH	[b]				
5		108.4, C			
6	7.06 (dd, 1.8, 0.5)	122.3, CH	2,7	2,3,4,5,7	7,8,9a
7	4.75 (d, 4.2)	85.4, CH	2,6,8	1,2,6,8,9	2,6,8,9b
8	3.11 (m)	53.9, CH	7, 9a, 9b	1,7,9,7',8',9'	2,6,7,9a
9a	4.27 (m)	71.8, CH_2	8, 9b	1,7,8,7'	2,6,8,9b
9b	3.90 (dd, 9.3, 3.6)		8, 9a	7,7'	7
1'		133.3, C			
2'	6.83 (d, 1.8)	108.0, CH	6',7'	1',3',4',5',6',7'	3'-OMe,7',8',9'a
3'		147.6, C			
3'-OMe	3.92 (s)	56.6, CH_3		3'	2'
4'		142.9, C			
4'-OH	[b]				
5'		108.4, C			
6'	7.06 (dd, 1.8, 0.5)	122.3, CH	2',7'	2',3',4',5',7'	7',8',9'a
7'	4.75 (d, 4.2)	85.4, CH	2',6',8'	1',2',6',8',9'	2',6',8',9'b
8'	3.11 (m)	53.9, CH	7', 9'a, 9'b	1',7',9',7,8,9	2',6',7',9'a
9'a	4.27 (m)	71.8, CH_2	8', 9'b	1',7',8',7	2',6',8'
9'b	3.90 (dd, 9.3, 3.6)		8', 9'a	7',7	7'

[a]Spectra recorded at 25 °C (800 MHz for ^{1}H and 200 MHz for ^{13}C); [b]signal not observed.

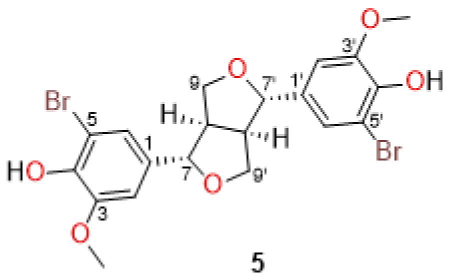

5

^{1}H NMR spectrum of (+)-5,5'-dibromopinoresinol (**5**) in $CDCl_3$

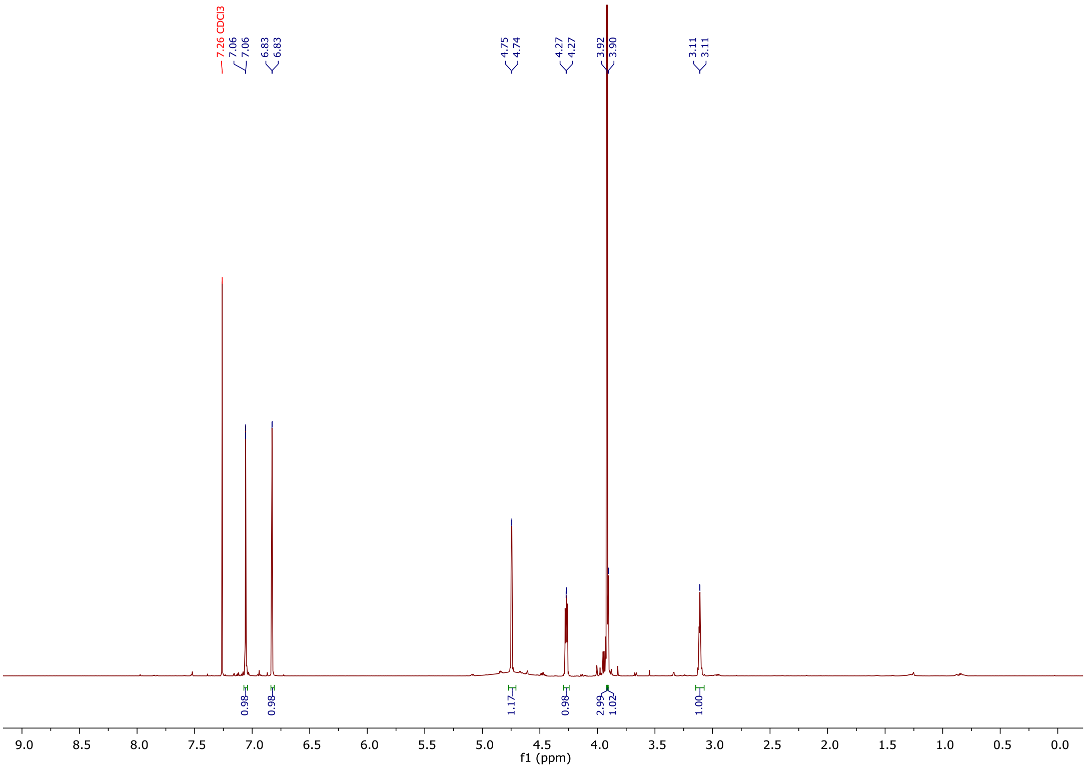

^{13}C NMR spectrum of (+)-5,5'-dibromopinoresinol (**5**) in $CDCl_3$

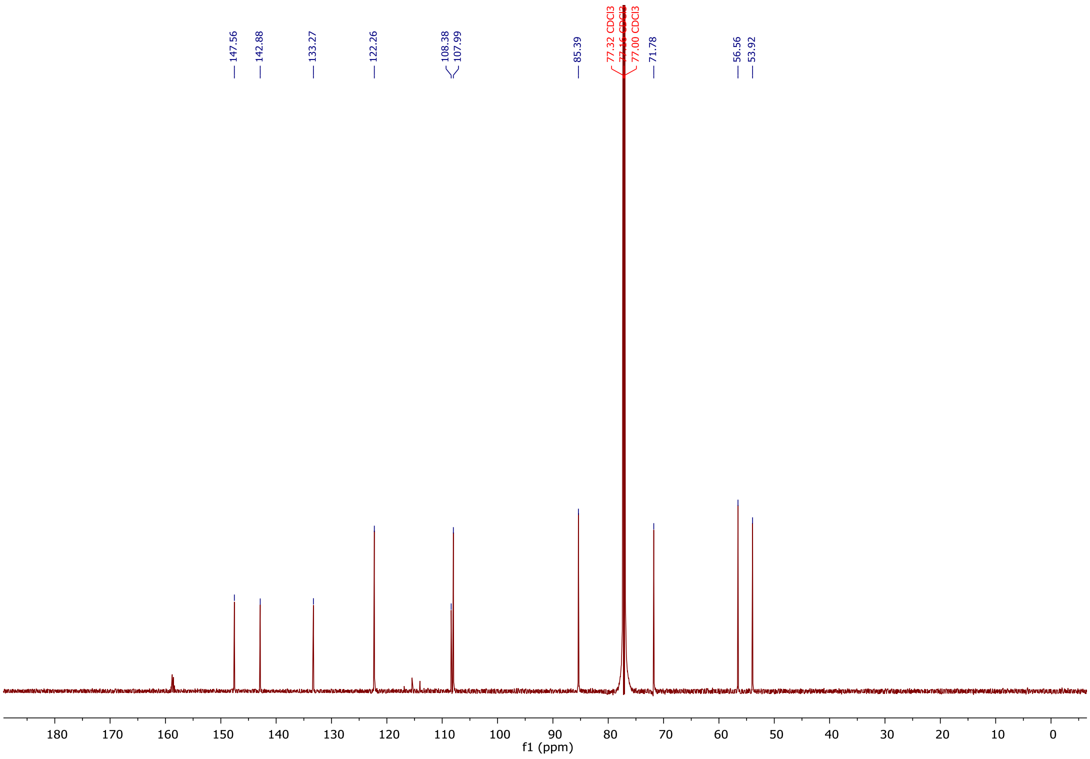

COSY spectrum of (+)-5,5'-dibromopinoresinol (**5**) in $CDCl_3$

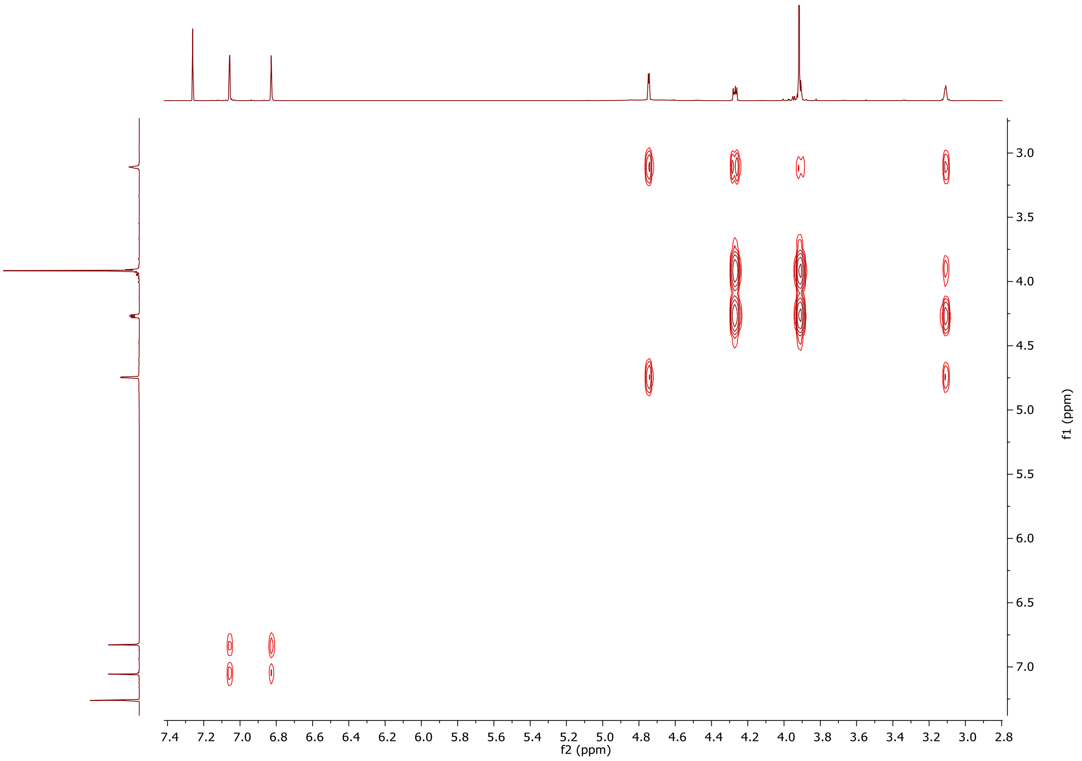

HSQC spectrum of (+)-5,5'-dibromopinoresinol (**5**) in $CDCl_3$

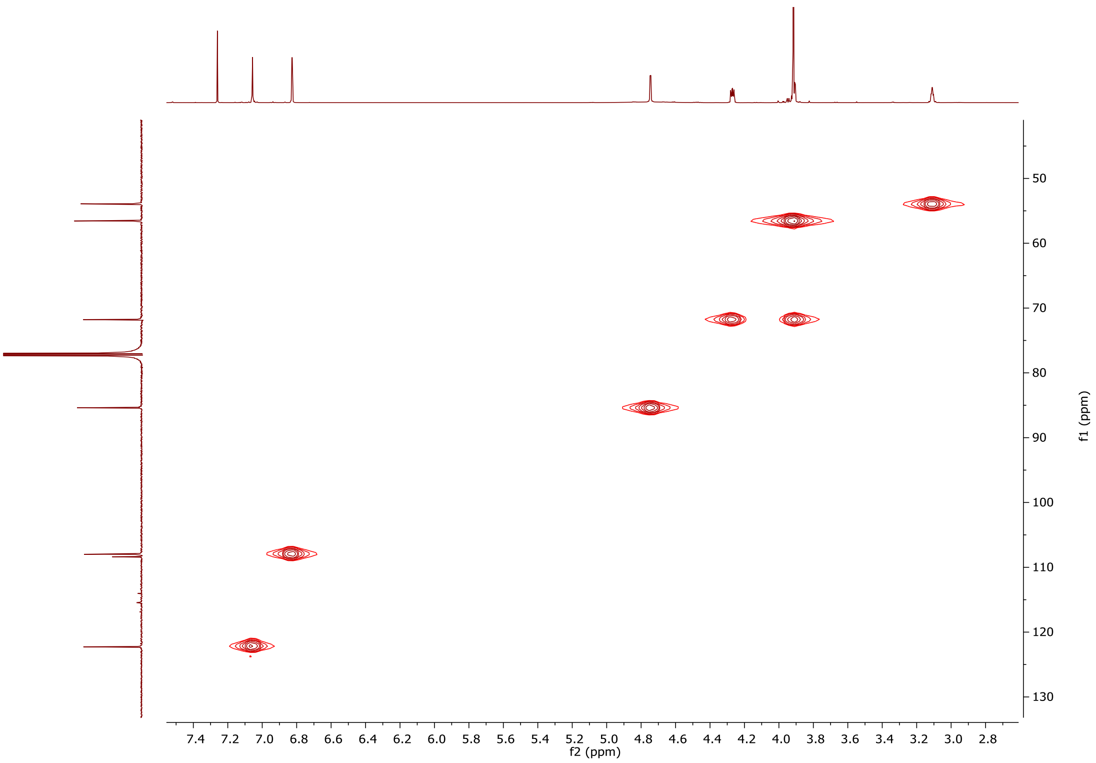

HMBC spectrum of (+)-5,5'-dibromopinoresinol (**5**) in $CDCl_3$

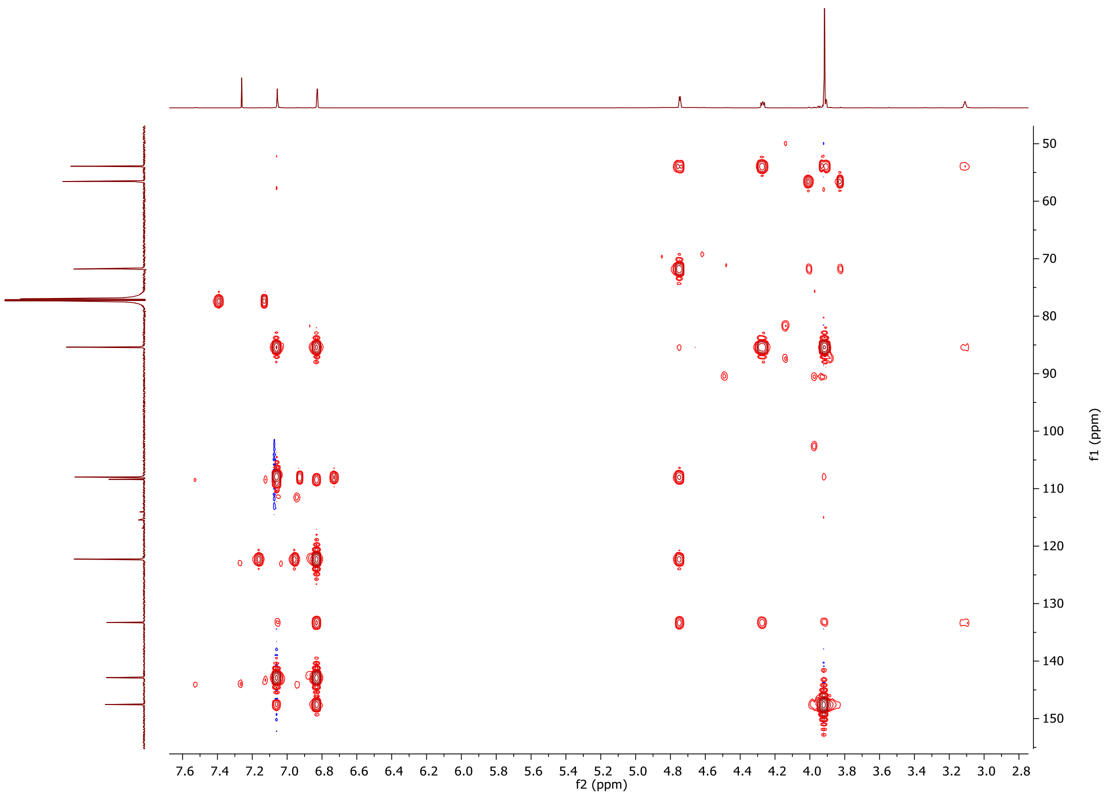

ROESY spectrum of (+)-5,5'-dibromopinoresinol (**5**) in $CDCl_3$

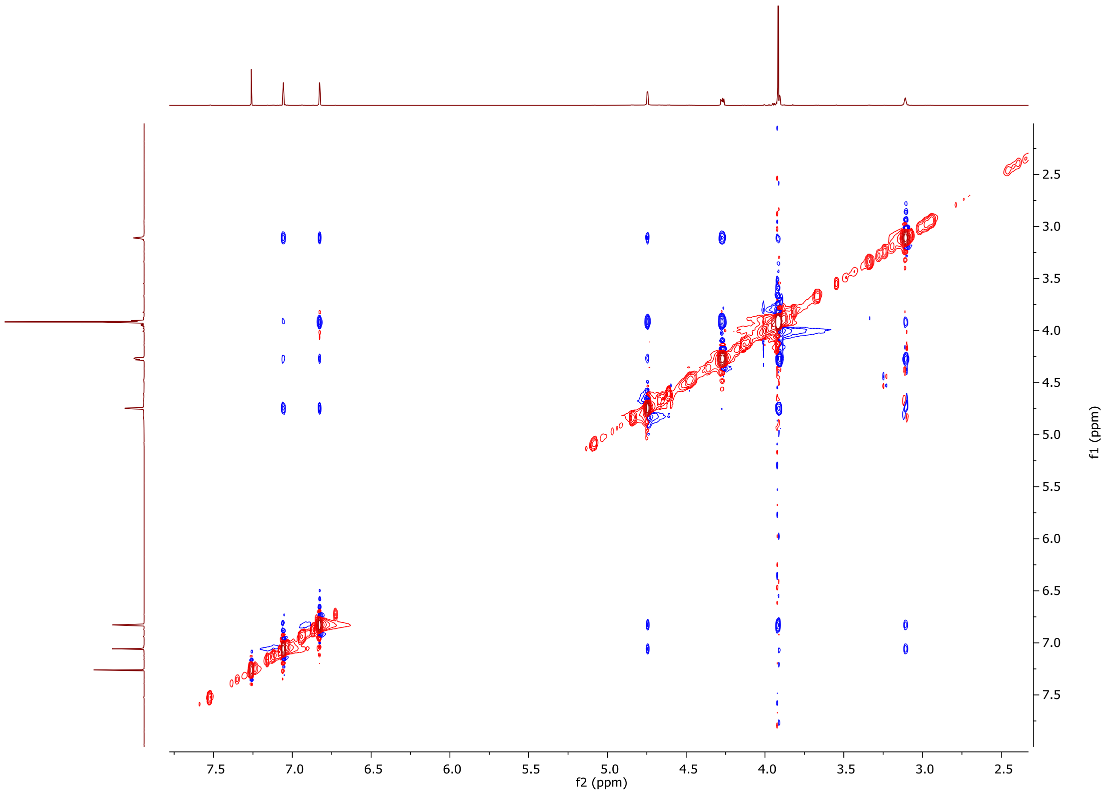

NMR data for (+)-4,4'-di(3,3-dimethylbutanoyl)pinoresinol (**6**) in $CDCl_3$[a]

Position	δ_H, mult (*J* in Hz)	δ_C, type	COSY	HMBC	ROESY
1		139.9, C			
2	6.98 (d, 1.9)	110.0, CH	6	4,6,7	3-OMe,7,8
3		151.4, C			
3-OMe	3.83 (s)	55.9, CH_3		3	2
4		139.3, C			
5	6.99 (d, 8.1)	123.0, CH	6	1,3	
6	6.88 (dd, 8.1, 1.9)	118.0, CH	2,5	2,4,7	7,8,9a
7	4.80 (d, 4.3)	85.7, CH	8	1,2,6,8,9	2,6,8,9b
8	3.09 (m)	54.4, C	7, 9a, 9b	1,7,7',8',9	2,6,7,9a,9b
9a	4.28 (m)	72.0, CH_2	8, 9b	7,8,7',8'	6,8
9b	3.93 (dd, 9.3,3.6)		8, 9a	7,8,7',8'	2,6,7
10		170.5, C			
11	2.46 (s)	47.7, CH_2		10,12,13	13
12		31.2, C			
13	1.14 (s)	29.7, CH_3		11,12,13	11
1'		139.9, C			
2'	6.98 (d, 1.9)	110.0, CH	6'	4',6',7'	3'-OMe,7',8'
3'		151.4, C			
3'-OMe	3.83 (s)	55.9, CH_3		3'	2'
4'		139.3, C			
5'	6.99 (d, 8.1)	123.0, CH	6’	1',3'	
6'	6.88 (dd, 8.1, 1.9)	118.0, CH	2',5’	2',4',7'	7',8',9'a
7'	4.80 (d, 4.3)	85.7, CH	8'	1',2',6',8',9'	2',6',8', 9'a,9'b
8'	3.09 (m)	54.4, C	7', 9'a, 9'b	1',7',7,8,9	2',6',7'
9'a	4.28 (m)	72.0, CH_2	8', 9'b	7',8',7,9	6',8'
9'b	3.93 (dd, 9.3,3.6)		8', 9'a	7',8',7,8	2',6',7'
10'		170.5, C			
11'	2.46 (s)	47.7, CH_2		10',12',13'	13'
12'		31.2, C			
13'	1.14 (s)	29.7, CH_3		11',12',13'	11'

[a]Spectra recorded at 25 °C (800 MHz for ^1H and 200 MHz for ^{13}C).

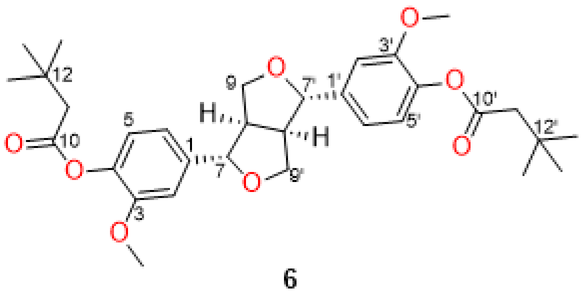

6

^{1}H NMR spectrum of (+)-4,4'-di(3,3-dimethylbutanoyl)pinoresinol (**6**) in $CDCl_3$

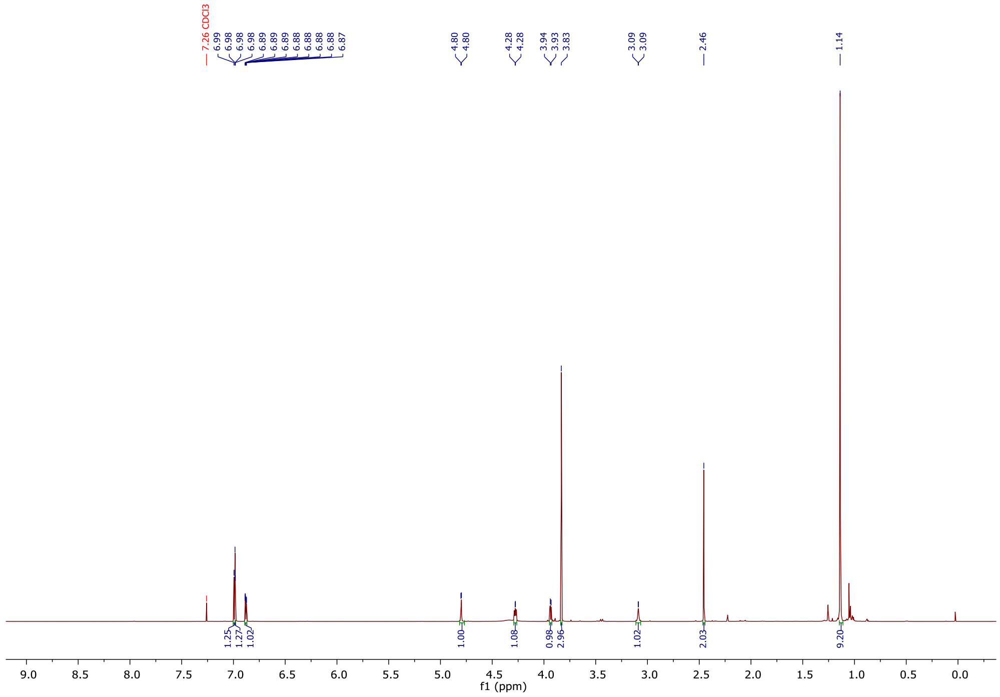

^{13}C NMR spectrum of (+)-4,4'-di(3,3-dimethylbutanoyl)pinoresinol (**6**) in $CDCl_3$

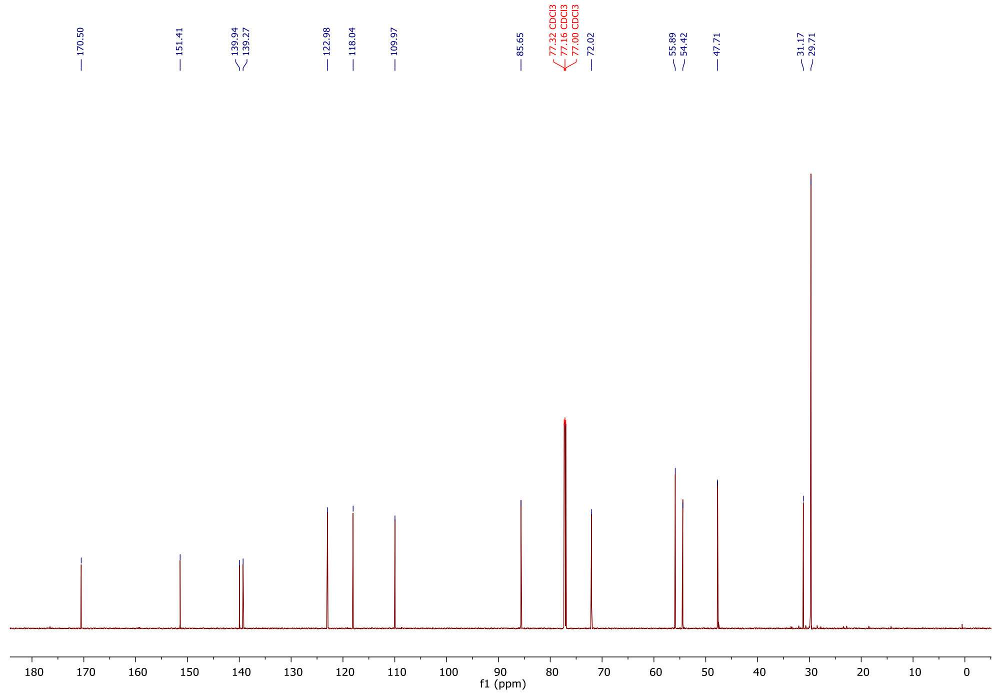

COSY spectrum of (+)-4,4'-di(3,3-dimethylbutanoyl)pinoresinol (**6**) in $CDCl_3$

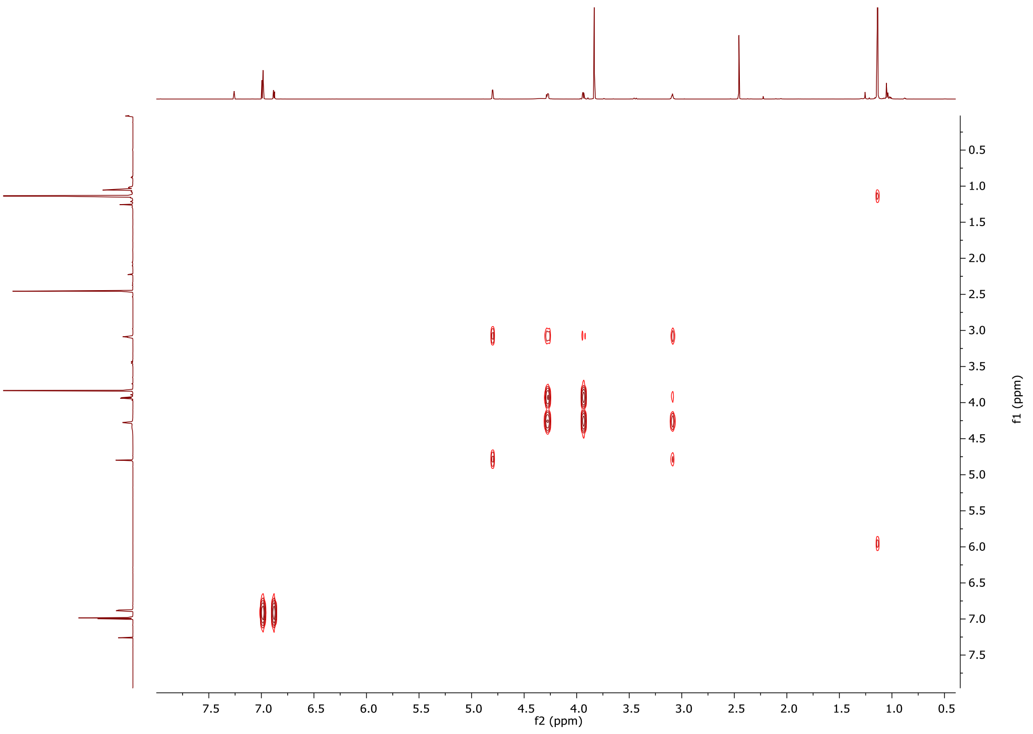

HSQC spectrum of (+)-4,4'-di(3,3-dimethylbutanoyl)pinoresinol (**6**) in $CDCl_3$

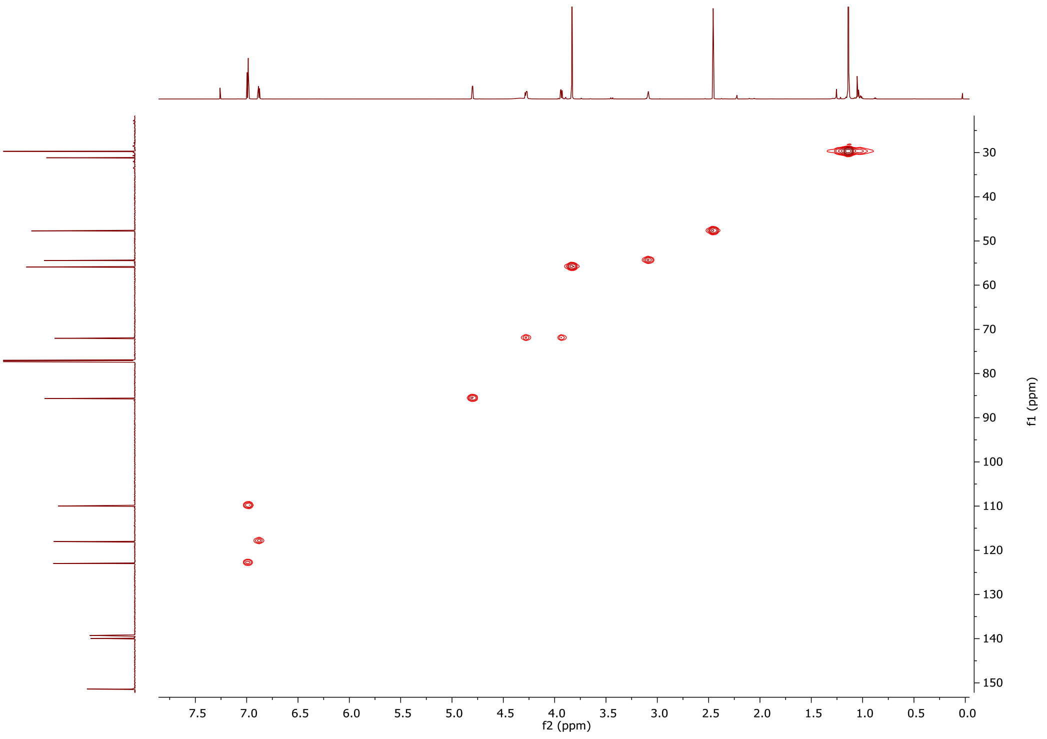

HMBC spectrum of (+)-4,4'-di(3,3-dimethylbutanoyl)pinoresinol (**6**) in $CDCl_3$

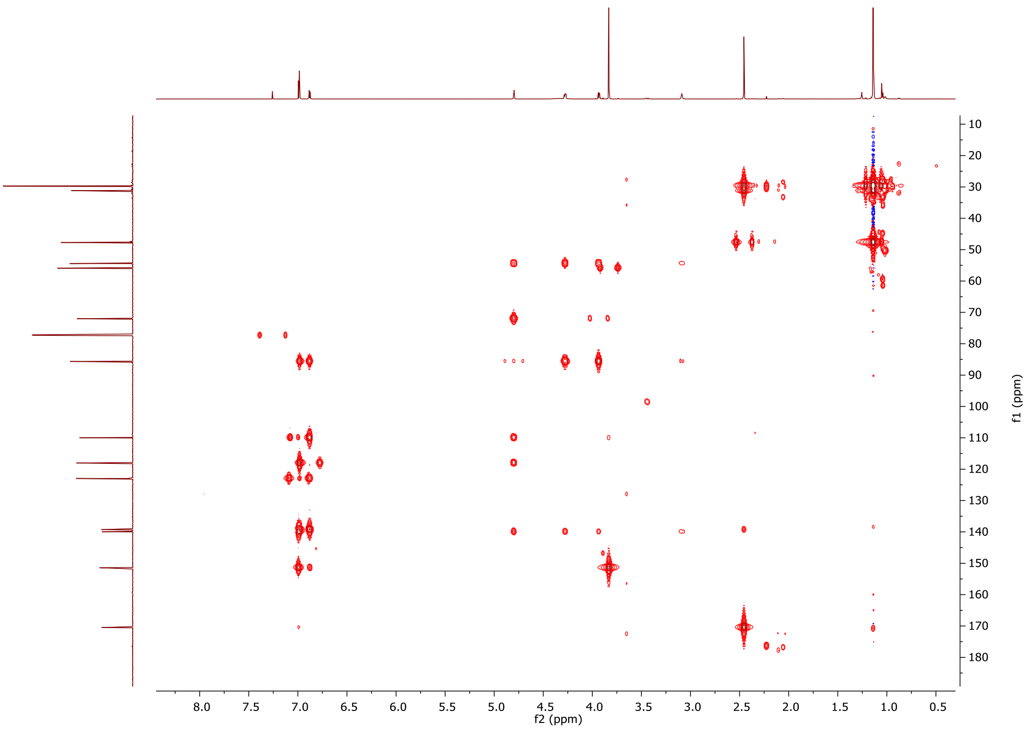

ROESY spectrum of (+)-4,4'-di(3,3-dimethylbutanoyl)pinoresinol (**6**) in $CDCl_3$

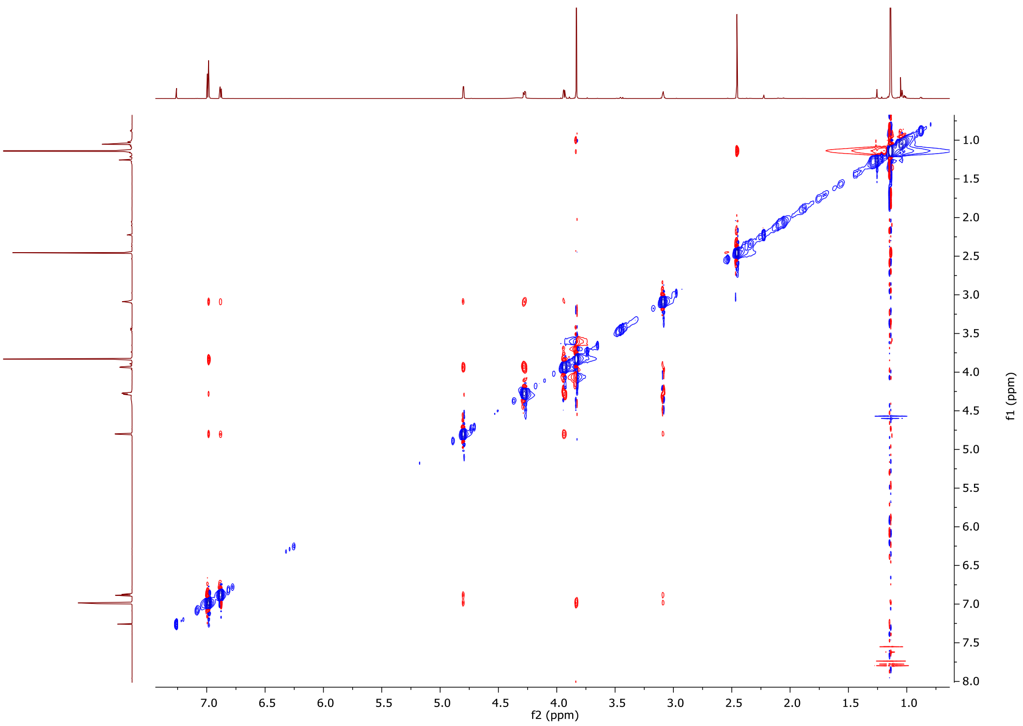

NMR data for (+)-4,4'-dipivaloylpinoresinol (**7**) in $CDCl_3$[a]

Position	δ_H, mult (*J* in Hz)	δ_C, type	COSY	HMBC	ROESY
1		139.8, C			
2	6.97 (d, 1.9)	110.1, CH	6	4,6,7	3-OMe,7, 8
3		151.5, C			
3-OMe	3.82 (s)	56.1, CH_3		2^w,3	2
4		139.7, C			
5	6.98 (d, 8.3)	122.8, CH	6	1,3	
6	6.88 (dd, 8.3, 1.9)	118.1, CH	2,5	2,4,7	7,8,9a
7	4.80 (d, 4.3)	85.7, CH	2,6,8	1,2,6,8,9	2,6,8,9b
8	3.10 (m)	54.5, CH	7, 9a, 9b	1,7,7',8'	2,6,9a
9a	4.28 (m)	72.0, CH_2	8, 9b	1,7,8,2'	6,8,9b
9b	3.94 (dd, 9.4, 3.6)		8, 9a	1,7,8,2'	7,9a
10		177.0, C			
11		39.2, C			
12	1.36 (s)	27.4, CH_3		10, 11,12	
1'		139.8, C			
2'	6.98 (d, 1.9)	110.1, CH	6’	4',6',7'	3-OMe,7, 8
3'		151.5, C			
3'-OMe	3.82 (s)	56.1, CH_3		$2'^w$,3'	2'
4'		139.7, C			
5'	6.97 (d, 8.3)	122.8, CH	6’	1',3'	
6'	6.88 (dd, 8.3, 1.9)	118.1, CH	2’,5’	2',4',7'	7',8',9'a
7'	4.80 (d, 4.3)	85.7, CH	2',6',8'	1',2',6',8',9'	2',6',8',9'b
8'	3.10 (m)	54.5, CH	7', 9'a, 9'b	1',7',7,8	2',6',9'a
9'a	4.28 (m)	72.0, CH_2	8', 9'b	1',7',8',2	6',8', 9'b
9'b	3.94 (dd, 9.4, 3.6)		8', 9'a	1',7',8',2	7',9'a
10'		177.0, C			
11'		39.2, C			
12'	1.36 (s)	27.4, CH_3		10', 11',12'	

[a]Spectra recorded at 25 °C (800 MHz for ^1H and 200 MHz for ^{13}C); [w]weak correlation.

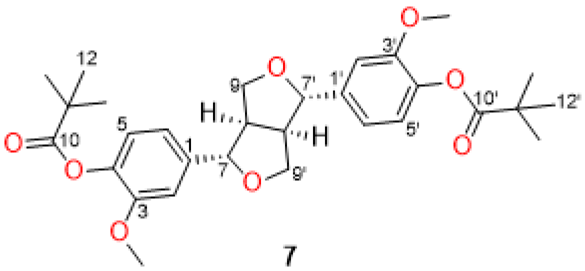

7

^{1}H NMR spectrum of (+)-4,4'-dipivaloylpinoresinol (**7**) in $CDCl_3$

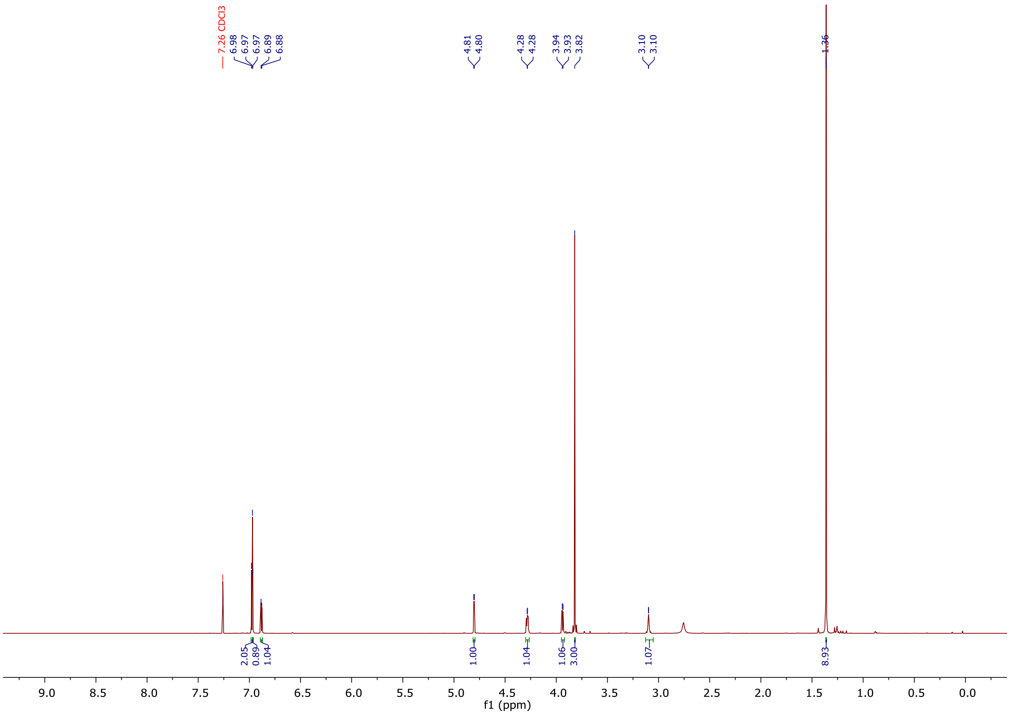

^{13}C NMR spectrum of (+)-4,4'-dipivaloylpinoresinol (**7**) in $CDCl_3$

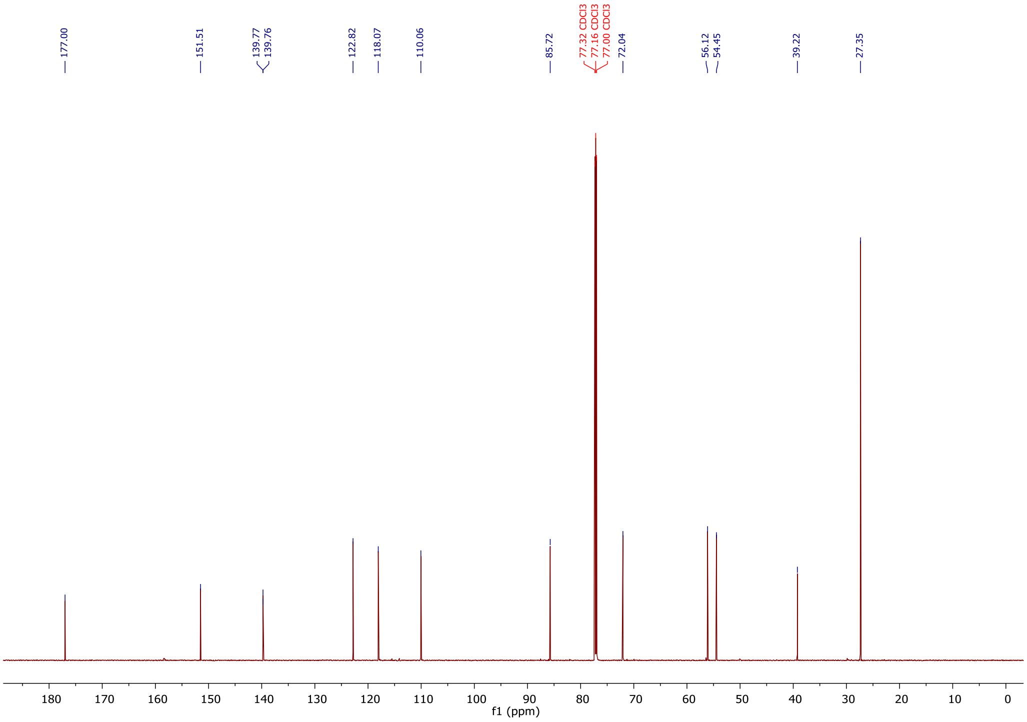

COSY spectrum of (+)-4,4'-dipivaloylpinoresinol (**7**) in $CDCl_3$

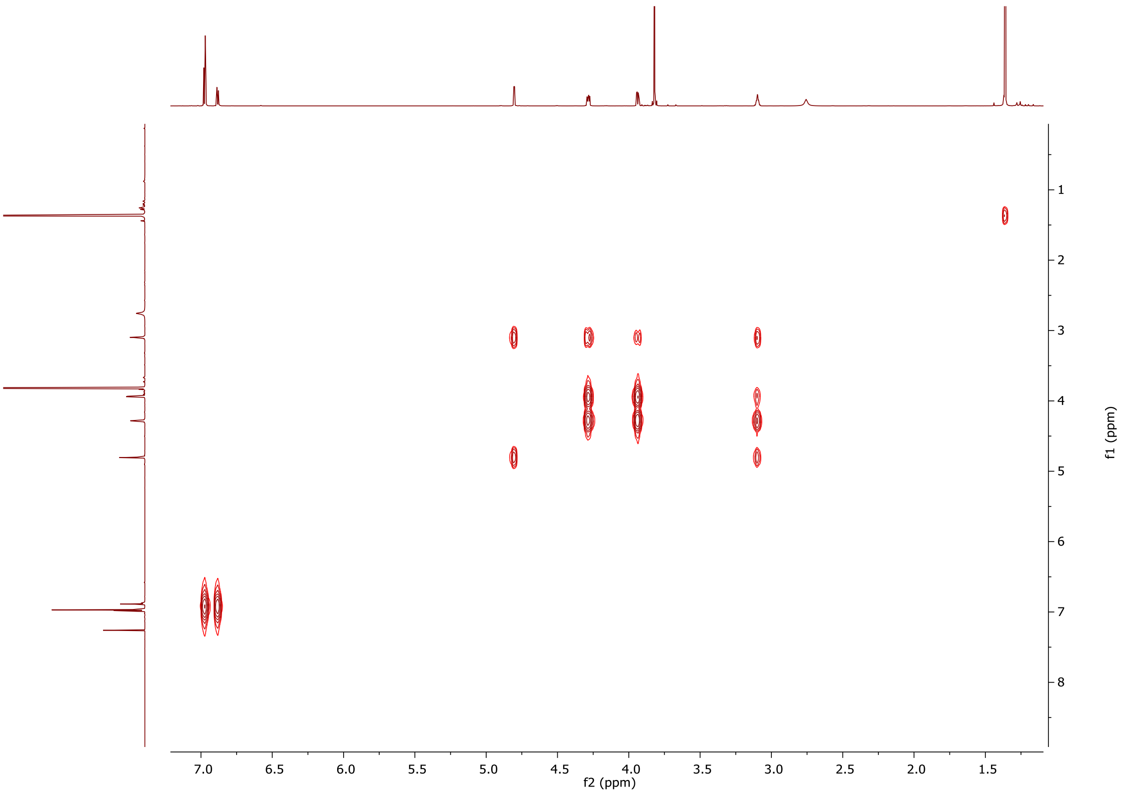

HSQC spectrum of (+)-4,4'-dipivaloylpinoresinol (**7**) in $CDCl_3$

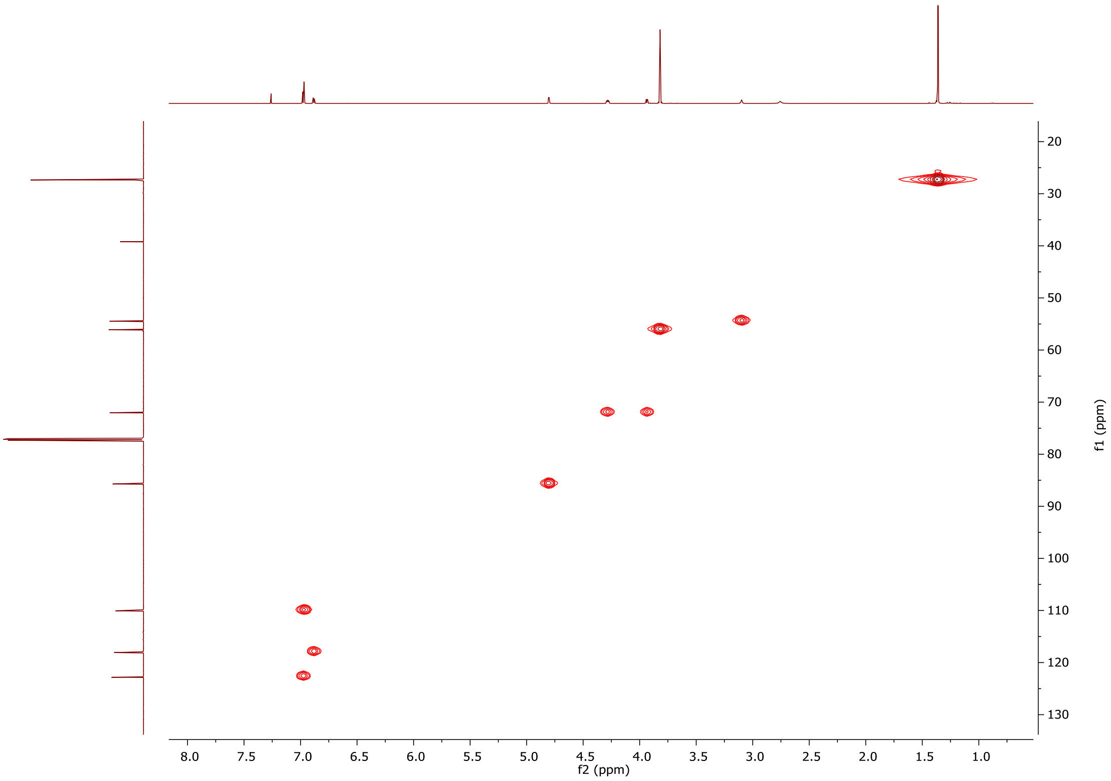

HMBC spectrum of (+)-4,4'-dipivaloylpinoresinol (**7**) in $CDCl_3$

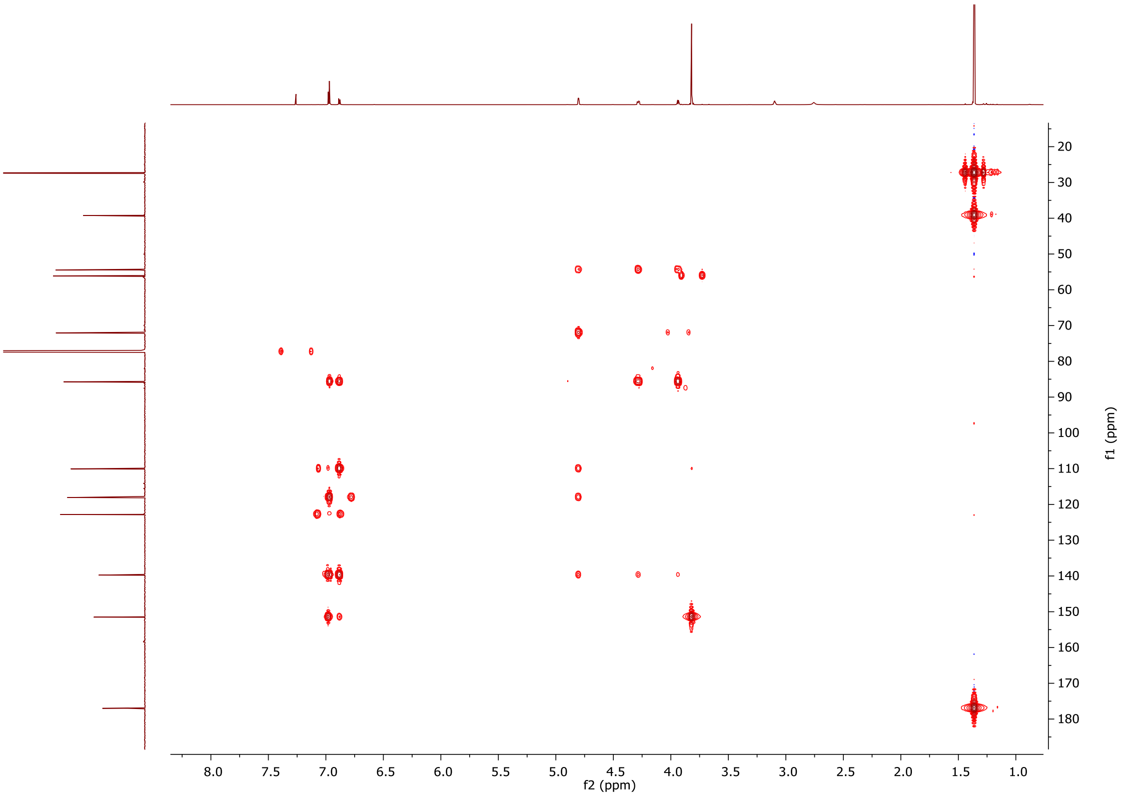

ROESY spectrum of (+)-4,4'-dipivaloylpinoresinol (**7**) in $CDCl_3$

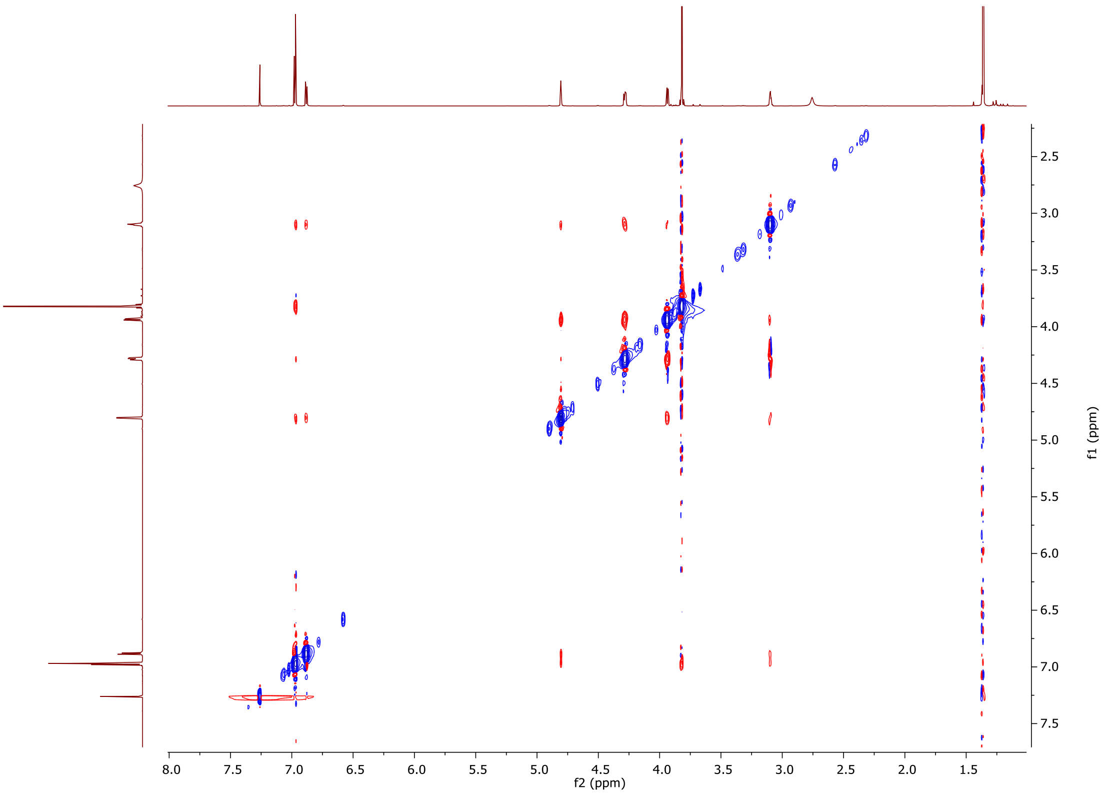

UHPLC–MS data of salicifoliol (**1**)

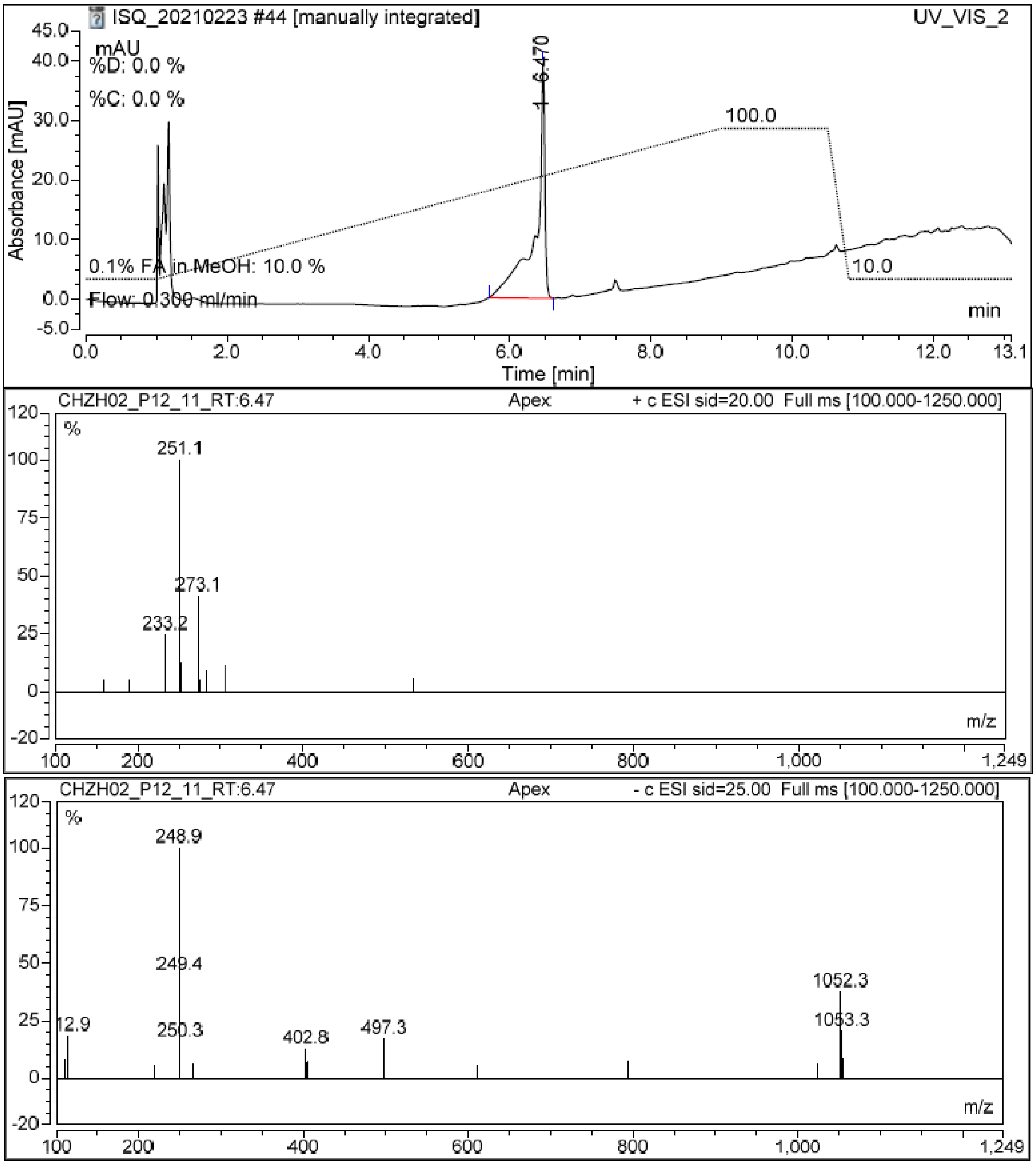

UHPLC–MS data of (+)-pinoresinol (**2**)

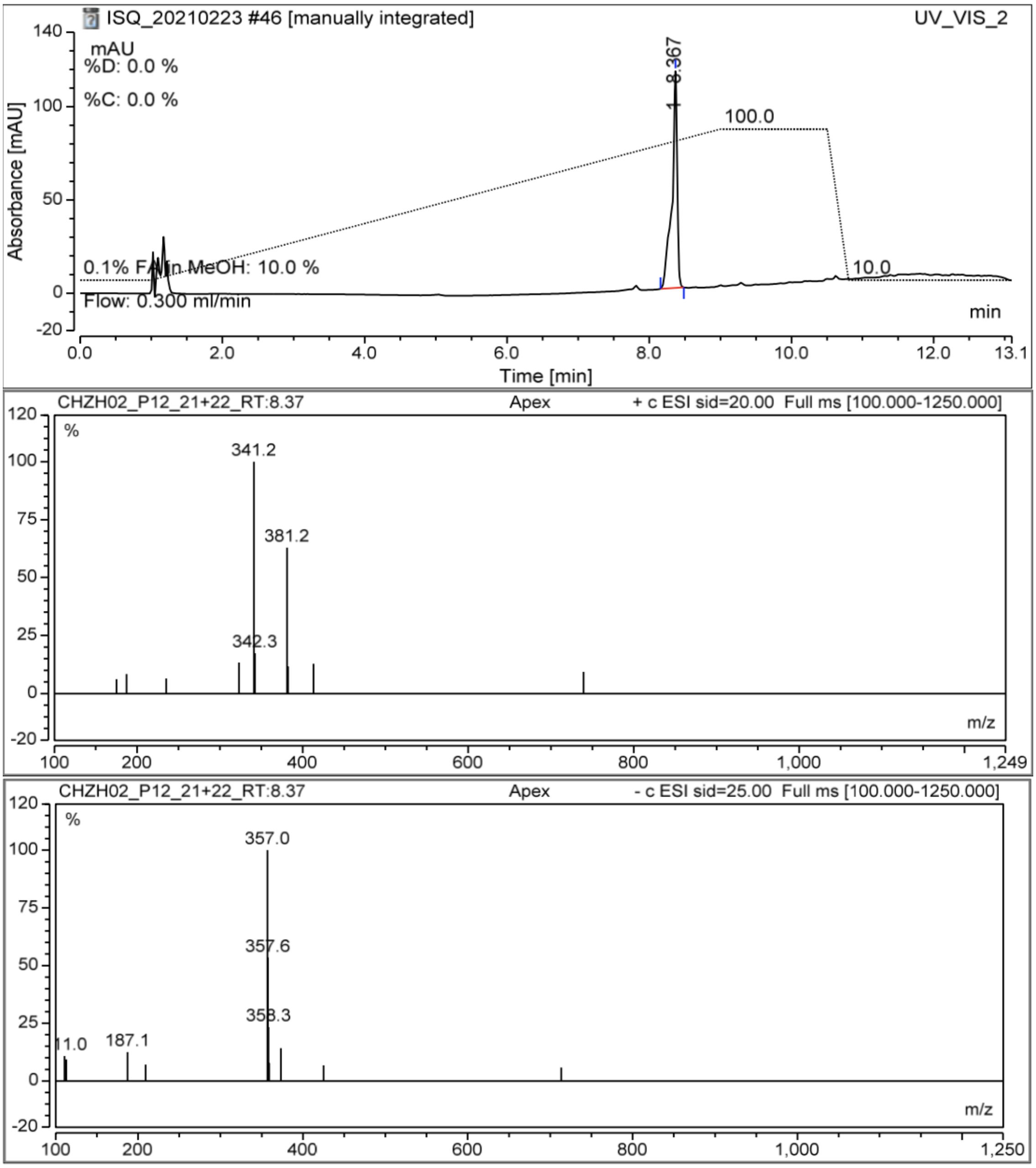

UHPLC–MS data of (+)-eudesmin (**3**)

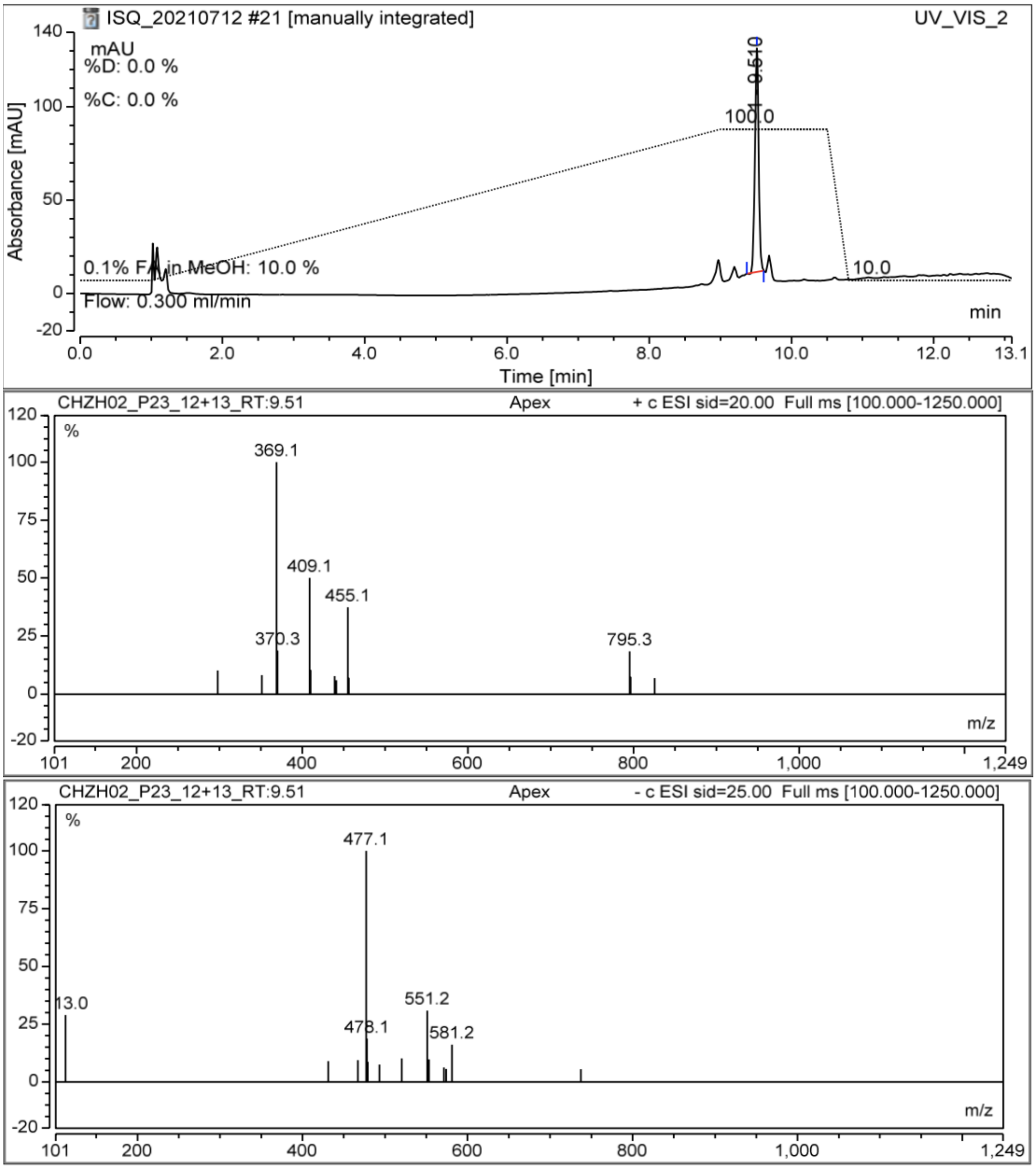

UHPLC–MS data of (+)-phillygenin (**4**)

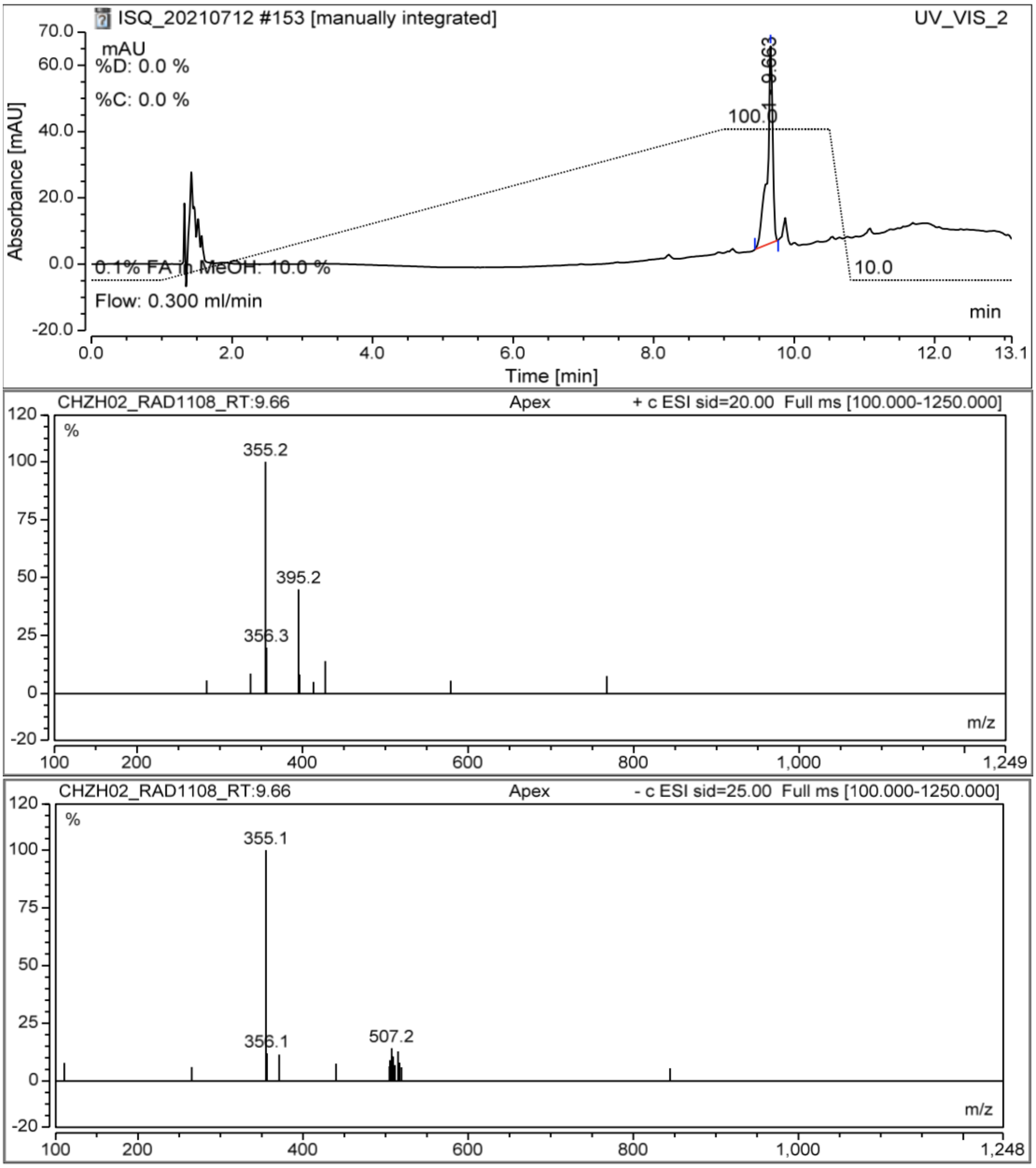

UHPLC–MS data of (+)-5,5'-dibromopinoresinol (**5**)

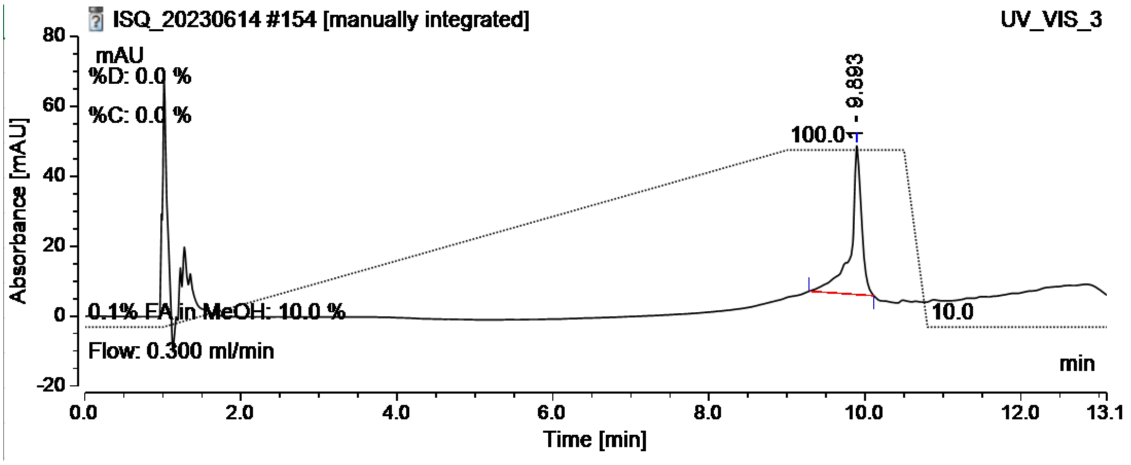

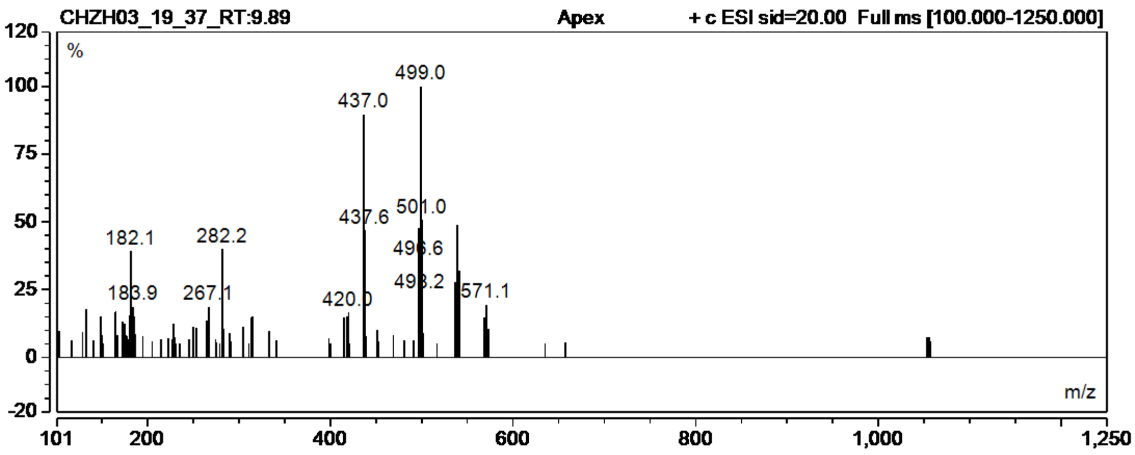

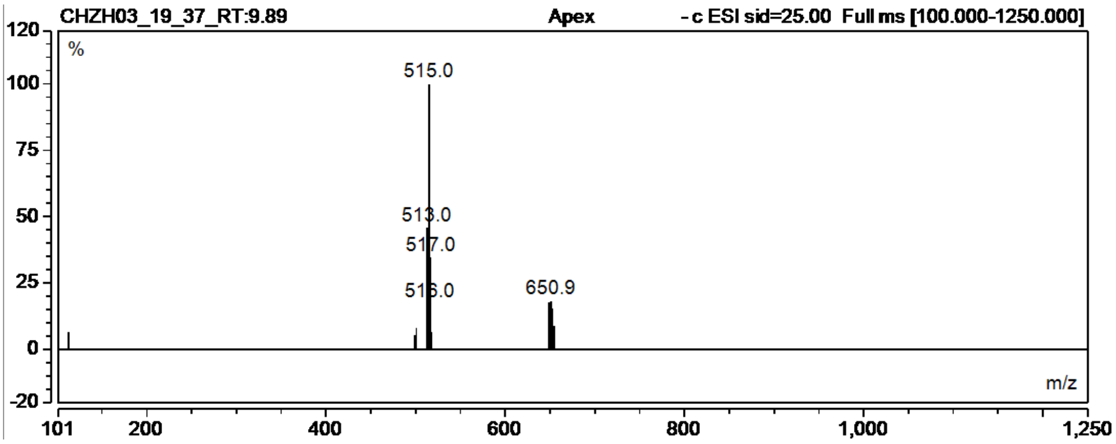

UHPLC–MS data of (+)-4,4'-di(3,3-dimethylbutanoyl)pinoresinol (**6**)

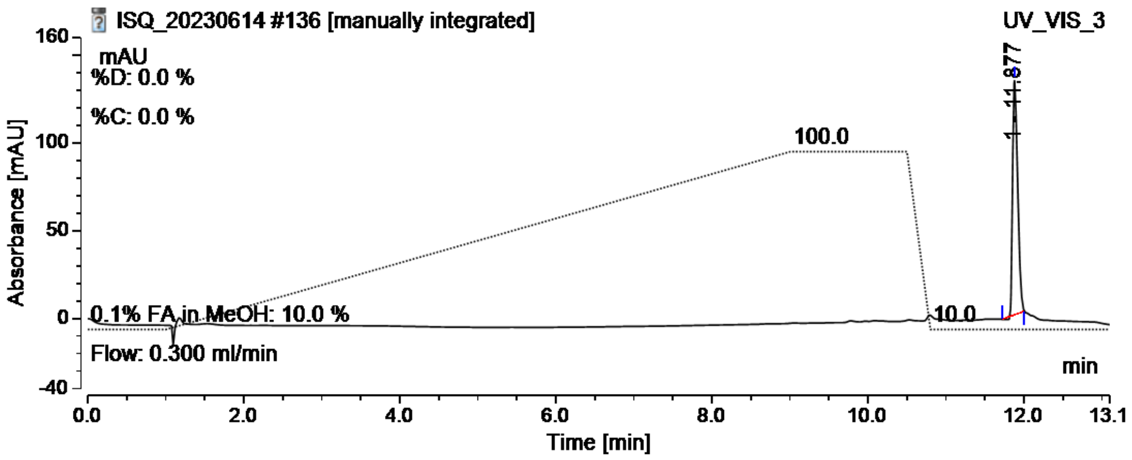

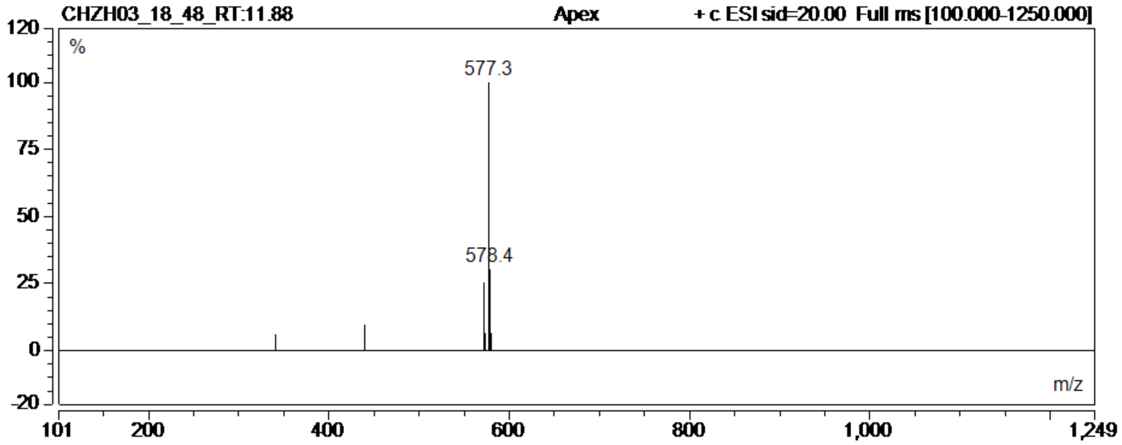

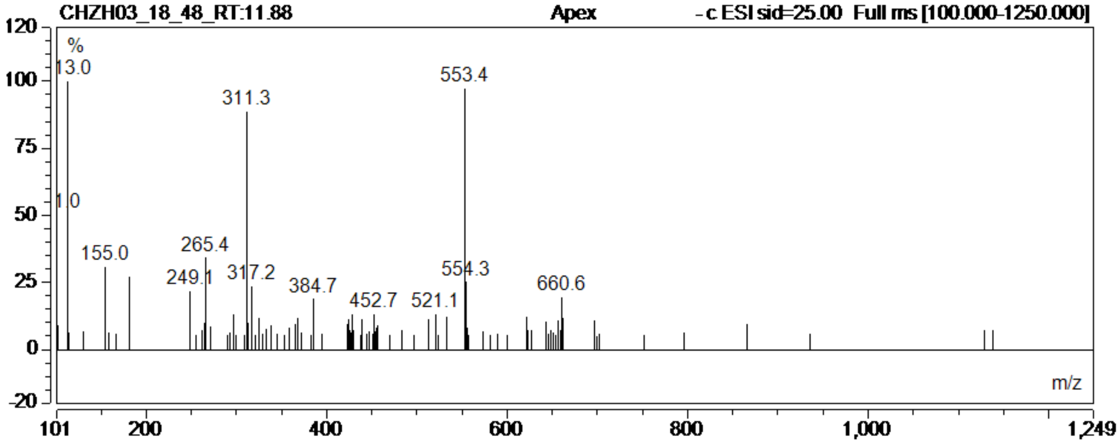

UHPLC–MS data of (+)-4,4'-dipivaloylpinoresinol (**7**)

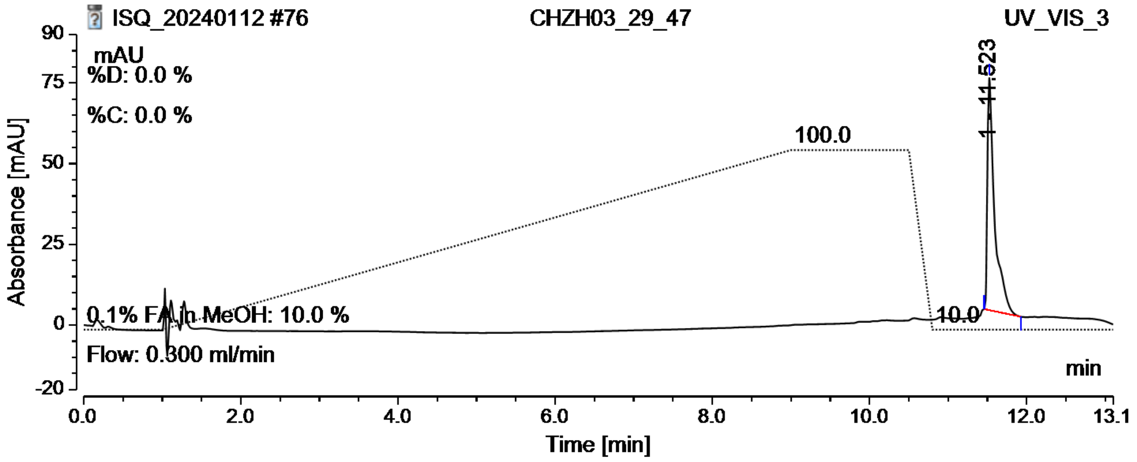

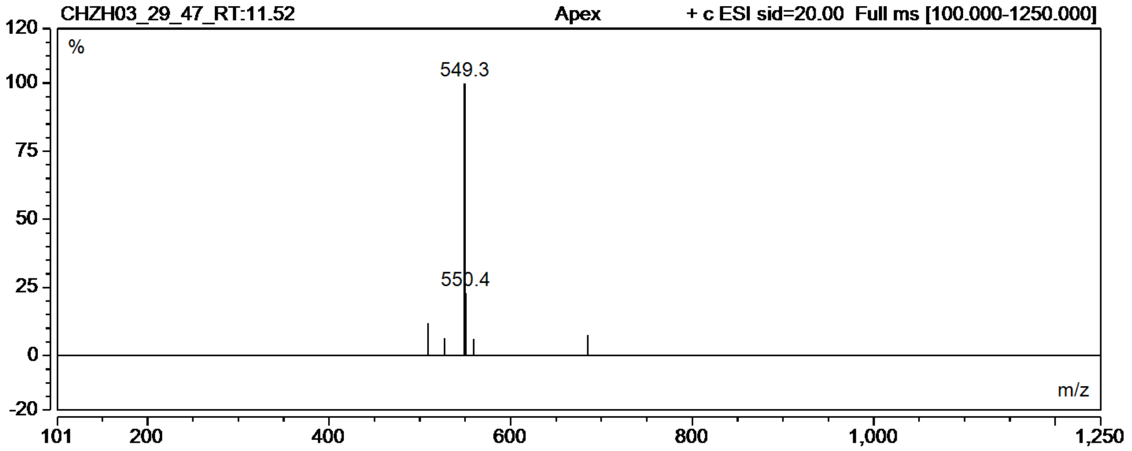

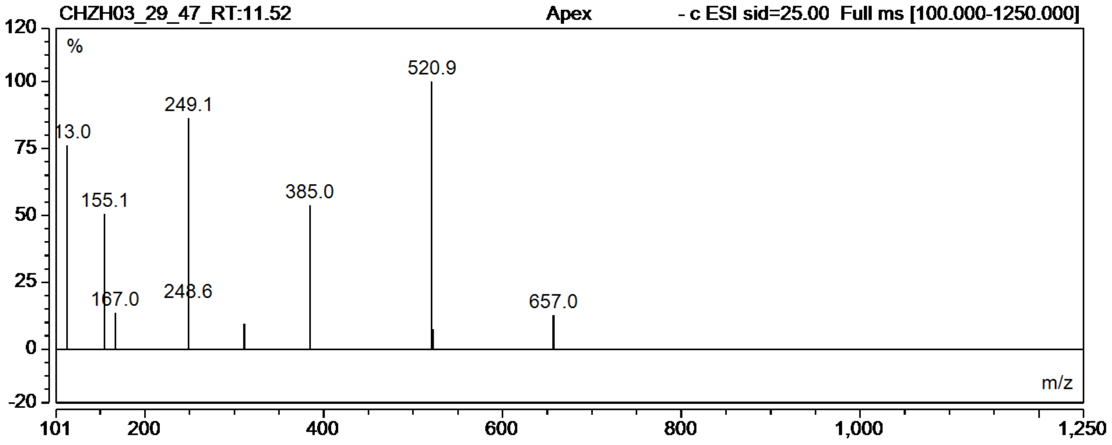

HRESIMS of (+)-pinoresinol (**2**)

Mass Spectrum SmartFormula Report

Analysis Info

Analysis Name	D:\Data\Wendy\20211109\RAD1105_000001.d	Acquisition Date	9/11/21 9:38:46 AM
Method	lmw_low_DI_20211027	Operator	
Sample Name	RAD1105	Instrument	solariX XR
Comment			

Acquisition Parameter

Acquisition Mode	Single MS	Acquired Scans	8	Calibration Date	Wed Oct 27 11:09:46
n/a	n/a	No. of Cell Fills	1	Data Acquisition Size	1048576
Broadband Low Mass	98.3 m/z	n/a	n/a	Data Processing Size (SI)	2097152
Broadband High Mass	2000.0 m/z	n/a	n/a	Apodization	Full-Sine
Source Accumulation	0.000 sec	n/a	n/a		
Ion Accumulation Time	0.300 sec				

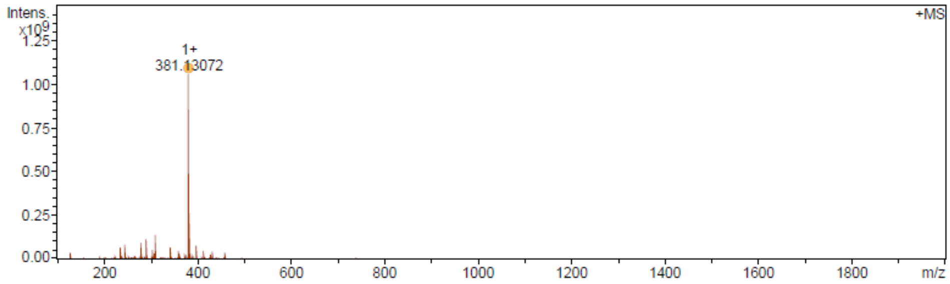

Meas. m/z	#	Ion Formula	Score	m/z	err [ppm]	Mean err [ppm]	mSigma	rdb	e⁻ Conf	N-Rule
381.130717	1	C21H18N4NaO2	72.04	381.132197	3.9	2.8	12.0	15.0	even	ok
	2	C20H22NaO6	100.00	381.130859	0.4	-0.4	17.3	10.0	even	ok

HRESIMS of (+)-eudesmin (**3**)

Griffith UNIVERSITY

High Resolution Mass Spectrum

Analysis Info

Analysis Name	D:\Data\Chen\20220808\RAD1106000001.d		
Method	DirectInfusion_2018_pos.m	Acquisition Date	8/8/2022 4:28:02 PM
Sample Name	RAD1106	Instrument	maXis II ETD 1823391.22321
Comment			

+MS, 0.1-0.1min #5-6

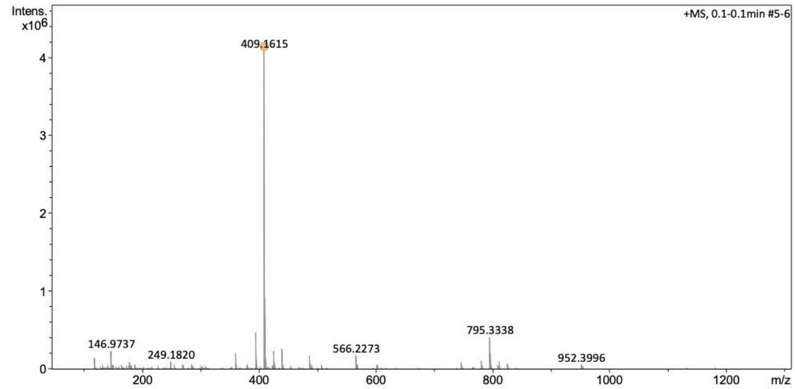

Meas. m/z	#	Ion Formula	m/z	err [ppm]	mSigma	# mSigma	Score	rdb	e⁻ Conf	N-Rule
409.1615	1	C22H26NaO6	409.1622	1.6	9.3	1	100.00	9.5	even	ok

HRESIMS of (+)-phillygenin (**4**)

Mass Spectrum SmartFormula Report

Analysis Info

Analysis Name	D:\Data\Sasha\APRIL 2024\RAD1108000001.d	Acquisition Date	4/9/2024 10:11:13 AM
Method	DirectInfusion_2018_pos.m	Operator	Demo User
Sample Name	RAD1108	Instrument	maXis II ETD
Comment			

Acquisition Parameter

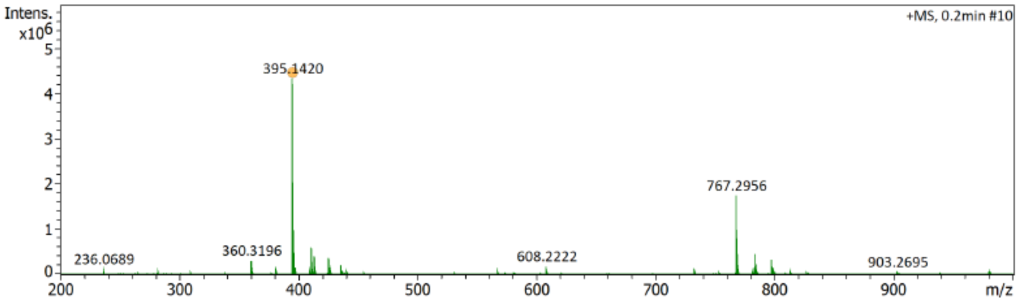

Meas. m/z	#	Ion Formula	m/z	err [ppm]	mSigma	# mSigma	Score	rdb	e⁻ Conf	N-Rule
395.1420	1	C21H24NaO6	395.1465	11.4	1.8	1	100.00	10.0	even	ok

HRESIMS of (+)-5,5'-dibromopinoresinol (**5**)

Mass Spectrum SmartFormula Report

Analysis Info

		Acquisition Date	5/21/2024 1:18:18 PM
Analysis Name	D:\Data\Sasha\May 2024\RAD1137000001.d		
Method	DirectInfusion_2018_pos.m	Operator	Demo User
Sample Name	RAD1137	Instrument	maXis II ETD
Comment			

Acquisition Parameter

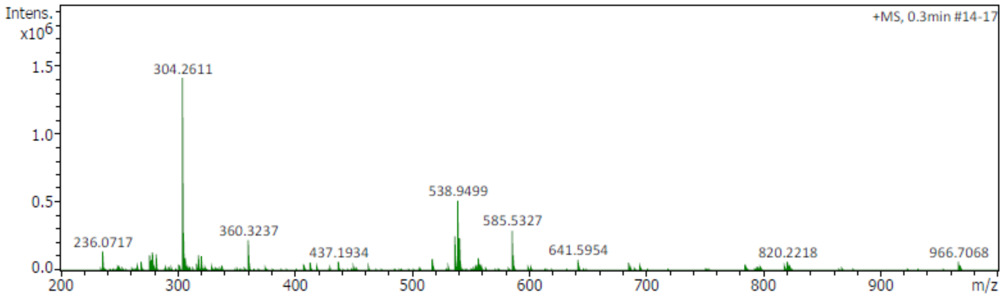

Meas. m/z	#	Ion Formula	m/z	err [ppm]	mSigma	# mSigma	Score	rdb	e⁻ Conf	N-Rule
536.9519	1	C20H20Br2NaO6	536.9519	0.0	25.4	1	100.00	14.0	even	ok

HRESIMS of (+)-4,4'-di(3,3-dimethylbutanoyl)pinoresinol (**6**)

Mass Spectrum SmartFormula Report

Analysis Info

Analysis Name	D:\Data\Sasha\May 2024\RAD1136000001.d	Acquisition Date	5/21/2024 1:26:45 PM
Method	DirectInfusion_2018_pos.m	Operator	Demo User
Sample Name	RAD1136	Instrument	maXis II ETD
Comment			

Acquisition Parameter

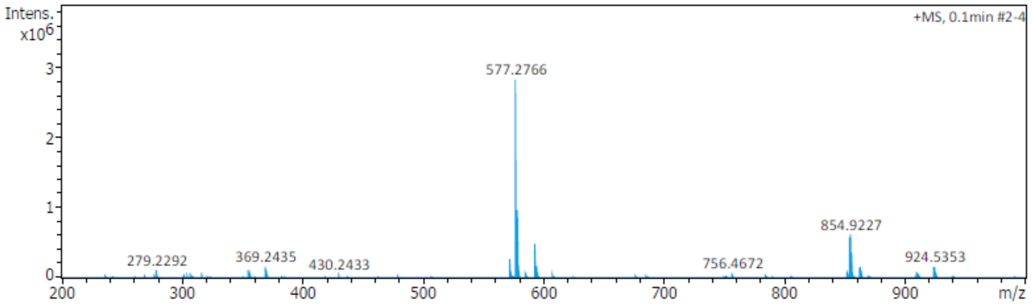

Meas. m/z	#	Ion Formula	m/z	err [ppm]	mSigma	# mSigma	Score	rdb	e⁻ Conf	N-Rule
577.2766	1	C32H42NaO8	577.2772	1.0	6.6	2	100.00	12.0	even	ok

HRESIMS of (+)-4,4'-dipivaloylpinoresinol (**7**)

Mass Spectrum SmartFormula Report

Analysis Info

Analysis Name	D:\Data\Sasha\May 2024\RAD1138000001.d	Acquisition Date	5/21/2024 1:08:27 PM
Method	DirectInfusion_2018_pos.m	Operator	Demo User
Sample Name	RAD1138	Instrument	maXis II ETD
Comment			

Acquisition Parameter

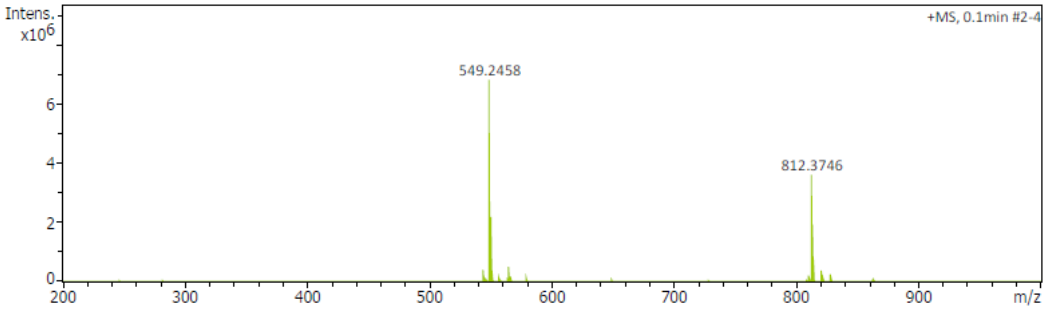

Meas. m/z	#	Ion Formula	m/z	err [ppm]	mSigma	# mSigma	Score	rdb	e⁻ Conf	N-Rule
549.2458	1	C30H38NaO8	549.2459	0.1	7.5	2	100.00	12.0	even	ok

NMR data comparison for (+)-5,5'-dibromopinoresinol (**5**) and previously synthesised and reported racemic (±)-5,5'-dibromopinoresinol in acetone-d_6

Position	δ_H (mult, *J* in Hz) 800 MHz data, Zhang (2026) et al	δ_H (mult, *J* in Hz) 500 MHz data, Yue (2021) et al
1		
2	7.00 (d, 1.8)	6.99 (d, 1.86)
3		
3-OMe	3.87 (s)	3.85 (s)
4		
4-OH	8.22 (s)	[a]
5		
6	7.12 (dd, 1.8, 0.6)	7.11 (dd, 1.88, 0.51)
7	4.70 (d, 4.10)	4.69 (d, 4.10)
8	3.11 (m)	3.10 (m)
9a	4.23 (m)	4.22 (m)
9b	3.85 (dd 9.3, 3.6)	3.84 (d, 3.68)
1'		
2'	7.00 (d, 1.8)	6.99 (d, 1.86)
3'		
3'-OMe	3.87 (s)	3.85 (s)
4'		
4'-OH	8.22 (s)	[a]
5'		
6'	7.12 (dd, 1.8, 0.6)	7.11 (dd, 1.88, 0.51)
7'	4.70 (d, 4.10)	4.69 (d, 4.10)
8'	3.11 (m)	3.10 (m)
9'a	4.23 (m)	4.22 (m)
9'b	3.85 (dd 9.3, 3.6)	3.84 (d, 3.68)

[a]Signal not observed.

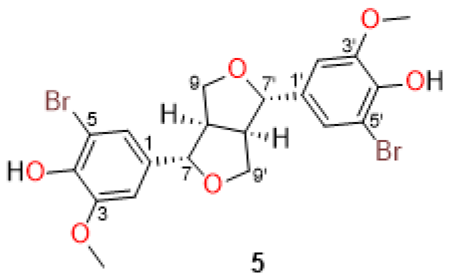

5

^{1}H NMR spectrum of (+)-5,5'-dibromopinoresinol (**5**) in acetone-d_6

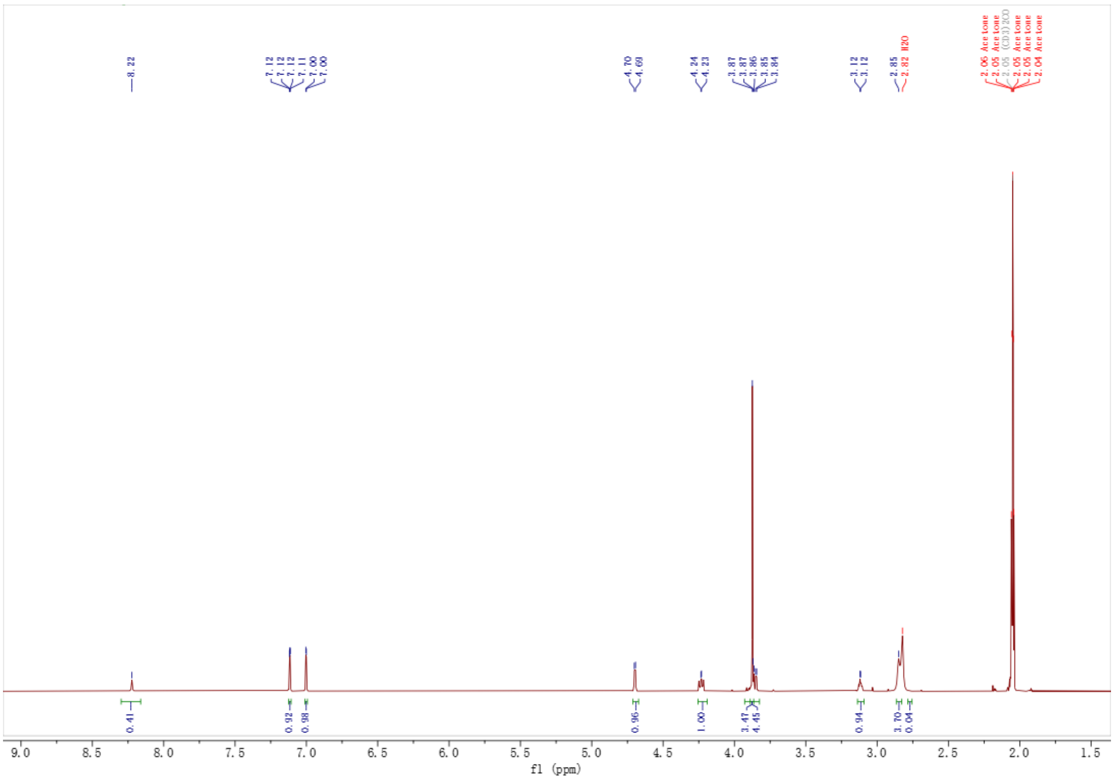

Supplement: File 1 — Compound characterization data. [file Beilstein_J_Org_Chem-22-691-s001.pdf]
